# Mapping the Nicotinic Acetylcholine Receptor Nanocluster Topography at the Cell Membrane with STED and STORM Nanoscopies

**DOI:** 10.3390/ijms231810435

**Published:** 2022-09-09

**Authors:** Lucas A. Saavedra, Héctor Buena-Maizón, Francisco J. Barrantes

**Affiliations:** Laboratory of Molecular Neurobiology, Biomedical Research Institute (BIOMED), UCA–CONICET, Av. Alicia Moreau de Justo 1600, Buenos Aires C1107AFF, Argentina

**Keywords:** nicotinic acetylcholine receptor, super-resolution microscopy, nanoscopy, STED, STORM, cholesterol, nanoclusters

## Abstract

The cell-surface topography and density of nicotinic acetylcholine receptors (nAChRs) play a key functional role in the synapse. Here we employ in parallel two labeling and two super-resolution microscopy strategies to characterize the distribution of this receptor at the plasma membrane of the mammalian clonal cell line CHO-K1/A5. Cells were interrogated with two targeted techniques (confocal microscopy and stimulated emission depletion (STED) nanoscopy) and single-molecule nanoscopy (stochastic optical reconstruction microscopy, STORM) using the same fluorophore, Alexa Fluor 647, tagged onto either α-bungarotoxin (BTX) or the monoclonal antibody mAb35. Analysis of the topography of nanometer-sized aggregates (“nanoclusters”) was carried out using STORMGraph, a quantitative clustering analysis for single-molecule localization microscopy based on graph theory and community detection, and ASTRICS, an inter-cluster similarity algorithm based on computational geometry. Antibody-induced crosslinking of receptors resulted in nanoclusters with a larger number of receptor molecules and higher densities than those observed in BTX-labeled samples. STORM and STED provided complementary information, STED rendering a direct map of the mesoscale nAChR distribution at distances ~10-times larger than the nanocluster centroid distances measured in STORM samples. By applying photon threshold filtering analysis, we show that it is also possible to detect the mesoscale organization in STORM images.

## 1. Introduction

The spatial organization of membrane proteins is intimately linked to their functional properties, and this is particularly relevant in the case of ligand-gated receptors, where the signaling efficacy of the external ligand is tightly coupled to the supramolecular organization of the receptor molecules. For this reason, the characterization of the supramolecular topography of the ubiquitous neurotransmitter for acetylcholine, the nicotinic acetylcholine receptor (nAChR), and the factors that regulate its topography in the membrane are important areas of research for understanding the functional and pathophysiological consequences of receptor distribution. At the mature cholinergic synapse, the neuromuscular junction (NMJ), nAChRs are organized in the form of a single 2-dimensional supramolecular complex of quasi-maximal packing density in a small area juxtaposed to the nervous terminal, patched at the extraordinary density of 10,000–20,000 particles/μm^2^; their density falls sharply in the rest of the plasma membrane to less than <100 particles/μm^2^ ([1,2] reviewed in [3]). Synaptic strength and efficacy are to a great extent determined by the number of active nAChR molecules. This, in turn, relies on the equilibrium between two sets of factors: (i) diffusion into and out of the synaptic region from non-synaptic (extrasynaptic) areas [4,5], and (ii) the turnover of receptors at the cell surface [6,7,8,9], determined by the rate and extent of biosynthesis and exocytic delivery to the plasmalemma on the one hand, and removal of surface receptors by internalization (endocytosis) on the other. 

Using stimulated depletion super-resolution microscopy (STED) [10] the nAChR was the first neurotransmitter receptor to be imaged with optical nanoscopy, revealing the occurrence of supramolecular aggregates in the form of clusters of nanometric dimensions [11]. Subsequent studies from our laboratory have employed total internal reflection (TIRF) microscopy and single-particle tracking methods [12] to follow the translational motional regimes of the receptor in live-cell imaging. More recently, single-molecule localization microscopy (SMLM) methods, and in particular stochastic optical reconstruction microscopy (STORM) [13] were applied to describe the heterogeneous and anomalous diffusional properties of the receptor at the cell surface [14,15]. 

Here, we study the cell-surface supramolecular organization of the adult muscle-type nAChR heterologously expressed in a mammalian cell system. We label the nAChR with either a monovalent ligand, fluorescent α-bungarotoxin (αBTX) conjugated with Alexa Fluor 647 or a multivalent ligand, the primary monoclonal antibody mAb35 against the α1 subunit of the receptor, also directly tagged with the same fluorophore, Alexa Fluor 647. The cyanine dye Alexa Fluor 647 is commonly used for STORM imaging applications because of its photo-switchable character and the photostability of its fluorophore [16], but it is not commonly applied in STED microscopy. Here we show that it is possible to successfully use this fluorophore to image the nAChR in fixed cells using STORM and confocal/STED nanoscopies alike. In STORM single-molecule localization microscopy (SMLM), super-resolution images are not directly captured in a single micrograph; instead, their localizations are reconstructed from the stochastic on-off blinking of the fluorophores tagging individual molecules. Some molecules may thus not appear in the reconstruction due to sub-stoichiometric or total lack of labeling, or exhaustion of the fluorophore. In contrast, single molecules may be counted more than once, giving rise to overcounting [17]. The latter would be particularly detrimental to our specific goals, namely determining the topographic distribution and degree of aggregation of individual receptor molecules. STED nanoscopy is a non-stochastic, targeted direct (“what you see is what you get”) method that does not require reconstruction of the localizations via post-acquisition analysis and is therefore not prone to overcounting artifacts [17]. The complementary use of two different super-resolution approaches in tandem provides more solid grounds to validate the occurrence of protein molecular aggregates in cell membranes. For the analysis of the state of aggregation of the nAChR we apply STORMGraph, an automated quantitative clustering method developed for SMLM and based on graph theory and community detection [18]. We also employ a recently introduced analytical tool, ASTRICS, an algorithm and inter-cluster similarity measure based on triangulation of data points and α-shapes [19], and compare the results with those obtained with the original STORMGraph version. Finally, by applying a photon threshold filtering procedure we show that it is possible to reveal the mesoscale organization in STORM samples.

## 2. Results

### 2.1. Correlative Confocal and STED Microscopy 

**Figure 1:**
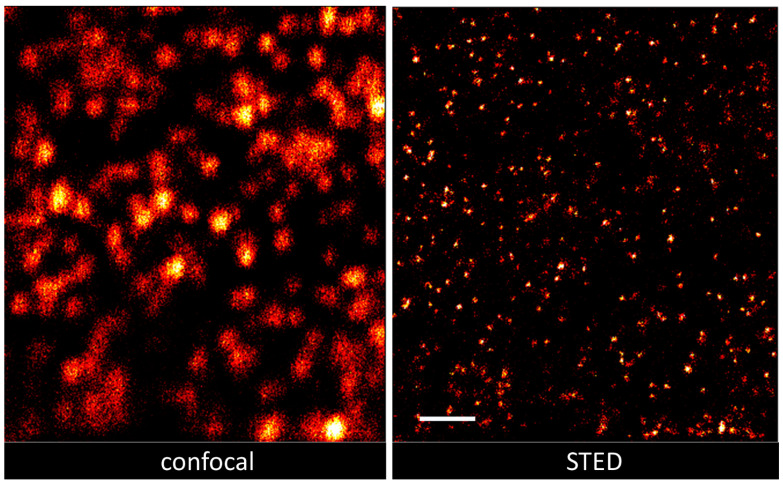
Confocal and STED images of the same ROI showing cell-surface nAChRs labeled with Alexa Fluor 647-mAb. Scale bar corresponds to 1 μm.

CHO-K1/A5 cells, a clonal cell line robustly expressing adult muscle-type nAChR [20] were used throughout. In the case of the primary monoclonal antibody, this protein was directly tagged with Alexa Fluor 647. PFA-fixed cells labeled with Alexa Fluor 647-BTX or Alexa Fluor 647-mAb35 against the nAChR α-subunit were imaged at their coverslip-adhered (ventral) surface. Between 8 and 20 cells were recorded per condition. As shown in Figure 1, in both series of experiments cell-surface membranes exhibited a fine punctiform distribution as well as spots of slightly larger nanometric dimensions. 

### 2.2. STORM Imaging

**Figure 2:**
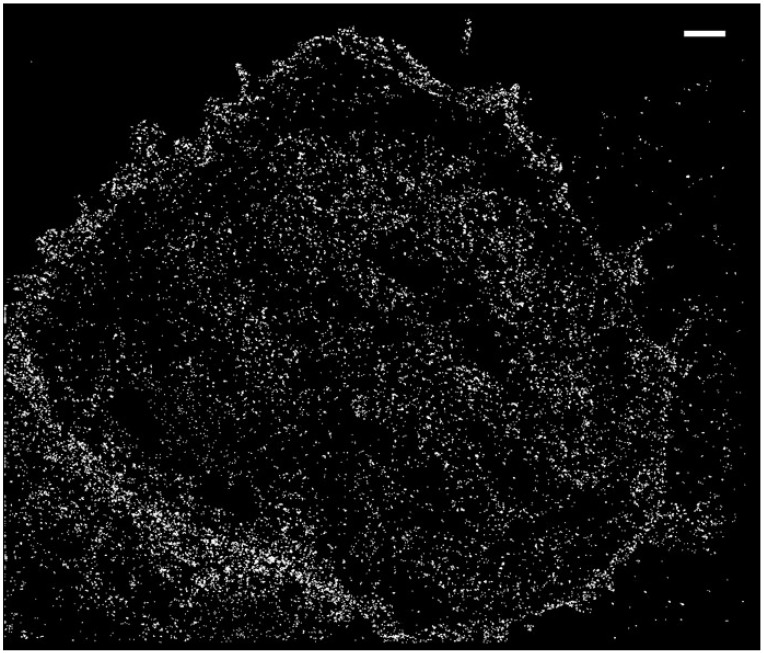
Representative raw STORM image showing reconstructed BTX-labeled nAChR localizations recorded from the entire coverslip-adhered ventral surfaces of CHO-K1/A5 cells. The ROI is 20 × 20 μm. The bar corresponds to 1 μm.

Figure 2 shows reconstructed nAChR puncta imaged with SMLM (STORM mode). Superresolution images of the entire flat ventral surfaces of the cells were acquired, containing tens of thousands of validated localizations.

### 2.3. STORMGraph Analysis of nAChR Nanoclusters

**Figure 3:**
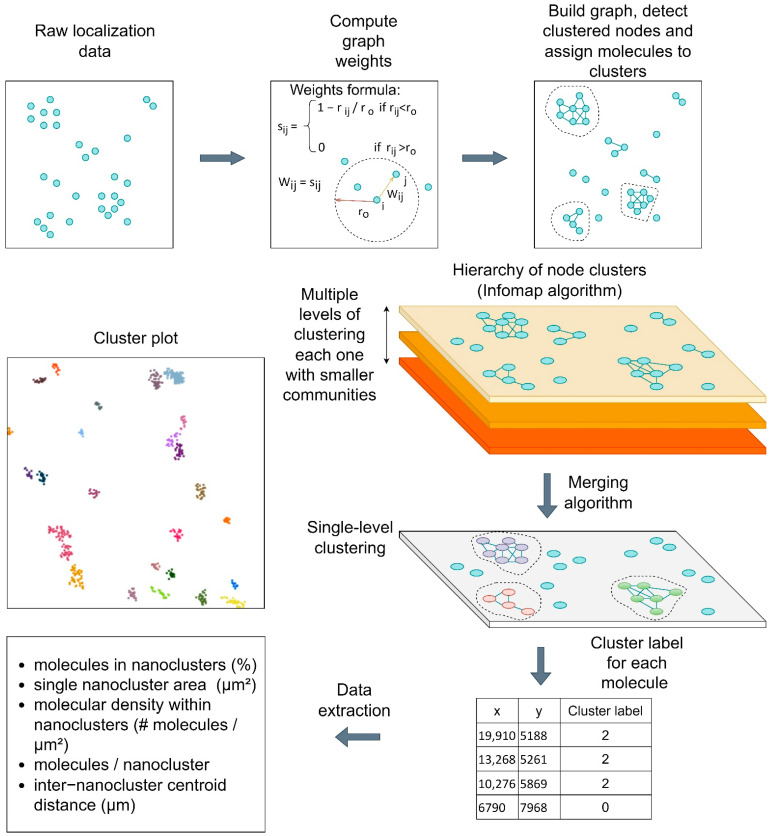
Schematic flow diagram of the STORMGraph analysis. STORMGraph first determines the ROI-specific length scale (r_o_) using a heuristic method or kNN distances. The graph is constructed using the weights formula in the central upper panel. Non-clustered nodes are subsequently removed, and the graph is regenerated with a new r_o_. A hierarchy of node clusters is found using the multi-level community detection algorithm followed by a merging algorithm that combines concepts of both symmetric kNN graphs and mutual kNN graphs. STORMGraph only merges clusters if they remain connected after the random removal or displacement of any node. The localization data with the cluster labels allows the extraction of data such as cluster area, localizations per cluster, and other metrics [18].

We next applied STORMGraph [18] for the identification and analysis of particle nanoclusters. This analytical approach differentiates these supramolecular aggregates from single nAChR particles at the cell surface. The method applies graph theory to detect particle cluster nodes and assign or discard molecules (particles) to clusters, as schematically depicted in Figure 3.

**Figure 4:**
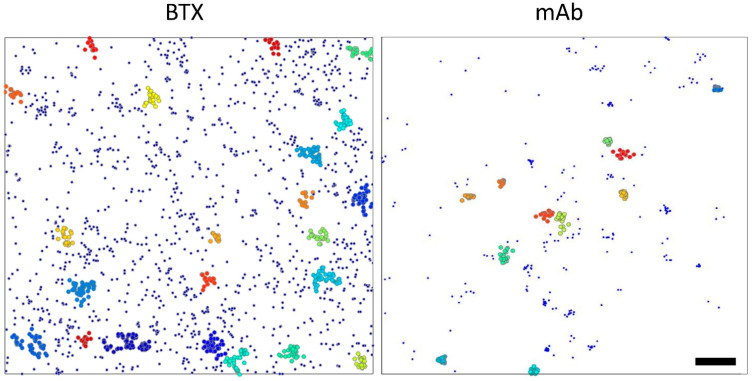
STORMGraph depiction of clustered and non-clustered (i.e., isolated single-molecule localizations) nAChR particles. The plots show the total single-molecule localizations of nAChR particles in a 10 × 10 μm ROI (**left** picture is the ROI number 3 of Figure S1). Non-clustered, isolated particles are shown in dark blue. nAChR molecules classified as clustered by STORMGraph are depicted as slightly larger colored spots, each color identifying an individual nanocluster. Individual spot sizes are not drawn to scale (size is irrelevant for the analysis). The cluster with the largest number of molecules in the example shown on the **left** (BTX-labeled sample) includes approximately 4% of the validated localizations in the ROI. In the case of the mAb-tagged sample (illustration on the **right**), the “macro” nanocluster outliers, i.e., the macro-nanoclusters account for up to 5%. The bar corresponds to 1 μm.

Using this approach nanoclusters were observed and quantitated under both experimental conditions. As shown in Figure 4, one of the outputs of STORMGraph provides immediate visual rendering of isolated single-particle localizations and distinct, individually identified nanoclusters, respectively. 

**Figure 5:**
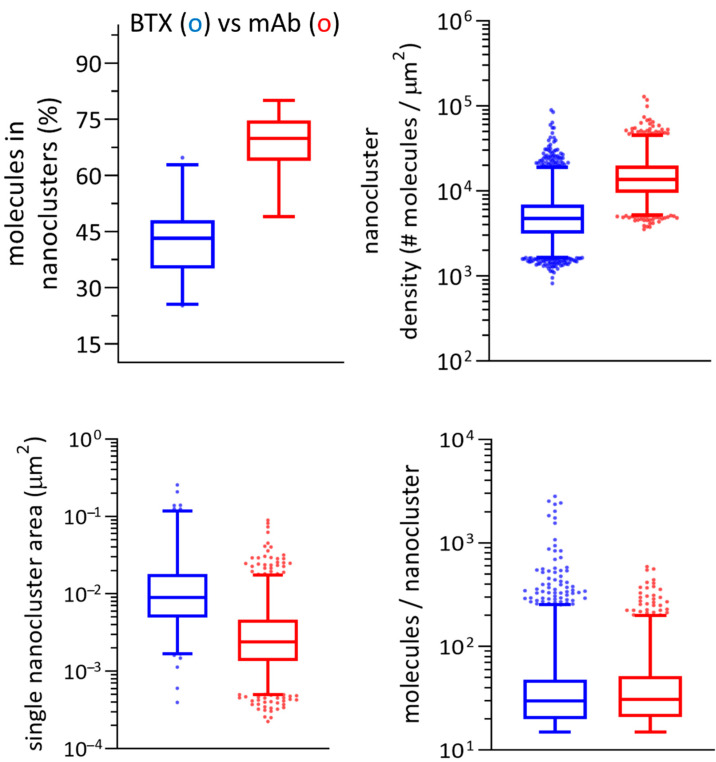
Main parameters derived from the STORMGraph analysis of the STORM experimental data. The whiskers represent the interquartile range and the median. The extremes of the intervals indicate the 2.5–97.5 percentile. The dots are outliers. All the plots (except for number of molecules in nanoclusters) are shown in log scale.

Examples of nanocluster parameters obtained through STORMGraph analysis of the two experimental super-resolution imaging conditions are shown in Figure 5. The number of molecules assigned to a cluster and the relative nanocluster density was higher for mAb-labeled samples (*p* < 0.0001). Statistically significant differences were also observed for the individual nanocluster area, and the inter-nanocluster centroid distance (See Figure S3), higher for BTX-labeled samples (*p* < 0.0001). The number of molecules per unit area within a single nanocluster (i.e., intra-cluster molecular density) was higher in BTX-labeled samples (*p* < 0.05). 

To learn whether the distribution of single-molecule localization varied across the surface of the plasmalemma, we selected ROIs from the peripheral and central regions of the cell (Figure S1). The periphery of the cells exhibited a higher density than the central region in mAb (<0.01) and BTX-labeled samples (<0.0001), but no differences were observed in the number of clustered molecules between these two regions for a given fluorescent label. From this type of analysis, we can conclude that the nanoclusters in the peripheral region of the cell are more tightly packed, in smaller areas, than those located in the central area of the cell. This is in agreement with the analysis of inter-nanocluster centroid distances (Figure S3). The statistics indicate that the centroids of the nanoclusters are separated by larger distances in the central region, both for BTX and mAb (*p* < 0.0001). Numerical results are listed in Table S1 and shown in Figures S2 and S4.

### 2.4. STORMGraph + ASTRICS Analysis of nAChR Nanoclusters 

**Figure 6:**
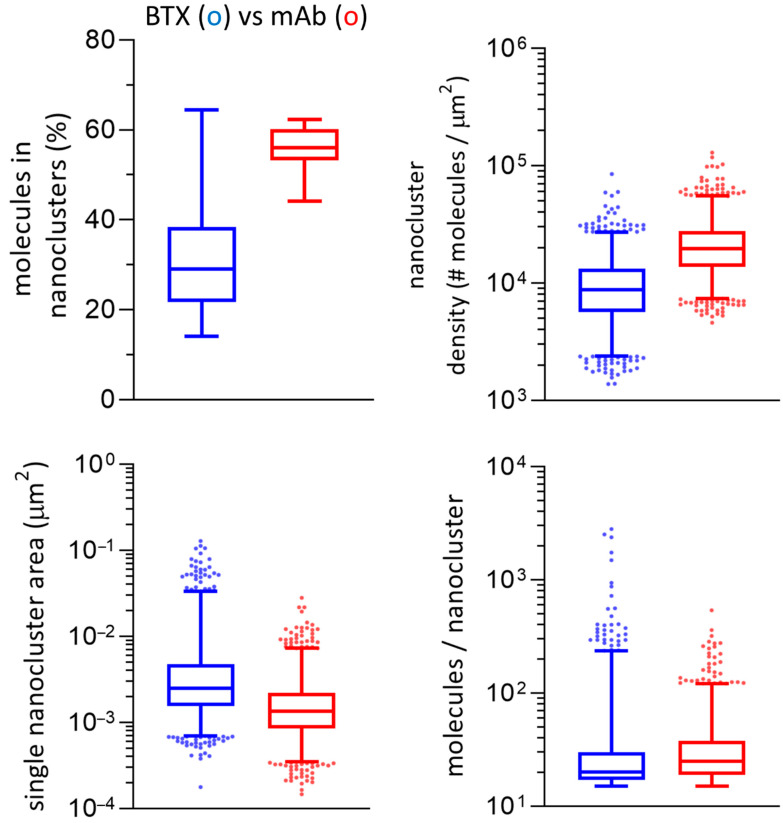
Main parameters derived from the STORMGraph + ASTRICS analysis of the STORM experimental data. The whiskers represent the interquartile range and the median. The extremes of the intervals indicate the 2.5–97.5 percentile. The dots are outliers. All the plots (except for molecules in nanoclusters) are shown in log scale. Metrics derived from these analyses are listed in Table 1 and in the Discussion section.

The addition of ASTRICS to the STORMGraph analysis (STORMGraph+ASTRICS) applies the similarity measure implemented in ASTRICS [19] to the lower levels of the cluster hierarchy generated by STORMGraph. The combined algorithms were used to identify nanoclusters, and multiple variables quantitated under both experimental conditions using custom-written software. mAb-tagged samples exhibited higher densities, a larger proportion of clustered molecules, and clusters with a higher number of molecules than BTX-labeled samples (*p* < 0.0001). BTX-labeled samples showed larger nanocluster areas and larger inter-nanocluster centroid distances than mAb-tagged samples (*p* < 0.0001). Results of these analyses are shown in Figure 6.

**Figure 7:**
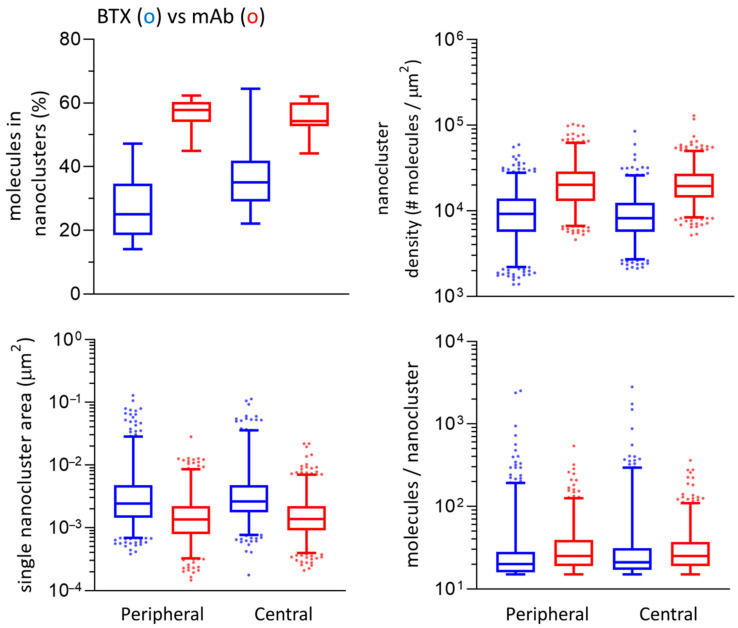
Main parameters derived from STORMGraph + ASTRICS of the STORM experimental data in the peripheral and central regions of the cell. The whiskers represent the interquartile range and the median. The extremes of the intervals indicate the 2.5–97.5 percentile. The dots are outliers. All plots (except for the one showing molecules in nanoclusters) are in log scale. The numerical values for this Figure are listed in Table 2.

Central regions from BTX-labeled samples showed larger centroid distances (*p* < 0.05), larger areas (*p* < 0.01), and lower densities (*p* < 0.001) than peripheral regions from the same labeled samples. Peripheral regions from mAb-labeled samples showed statistically significant higher densities than central regions (*p* < 0.0001). The latter exhibited larger centroid distances than peripheral regions from mAb (*p* < 0.001). No statistical differences were found in the number of clustered molecules, the number of molecules per nanocluster, and areas when comparing the two regions from mAb-labeled samples. When we further compared parameters from the same regions of mAb vs. BTX-labeled samples, we found that central regions from mAb-labeled samples showed higher densities (*p* < 0.05), a higher percentage of clustered localizations (*p* < 0.001), and a larger number of molecules per nanocluster (*p* < 0.0001) than BTX-labeled samples. Central and peripheral regions from BTX-labeled samples showed larger centroid distances and larger areas than mAb-labeled samples (*p* < 0.001). Peripheral regions from mAb-labeled samples had higher densities, more clustered molecules, and a larger number of molecules per nanocluster than BTX-labeled samples (*p* < 0.001, *p* < 0.0001, *p* < 0.0001, respectively). Results of this analysis are shown in Figure 7 and listed in Table 2.

### 2.5. Comparison between STED and STORM Parameters

Because of the physical principles on which the two nanoscopy approaches rely (see e.g., [10]), the results obtained with STED and STORM complement each other and provide information on different scales; however, only a few metrics can be directly matched, since one of them (STORM) looks at single molecules and macromolecular aggregates whereas STED currently resolves the latter [11]. Thus, the number of single-molecule localizations, the percentage of clustered localizations, the number of localizations per cluster and the density of the nanoclusters were quantified in STORM samples only. The distances between nanocluster and spot centroids as well as their size could be measured in the two types of nanoscopies. The areas of the spots imaged with STED were on average larger than those of the STORM nanoclusters (*p* < 0.0001), and the inter-centroid distances measured in STED images were roughly twice the median distance of the nanoclusters identified with STORM (*p* < 0.0001) (Table 3), suggesting that the two techniques accentuate different scales of the samples. 

Information about the shapes of the spots and nanoclusters was also obtained. Since most of them exhibited an ellipsoid shape under both types of nanoscopy, we implemented a method (detailed in Supplementary Material) to calculate the eccentricity of the ellipsoid and the length of its major axis. STED spots had a greater eccentricity than STORM nanoclusters (*p* < 0.0001). In STED BTX-tagged samples, inter-spot distances were approximately 8.6 times larger than the corresponding BTX-labeled inter-nanocluster distances in STORM (*p* < 0.0001), and in mAb-tagged samples, inter-spot distances were 10.8 times longer than inter-nanocluster distances imaged in STORM (*p* < 0.0001).

Using the distribution function G(d) (see Material and Methods for a detailed explanation), in the case of STORM the scale of the clustering was higher in mAb than in BTX samples (*p* < 0.0001), whereas in the case of STED, no statistical differences were found between mAb and BTX-labeled samples. As expected, STED scales were approximately 6 times higher in BTX-labeled, STED-imaged samples than in BTX/STORM samples (*p* < 0.0001). In the case of mAb STED samples, they were 3 times higher than in mAb/STORM (*p* < 0.0001).

### 2.6. Nanocluster Distribution in the Peripheral and Central Regions of the Coverglass-Adhered Plasmalemma

When we applied the ASTRICS analysis to the STORM data, we found that the metrics derived from the combined STORMGraph + ASTRICS approach rendered smaller nanocluster sizes, similar to those obtained in previous work from our laboratory [14,15]. In addition, some large elongated nanoclusters depicted by STORMGraph where split by ASTRICS into several smaller clusters with a more circular profile. Due to the low dimensionality of the localizations, ASTRICS bypasses the CS step, i.e., reduction in dimensionality, and moves directly to the ASTRI step, which uses α-shapes. An α-shape is a representation of the shape of a set of points. The degree of detail of the α-shape is determined by α [21]: if α decreases, more detail can be observed in the set [22]. Thus, the last step takes seed clusters and, for every pair of clusters, calculates its α-shape such that α is a minimum: α-shape encloses all data points from one of the paired clusters. The way α is selected increases the probability that well-separated clusters have zero similarity. If similarity is zero for a given pair of clusters, these are not merged. Otherwise, they are merged, and the algorithm continues until no more similarities are found between clusters. A more detailed explanation is given in Supplementary Material. The metrics derived from this series of analysis are listed in Table 2 and Table 3.

As shown in Table 3, differences are observed in the distribution of nAChR molecules between the two labeling conditions. The most striking difference is the higher percentage (~50%) of nAChRs occurring in clusters, and the higher relative density of receptors in mAb-tagged nanoclusters relative to BTX-labeled samples, a consequence of the antibody-induced crosslinking. The cell-surface coverage by nanoclusters (nanocluster density) is also much lower in BTX samples, which exhibit larger nanocluster sizes. However, the number of molecules within a nanocluster, and the distribution of receptors between central and peripheral regions, is essentially the same independently of the labeling conditions. 

### 2.7. ASTRICS for Low Dimensional Data and Its Combination with STORMGraph

**Figure 8:**
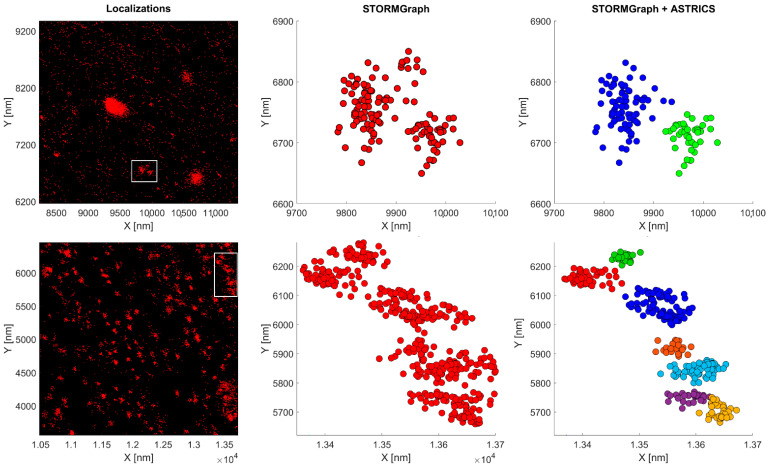
Comparison of clusters detected with STORMGraph alone with those obtained using the STORMGraph + ASTRICS combination on BTX samples (upper row) and mAb samples (lower row). It is apparent that (i) ASTRICS analysis results in smaller nanoclusters and (ii) some localizations are excluded from the clusters upon application of ASTRICS.

When visualizing STORMGraph depictions (Figure 8) we noticed that some nanoclusters exhibited an elongated appearance, while others showed single-particle localizations that appeared to lie far away from the densest part of the clusters. STORMGraph creates an additional level on top of the multi-level Infomap cluster hierarchy, merging those clusters that are sufficiently interconnected. To test whether the STORMGraph-generated clusters were artifactually biased toward larger clusters, ASTRICS was applied at the lowest hierarchical levels of STORMGraph to merge clusters. The ASTRICS approach resulted in the identification of clusters having roughly half the area of those detected by STORMGraph alone, and cluster densities were twice as high as those resulting from STORMGraph analysis alone.

## 3. Discussion

### 3.1. The Idea behind Complementary Correlative Microscopy Approaches

Combining different modalities of optical microscopies provides the means to compare, complement, and expand the information yielded by the individual approaches, and ultimately produces a much richer depiction of the object under study. Thus confocal microscopy together with stochastic STORM [23,24], super-resolution optical fluctuation imaging (SOFI) [25] combined with confocal microscopy [26], STED complemented with RESOLFT techniques [27], or various SMLM modalities blended with STED microscopy [28,29,30] have been applied to this end. In the last example the successful complementary approach was aimed at minimizing the spectral crosstalk of the fluorophores employed in STED, sptPALM, and uPAINT, respectively. In the present work the goal was to compare the cell-surface topography of the nAChR rendered by two receptor-specific biological probes with inherently different abilities to label the nicotinic receptor protein. The small peptide α-bungarotoxin, a quasi-irreversible competitive antagonist of the nAChR, is a monovalent ligand that labels the α subunits of the nAChR in a 2:1 stoichiometry relative to the receptor monomer. In contrast, the monoclonal antibody mAb35 is a multivalent, *crosslinking* ligand. Importantly, both receptor-specific ligands were tagged with the *same* red-absorbing cyanine dye, Alexa Fluor 647, which in the case of mAb35 was directly attached to the primary antibody. Furthermore, imaging was performed using two nanoscopy modalities, targeted STED super-resolution imaging and single-molecule reconstruction STORM nanoscopy, that rely on different physical principles and photochemical properties of the dyes. This combination provided a consistent complementary depiction of the distribution of the nAChR protein at the cell surface.

### 3.2. The Alexa Fluor 647 Fluorophore and Imaging Conditions

The field of nanoscopy grew hand-in-hand with the development of appropriate fluorescent probes, both for biological and materials science applications. The original implementation of STORM, for instance, required multiple lasers and the appropriate combination of rather limited pairs of fluorescent dyes (e.g., Alexa Fluor 405 for activation/Alexa Fluor 647 for imaging), an approach implemented up until a few years ago. In the present work, the choice of the cyanine dye Alexa Fluor 647 was initially based on its top overall performance in SMLM: high extinction coefficient, photostability, and relatively high photon yield per switching event, low on-off duty cycle, and good survival fraction [31]. In addition, the probe has a low spectral heterogeneity, i.e., it exhibits a spectrally homogeneous (692 ± 3.3 nm) single-molecule fluorescence emission [32], which is conserved upon conjugation to an antibody [33]. As expected, Alexa Fluor 647 performed very well in STORM using a thiol-containing glucose oxidase/catalase (GLOX) imaging oxygen scavenger system with a relatively low concentration of glucose (<2%). Contrary to expectations, its subsequent application under identical labeling and similar imaging conditions and using the same oxygen-depleted buffer in the STED experiments showed that the fluorescent probe conjugated either to the small peptide (~8000 MW) α-bungarotoxin or to an antibody macromolecule performed remarkably well in this targeted-microscopy modality. Oxygen depletion is expected to prolong the triplet state lifetime and decrease photon counts using the higher laser intensities required for STED because photobleaching occurs predominantly from this state [34]. In the present work, laser power was kept to a minimum for imaging with reasonable scanning times to allow for recovery of the bright “on” state. To maintain conditions as close as possible to those employed in STORM, imaging in STED was undertaken in the same buffer as used in SMLM.

### 3.3. STORM Nanocluster Metrics

Aggregates of membrane proteins of nanoscopic dimensions at the cell surface are of great interest in cell biology because they represent a supramolecular organization of physiological importance in signal transduction [35,36]. Pioneer fluorescence energy transfer in the homo-FRET modality provided early evidence of the occurrence of these platforms in the mammalian cell CHO-K1 [37,38], the parental cell of CHO-K1/A5, the clonal cell line produced in our laboratory used in the present study, that robustly expresses adult muscle-type nicotinic receptors [20]. Near-field optical microscopy [35,39], STED [11,40], STED-FCS [41], and SMLM [6,42,43,44] have contributed to characterizing the presence of nanoclusters formed by a variety of membrane proteins at the plasmalemma of several cell types.

Various methods have been developed to identify and analyze membrane protein nanoclusters in fixed specimens. Some are based on Ripley’s K function [11,44], others on Bayesian statistics [45,46,47], or deep learning strategies [48], including Density-Based Spatial Clustering of Applications with Noise (DBSCAN) [49]. Most approaches are based on a user-specified minimum number of points lying within a user-specified radius, often resulting in a biased analysis and specific settings that may also vary between sub-regions of the same ROI. 

Mapping the topographical distribution of nAChR molecules obtained via a direct method such as STED can be reduced to finding a function *n(r)* describing the number of molecules at coordinate *r* ± Δ*r*/2, Δ*r* being the spatial interval in which molecules exhibit the on-fluorescence state. Δ*r* is effectively the spatial resolution given by the full-width at half-maximum of the effective point spread function (PSF) *h(r)* of the imaging process [50]. 

Here, we initially analyzed the STORM images using STORMGraph, an approach based on graph theory (see, e.g., PhenoGraph [51]) and graph merging [52,53] with community detection algorithms [54,55] such as Infomap [56] or the Louvain method [57]). STORMGraph determines thresholds adaptively, circumventing user-defined parameters and thus allowing batch analysis over heterogeneous samples using identical settings to avoid bias [18].

### 3.4. ASTRICS for Low Dimensional Data and Its Combination with STORMGraph

Some clustering algorithms such as ClusterViSu [58] and SR-Tesseler [59] resort to Voronoï diagrams to quantify clusters in SMLM. The STORMGraph + ASTRICS approach uses the Delaunay triangulation, a dual form of Voronoï tessellation. ASTRICS triangulates the α-shape utilizing a subset of the Delaunay method [19]. Owing to the sparseness of the similarity matrix returned by ASTRICS, the STORMGraph + ASTRICS combined analysis was able to detect smaller clusters than STORMGraph alone in both BTX- and mAb-labeled samples (Figure 8 and Figure S12). 

### 3.5. STORM Localizations Filtered by Photons Emitted Revealed the Occurrence of a Mesoscale Distribution Similar to That Directly Apparent in STED Samples

Since STED particles (“spots”) are local maxima directly observed in the micrographs, it is possible that the particles detected by ThunderSTORM in STED data correspond to the brightest localizations of the STORM data. In another attempt to establish correlations between STORM and STED datasets, we selected 10 μm × 10 μm ROIs from BTX and mAb STORM samples and filtered out particles according to the photons emitted by each localization. Those localizations that did not pass a given photon threshold were removed. We next obtained the inter-particle distances using the Delaunay triangulation method. The inter-particle distances as a function of photon threshold are shown in Figure S15. The thresholds that resulted in the closest inter-particle distances from STED were 18,000 and 16,500 for mAb and BTX, respectively, and the resulting localizations are shown in Figure S16. We then ran STORMGraph on the filtered STORM localizations. As shown in Table 4, the photon threshold filtering analysis revealed that despite the different sizes of the objects imaged by the two labeling techniques, the STORM nanocluster centroids in both BTX and mAb samples are separated at scales similar to those separating spots in STED (2–4 μm, Table 2).

### 3.6. Complementarity of Nanoscopies, Biosensors, and Biological Implications of the Findings 

Sil and coworkers [60] define the nano-scale organization of the CD44 as “being built of individual molecules brought together within ~10 nm distances” and mesoscale as “domains ~100 nm–1000 nm in scale”. Although these authors refer to a completely unrelated membrane protein, they concur with our results [14,15] in that the nano-scale organization of the molecules dictates, to a large extent, their mesoscale organization and dynamics. STORM provided a detailed quantitative description of nanocluster size, shape, distribution, and occupancy (number of molecules per cluster, density). STED delivered a complementary picture, providing a direct map of the mesoscale nAChR distribution, with inter-spot centroid distances ~10-times larger than those of the STORM approach. We introduced a novel approach, photon threshold filtering analysis, that brought out the mesoscale receptor organization in STORM samples. The combination of STORM and STED nanoscopies and novel image analytical techniques thus provides not only a fuller picture than that of each approach in isolation but also reconciles the nano- and mesoscale information rendered by the two methodologies. Differences between samples remain because of the biosensors used: the monovalent BTX vs. the polyvalent crosslinking antibodies. The former is most appropriate to characterize the nanocluster in fine detail, and the second rendered the mesoscopic organization of the clusters at a larger scale, with possible implications in the pathology of the peripheral cholinergic synapse [61,62]. Though nAChR nanoclusters may no longer occur as such in the tightly packed, adult neuromuscular junction, they are of importance during the early ontogenetic neurodevelopmental stages as the initial form of association of individual receptors that subsequently coalesce into patches ([63,64,65], reviewed in [66]) that fuse to produce the peripheral cholinergic synapse. 

## 4. Material and Methods

### 4.1. Materials

Mouse monoclonal antibody clone mAb35 (purified immunoglobulin, product No. M-217) against the extracellular moiety of the nAChR α_1_-subunit and Alexa Fluor 647 (product no. W32466) were purchased from Thermo Fisher, Germany. Catalase from *Aspergillus niger* (product no. C3515), glucose oxidase type VII (product no. G2133), and β-mercaptoethanol (product no. 63689) were obtained from Sigma Chem. Co. (St. Louis, MO, USA). 

### 4.2. Cell Culture

CHO-K1/A5 cells, a clonal cell line robustly expressing adult muscle-type nicotinic acetylcholine receptor (nAChR) [20] were grown in Ham’s F12 medium supplemented with 10% fetal bovine serum for 2–3 days at 37 °C before experiments. Cells are used for a maximum of 20 passages. No ethical approval was required for the use of the biological material.

### 4.3. Cell-Surface Fluorescence Staining of nAChRs

CHO-K1/A5 cells grown on 18 mm diameter no. 1.5 glass coverslips (WRL) in Ham’s F12 medium at 37 °C were washed thrice with M1 medium and fixed with 2.4% PFA for 10 min. After 3× rinsing with 1× PBS, the cells were quenched with 1× PBS containing 1% BSA with several changes for 3 min. Cells were then stained with Alexa Fluor 647-BTX at a final concentration of 1 μM for 10–30 min at 20 °C and finally washed thrice with a cold M1 medium containing 1% PBS. Another set of PFA-fixed cells was incubated with mAb35 monoclonal antibody coupled with Alexa Fluor 647. Coverslips with the adhered cells were subsequently mounted on glass slides and examined within 1–2 h. 

### 4.4. Single-Molecule Stochastic Optical Reconstruction Microscopy (STORM) Single-Molecule Localization Microscopy (SMLM)

Samples were imaged with a standard, custom-built STORM microscope, using β-mercaptoethanol-based blinking buffer, as described in ref. [67].

### 4.5. STED Nanoscopy Imaging

Imaging was performed using an add-on Stedycon STED nanoscope from the firm Abberior (Göttingen, Germany). Some experiments were performed using the Expert STED nanoscope from Abberior. 

### 4.6. Superresolution Data Analysis

#### 4.6.1. Sub-Diffraction Coordinates of STED Superresolution Images in Fixed Specimens Stained with Alexa Fluor 647-BTX or Alexa Fluor 647-mAb

Each series of STED images obtained from a given experiment was first pre-processed using ImageJ to provide optimal contrast and subtract background noise. Images were then analyzed with ThunderSTORM [68]. The “Difference of averaging filters” filter was set with a first kernel size of 3 [px] and a second kernel size of 5 [px]. The localization “Centroid of Connected Components” method was used, with the “std (Wave.F1)” peak intensity threshold. The “PSF: Integrated Gaussian” sub-pixel localization fitting procedure with a “weighted least-squares” routine was applied. 

#### 4.6.2. Single-Molecule STORM Localization in Specimens Stained with Alexa Fluor 647-BTX or Alexa Fluor 647-mAb

Frames were convolved with a Gaussian Kernel with unit height and width set as the expected PSF, lowered to have a zero integral [69]. This procedure reduces high-frequency noise and low-frequency background variations. A threshold was applied to the images, and the peaks were identified with the local maxima. A squared region of 5–7 pixels centered on each peak was passed through a non-linear least-squares fitting algorithm. To determine the positions of the fluorescent molecules, two fits were performed: first, the data were fit to a continuous ellipsoidal Gaussian. The center position, amplitude, and width were determined in the second fit step, where the data were fit to a rotationally symmetric Gaussian function. Finally, the total number of counts collected in the peak was calculated to obtain the number of photons detected. Next, a set of filtering criteria was applied to remove peaks corresponding to multiple activated fluorophores, which are very close to each other and appear as a single peak. Finally, peaks in time-contiguous frames that appear with a relative displacement of one pixel or less are grouped together [70]. 

When present, drift was corrected using the method of Mlodzianoski, which is integrated into the software package ThunderSTORM [68] with 5 bins and a magnification of 5.0×.

#### 4.6.3. Nanocluster/STED Analysis Using STORMGraph

The STORM single-molecule coordinates were merged using a weighted arithmetic mean with the number of photons of each individual localization as weight [13]. The STORMGraph [18] automated quantitative clustering analysis of SMLM images was applied next; initial parameters were configured using the following values: K = 10, minimum cluster size = 15, and α = 0.05, the maximum probability of a completely randomly distributed localization being classified as clustered, with a value of 0.05. The parameter that avoids multiple blinking was disabled. STORMGraph first determines the ROI-specific length scale (*r*_o_) using a heuristic method or kNN distances. The graph was constructed using the formula shown in Figure S3. Then, non-clustered nodes were removed, and the graph regenerated with a new *r_o_*. A hierarchy of node clusters was found next using the multi-level community detection algorithm [54], followed by a merging algorithm that combines concepts of both symmetric kNN and mutual kNN graphs. STORMGraph only merges clusters if they remain connected after the random removal or displacement of any node. Finally, the localization data with the cluster labels allowed the extraction of metrics pertaining to the nAChR supramolecular array, i.e., the nanocluster, such as single nanocluster area, number of molecules per nanocluster, nanocluster centroids, as well as metrics related to the supra- distribution of the nanoclusters, such as the inter-centroid distances and other parameters related to the mesoscopic topography of the clusters in the cell membrane.

Though ThunderSTORM and STORMGraph are, as their name indicates, intended for STORM data, the key parameters (intensities, local maxima, cluster area, distance between centroids) are applicable to STED data. The output of the ThunderSTORM-processed STED data with the (x,y) localization coordinates was subjected to STORMGraph [18] analysis. Key parameters set as start values included MinCluSize, the pixel size, the expected number of nanoclusters, and the parameter K (the number of nearest neighbors required to perform the kNN analysis). The output of the STORMGraph analysis containing information on the area, centroids, etc., was then used as input to a script to calculate statistical parameters associated with the image data. Relative fluorescence intensities of clustered and non-clustered STORM localizations and STED spots are listed in Table S2 and Figure S5, and their probability density distributions shown in Figures S6–S10. 

#### 4.6.4. Nanocluster Analysis Using the Combination of STORMGraph + ASTRICS

The STORM single-molecule coordinates were merged, and STORMGraph [18] was applied, as mentioned above. We also combined STORMGraph with ASTRICS, an inter-cluster similarity algorithm based on α-shapes and triangulation [19]. To this end, we used the second level of the hierarchy of clusters generated by STORMGraph followed by a merging algorithm that uses ASTRICS as a similarity measure skipping the CS step, as described in Supplementary Material Figures S11–S13. Once ASTRICS establishes the similarity of each cluster pair, clusters are merged if and only if they have the maximum ASTRICS similarity over all clusters, and this value differs from zero. This process is repeated until no mergeable clusters are found. Finally, all those clusters that do not pass a minimum threshold number of 15 localizations are discarded. Metrics such as single nanocluster area, number of molecules per nanocluster, nanocluster centroids, and other parameters shown in Results were extracted from the remaining clusters.

#### 4.6.5. Cluster Shape Analysis Using an Elliptic Fitting Algorithm

Upon application of clustering algorithms, one can analyze the shape of the resulting clusters. One way to accomplish this is by using direct least-squares fitting on the clusters’ boundaries. A least square fitting specifically designed for ellipses was employed [71]. However, when the number of experimentally observed clusters is relatively low, as in STED experiments, axis lengths that disproportionally exceed cluster dimensions were observed using this method. To consider cluster geometry and not just cluster boundaries, we implemented a method that includes the farthest point of a given cluster, moves it to the origin, and rotates the cluster until both points are horizontally aligned. The short axis of the resulting ellipse is calculated next as the vertical distance between the maximum and minimum points on the y-axis. The implemented algorithm is described in Supplementary Material Figure S14.

#### 4.6.6. Inter-Particle Distance Using Delaunay Triangulation

**Figure 9:**
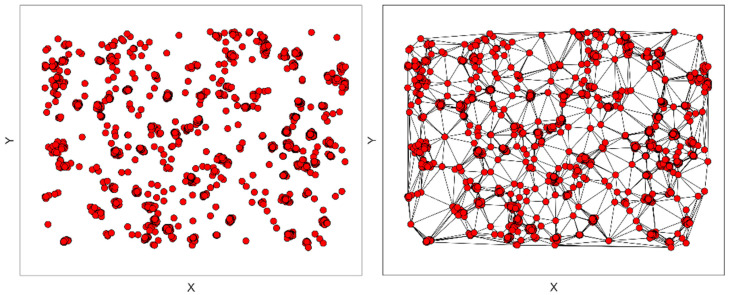
A set of localizations from STORM (**left**) and its Delaunay triangulation (**right**). Localizations are linked with neighboring ones, discarding connections at long distances.

The most straightforward way to establish distances between points in a dataset is, of course, to measure the distance between every point. However, this has the drawback that it includes distances so great as to make them biologically irrelevant. Another approach is to measure the Euclidean distance between a particle and its neighbors. To reduce the number of distances considered, we employed the Delaunay triangulation. The output was a graph whose weights are the distances between linked particles. Delaunay triangulation conforms to the dual graph mode of a Voronoi diagram, in which two particles are linked if they are close to each other in the Voronoi diagram partition (Figure 9). This methodology, previously used to analyze the distributions of the distances between localized fluorophores [72], was applied to both STED and STORM data.

#### 4.6.7. Nearest Particle Distance Analysis

Planar point pattern analysis was conducted to determine if the particle distribution in samples from both techniques constituted clusters and, if so, up to which distance. We used the PySAL [73] submodule pointstat that includes an “event-to-event” nearest neighbor distance function $G\left(d\right)=\overset{n}{\underset{i=1}{\sum }}I(d_{min}\left(p_{i}\right)<d)/n$ such that $d_{min}\left(p_{i}\right)$ is the nearest particle distance of the particle $p_{i}$ and I is the indicator function fulfilling the condition if $d_{min}\left(p_{i}\right)<d$ is true, I returns 1 [73]. Otherwise, it returns a value of 0. G(d) is the proportion of nearest particle distances that are less than d and is a cumulative distribution function.

We also used PySAL to simulate random patterns (*n* = 99) for each STED and STORM sample having the same size and density to extract a 95% confidence interval (CI) from Complete Spatial Randomness (CSR) patterns in order to determine the presence of clusters in the samples and, if so, the distances up to which the clustering extended. As shown in Figure S15, if G(d) is greater than the upper limit of the CI, there is clustering. The meaning of the G(d) function with the confidence intervals is shown in Figure S17, and two examples of STORM and STED are in Figure S18. Figures of G(d) functions in STORM, and STED samples are found in Supplementary File S1.

#### 4.6.8. Statistical Analyses

The one-sample Kolmogorov–Smirnov test was applied to assess whether the data were normally distributed or not. To compare two distributions, we used the Kolmogorov–Smirnov (KS) test for two samples. The experimental procedures (cell cultures, immunocytochemistry, STORM imaging) and the statistical analyses were performed by different individuals. The median ± 95% confidence interval is shown unless otherwise stated. Cluster parameters were computed with MATLAB v9.10.0 (R2021a) using, in the case of areas, its boundary function. Statistical Analyses were conducted with GraphPad Prism 8.

## Figures and Tables

**Table 1:** Comparison of parameters resulting from application of the STORMGraph + ASTRICS analysis to STORM data *.

Parameters	BTX	mAb
% Localizations in nanoclusters	29.12 (24.31–36.34)	56.04 (53.18–60.17)
Single nanocluster area (μm²)	0.0025 (0.0024–0.0026)	0.0013 (0.0013–0.0014)
Molecule density (number of molecules/μm² within individual nanocluster)	8726 (8422–9025)	19,661 (18,941–20,408)
Validated localizations per nanocluster	20 (20–21)	25 (24–26)
Inter-nanocluster centroid distance (μm)	2.25 (2.24–2.26)	1.58 (1.57–1.59)

* Data are expressed as median and lower 95% CI of the median/upper 95% CI of the median values in brackets.

**Table 2:** Metrics derived from STORMGraph + ASTRICS analysis of STORM images, subdivided into peripheral and central regions *.

	BTX		mAb	
Median	Peripheral Region	Central Region	Peripheral Region	Central Region
% Molecules in nanoclusters	24.99 (19.59–32.28)	35.11 (29.00–42.61)	57.80 (44.92–62.34)	54.32 (49.06–60.94)
Single nanocluster area (nm²)	2494 (2294–2658)	2665 (2531–2813)	1375 (1297–1519)	1337 (1264–1411)
Nanocluster density (number of molecules/nm²)	9.15 (8.65–9.81)	8.21 (7.80–8.67)	20.14 (18.97–21.23)	19.29 (18.61–20.25)
Molecules/nanocluster	20 (19–21)	21 (20–22)	25 (23–26)	25 (24–26)
Inter-nanocluster centroid distance (nm)	2248 (2233–2262)	2272 (2253–2291)	1478 (1467–1489)	1663 (1651–1673)

* Data are expressed as median and lower 95% CI of the median/upper 95% CI of the median values in brackets.

**Table 3:** Comparison of parameters obtained by applying STORMGraph + ASTRICS analysis to STED and STORMGraph to STED data *.

	STORM		STED	
Parameters	BTX	mAb	BTX	mAb
% Localizations in nanoclusters	29.12 (24.31–36.34)	56.04 (53.18–60.17)	-	-
Nanocluster/spot area (μm²)	0.0025 (0.0024–0.0026)	0.0013 (0.0013–0.0014)	0.004 (0.003–0.005)	0.001 (0.0006–0.0017)
Relative nanocluster density (number of molecules/μm²)	8726 (8422–9025)	19,661 (18,941–20,408)	-	-
Validated localizations per nanocluster	20 (20–21)	25 (24–26)	-	-
Inter-nanocluster/spot centroid distance (μm)	2.25 (2.24–2.26)	1.58 (1.57–1.59)	4.42 (4.36–4.47)	3.2 (3.06–3.32)
Nanocluster/spot eccentricity	0.72 (0.71–0.73)	0.69 (0.68–0.70)	0.93 (0.91–0.94)	0.89 (0.85–0.94)
Major axis length of nanocluster/spot (μm)	0.086 (0.084–0.088)	0.061 (0.060–0.063)	0.360 (0.332–0.399)	0.150 (0.107–0.267)
Inter-particle distances (nm)	42.42 (42.23–42.63)	14.23 (14.17–14.28)	366.5 (362.6–369.7)	153.9 (150.5–158.4)
Nearest particle distances (nm)	15.70 (15.50–15.80)	11.20 (11.10–11.20)	75.70 (70.40–80.70)	20.65 (19.50–22.70)
Maximum distance of considerable clustering (nm)	47.00 (41.60–51.00)	109.0 (72.20–208.0)	317.5 (300.0–357.0)	354.0 (311.0 566.0)

* The ThunderSTORM analysis applied to the STED data was used as STORMGraph input. Parameters k , m, and α (see Material and Methods) were different for each ThunderSTORM analysis.

**Table 4:** Metrics derived from STORMGraph analysis of BTX- and mAb-labeled nAChR STORM with photo-filtered data *.

	STORM Data after Filtering	
Parameters	BTX	mAb
Cluster area (μm²)	0.0053 (0.0032–0.0076)	0.0007 (0.0005–0.0016)
Cluster centroid distance (μm)	4.82 (4.72–4.90)	4.34 (4.29–4.40)
Cluster eccentricity	0.95 (0.91–0.96)	0.92 (0.88–0.95)
Major axis length of cluster (μm)	0.207 (0.173–0.255)	0.087 (0.076–0.104)
Maximum distance of considerable clustering (nm)	347.0 (284.0–408.0)	292.0 (190.0–503.0)

* The filtered STORM data were used as STORMGraph input. Parameters were, in the case of BTX, k=2, m=2, and α=0.05. In the case of mAb, k=5, m=2, and α=0.05.

## Data Availability

Raw data and software scripts are available upon request from the corresponding author.

## Supplementary Materials

The following supporting information can be downloaded at: https://www.mdpi.com/article/10.3390/ijms231810435/s1.
