# Supplementary material for: Mapping the Nicotinic Acetylcholine Receptor Nanocluster Topography at the Cell Membrane with STED and STORM Nanoscopies
Source: Int J Mol Sci. 2022 Sep 9;23(18):10435. doi: 10.3390/ijms231810435 (PMC9499342; doi:10.3390/ijms231810435)
Supplement: Supplementary file 1 [file ijms-23-10435-s001.zip › STED-STORM Suppl Mat.pdf]

Supplementary material

Distribution of nanoclusters in the peripheral and central regions of the ventral plasmalemma

In addition to the general analysis of single-molecule localizations at the plasmalemma, we analyzed the metrics of nAChR topography across the ventral cell surface by selecting ROIs in the periphery and in the central regions of the cell (Figure S1).

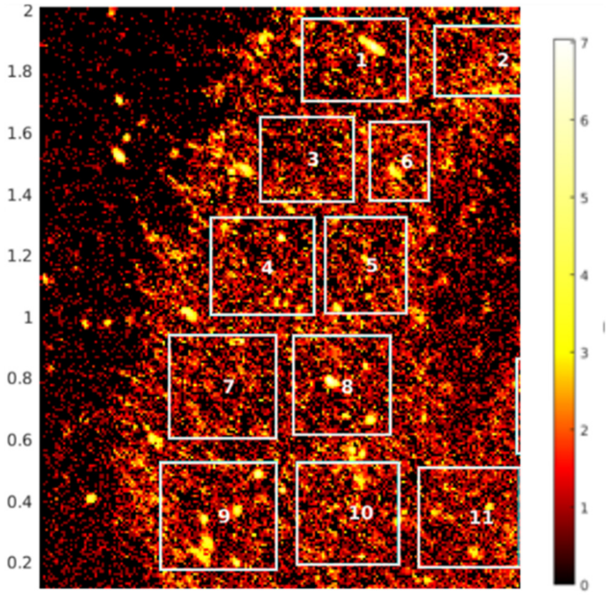

Figure S1: nAChR distribution in central and peripheral regions of a cell imaged with STORM. In the *CROP* option, STORMGraph analysis requires selection of individual ROIs; the program provides a manual ROI selection option. ROIs (white rectangles) were selected as shown and given a unique identifier (here, 1-11). Metrics of the results are given in Table S1 and Figure S2 below.

Table S1: Metrics derived from STORMGraph analysis of BTX- and mAb-labeled nAChR STORM data, subdivided into peripheral and central regions.*

	BTX		mAb	
Median	Peripheral Region	Central Region	Peripheral Region	Central Region
% molecules in nanoclusters	43.71 (36.63 - 47.91)	46.41 (43.19 - 49.65)	70.06 (64.13 - 76.30)	67.12 (57.20 - 76.56)
nanocluster area (µm²)	0.006 (0.005 - 0.007)	0.007 (0.006 - 0.008)	0.0023 (0.0021 - 0.0024)	0.0026 (0.0024 - 0.0028)
relative nanocluster density (# molecules / µm²)	5163 (4999 - 5347)	4483 (4263 - 4592)	14763 (14006 - 15520)	13058 (12474 - 13609)
molecules / nanocluster	29 (28 - 31)	30 (29 - 32)	31 (29 - 33)	32 (30 - 34)
inter-nanocluster centroid distance (µm)	2.3 (2.29 - 2.31)	2.4 (2.38 - 2.41)	1.52 (1.50 - 1.53)	1.65 (1.63 - 1.66)

* Data are expressed as median and lower 95% CI of the median / upper 95% CI of the median values in brackets.

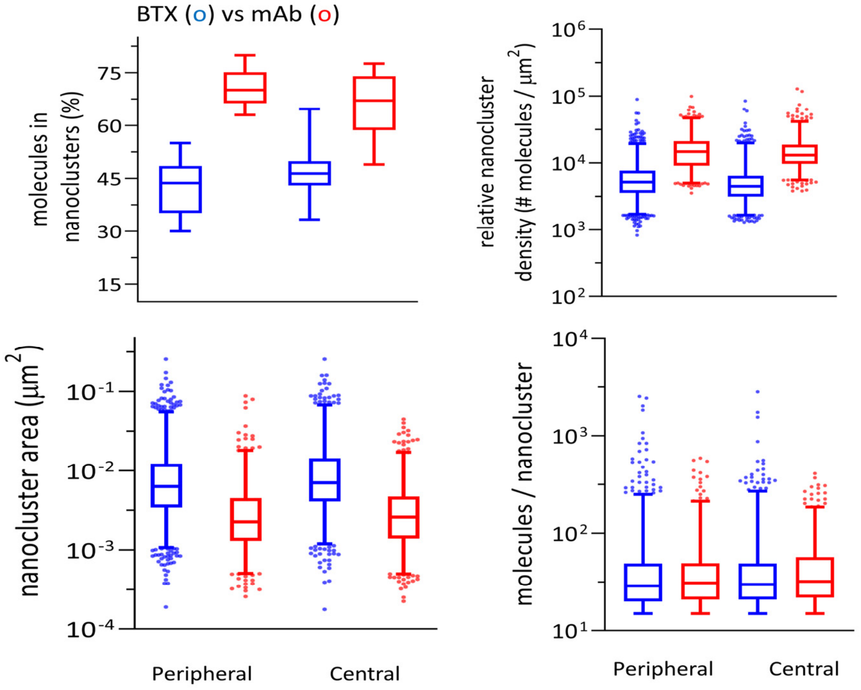

Figure S2: Main parameters derived from the STORMGraph analysis of the STORM experimental data. The whiskers represent the interquartile range and the median. The extremes of the line indicate the 2.5 – 97.5 percentiles. The dots are outliers. All plots (except for the one showing molecules in nanoclusters) are in log scale. The numerical values for this Figure are listed in Table S1.

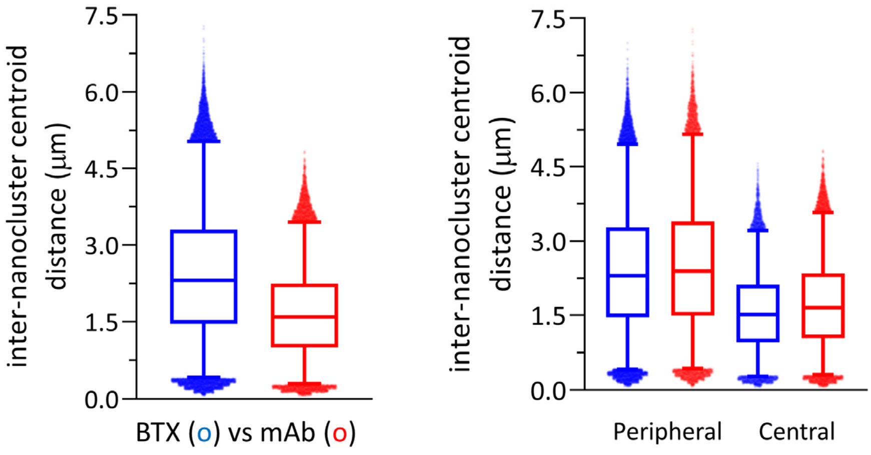

Figure S3: Mean distances between the centroids of neighboring nanoclusters from STORMGraph analysis. The whiskers represent the interquartile range and the median. The extremes of the line indicate the 2.5 – 97.5 percentile. The dots are outliers.

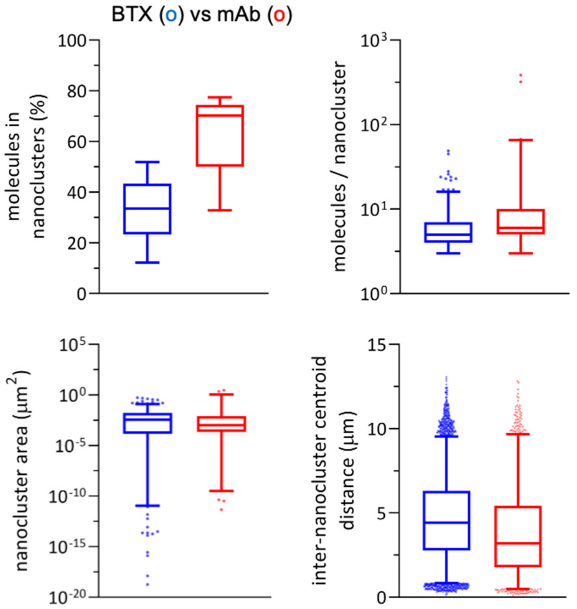

Figure S4: Parameters derived from the STORMGraph analysis of STED experimental data. The whiskers represent the interquartile range and the median. The extremes of the line indicate the 2.5 – 97.5 percentile. The dots are outliers. The molecules per nanocluster and the nanocluster area are in log-scale. The numerical values are listed in Table S1.

Intensity profile of the single particles and nanoclusters

Figure S5 shows the graphical representation of the intensities of clustered and non-clustered nAChR localizations obtained with STORM imaging. This figure and Table S2 show the higher fluorescence intensities of the single-molecule *isolated* particles (i.e. non-clustered localizations) in comparison to clustered localizations for the entire STORM data set (upper row) and for data sorted according to peripheral or central cell regions. Marked statistical differences were observed between BTX- and mAb-labeled samples. Non-clustered localizations have higher intensities than clustered ones in both BTX and mAb ($p < 0.0001$). Clustered localizations from BTX-labeled samples presented lower intensities than mAb localizations ($p < 0.001$). The same trend was observed for non-clustered localizations ($p < 0.0001$). In the case of mAb-labeled samples, differences were observed in the peripheral region ($p < 0.0001$), where non-clustered localizations showed lower fluorescence intensities ($p < 0.0001$) than the clustered ones. The oppossite was the case for central regions, where non-clustered localizations showed higher intensities than clustered ones ($p < 0.0001$). The intensities of the particles in STED and STORM data are shown for comparison in Table S2.

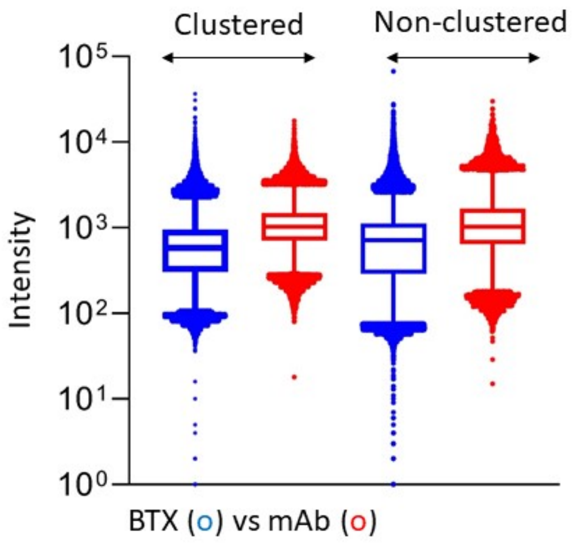

Figure S5: Intensity of BTX- and mAb-labeled samples in STORM images. Validated localizations were assigned as either clustered or non-clustered (free particles). The box plot (log scale) depicts the interquartile range, with the median represented in the middle. The extremes represent the 2.5th and the 97.5th percentile, respectively. The small dots are outliers. The statistical analysis of the distribution of the data showed a positive skewness with the intensity in all cases below 2,000 and 3,000 for BTX and mAb, respectively, and a right-tail with outliers with up to 67,400 photons.

Table S2: Relative fluorescence intensities of clustered and non-clustered STORM localizations ans STED spots*

Median	BTX		mAb	
	Clustered	Non-clustered	Clustered	Non-clustered
STORM, all ROIs	580 (577 - 583)	712 (709 - 715)	1025 (1020 - 1031)	1029 (1019 - 1040)
Peripheral	589 (585 - 593)	735 (731 - 740)	1076 (1068- 1084)	1044 (1030 - 1059)
Central	572 (568 - 577)	683 (677 - 689)	980 (972 - 986)	1016 (1002 - 1029)
STED**	368 (352 - 389)	330 (320 - 338)	1306 (1155 - 1459)	1246 (1145 - 1314)

*Median and 95% CI of the median are listed for BTX and mAb-labeled samples in the entire cell or discriminating according to intracellular region.

** In STED microscopy we cannot ascertain whether an imaged “spot” corresponds to a nanocluster, i.e., a clustered group of localizations as defined for STORM. Upon application of ThunderSTORM to STED images in the process of measuring spot intensities, these were divided into clustered and non-clustered localizations using STORMGraph.

As shown in Figure S6, significant differences were observed in the probability density distribution of intensities between BTX- and mAb-labeled samples: Non-clustered BTX-tagged nAChRs did not appear to follow a unimodal distribution. However, when Hartingan's Dip test was applied to assess whether the data was unimodal, the result was positive ($p < 0.0001$), indicating that the initial hypothesis was not correct. The small peaks in the BTX non-clustered data below ~125 photons represent sparse localizations from different regions of the cell, with an approximately uniform distribution over each ROI. The mAb-labeled clustered and non-clustered receptors followed also a simple unimodal distribution ($p < 0.0001$).

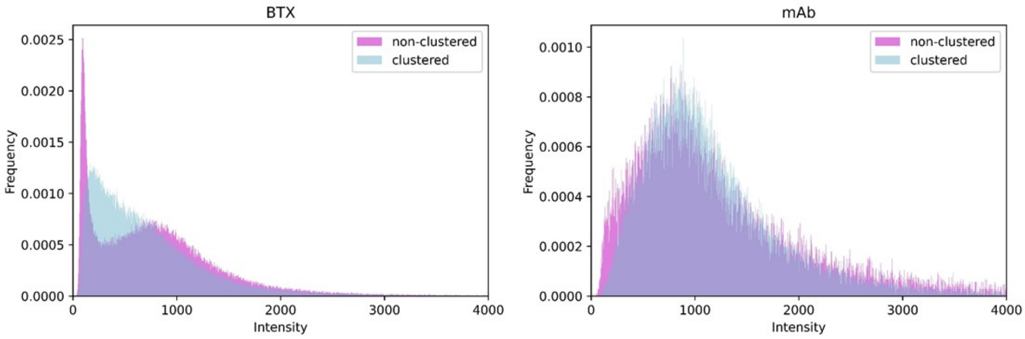

Figure S6: Probability density function (PDF) of the intensity of the localizations in BTX- and mAb-labeled samples. The cyan-colored areas represent the clustered localizations and the magenta-colored areas the non-clustered. A third color appears when the PDFs overlap. Note the small peak at approximately 125 photons for the case of BTX non-clustered samples, this peak is near the 12.5th percentile of the data. The Intensity axis only considers values under 4,000 to highlight the most remarkable differences. See Figure S5 to see the full range which includes the outliers.

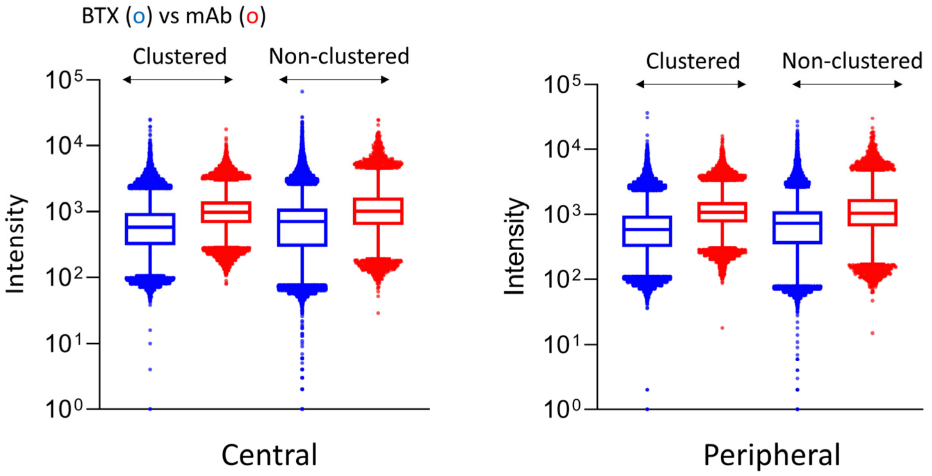

Figure S7: Intensities of the clustered and non-clustered STORM validated localizations upon sorting the ROIs according to the intracellular region. The box plot (log scale) indicates the interquartile range, with the median represented in the middle. The extremes represent the 2.5^{th} and the 97.5^{th} percentile. The small dots are outlier

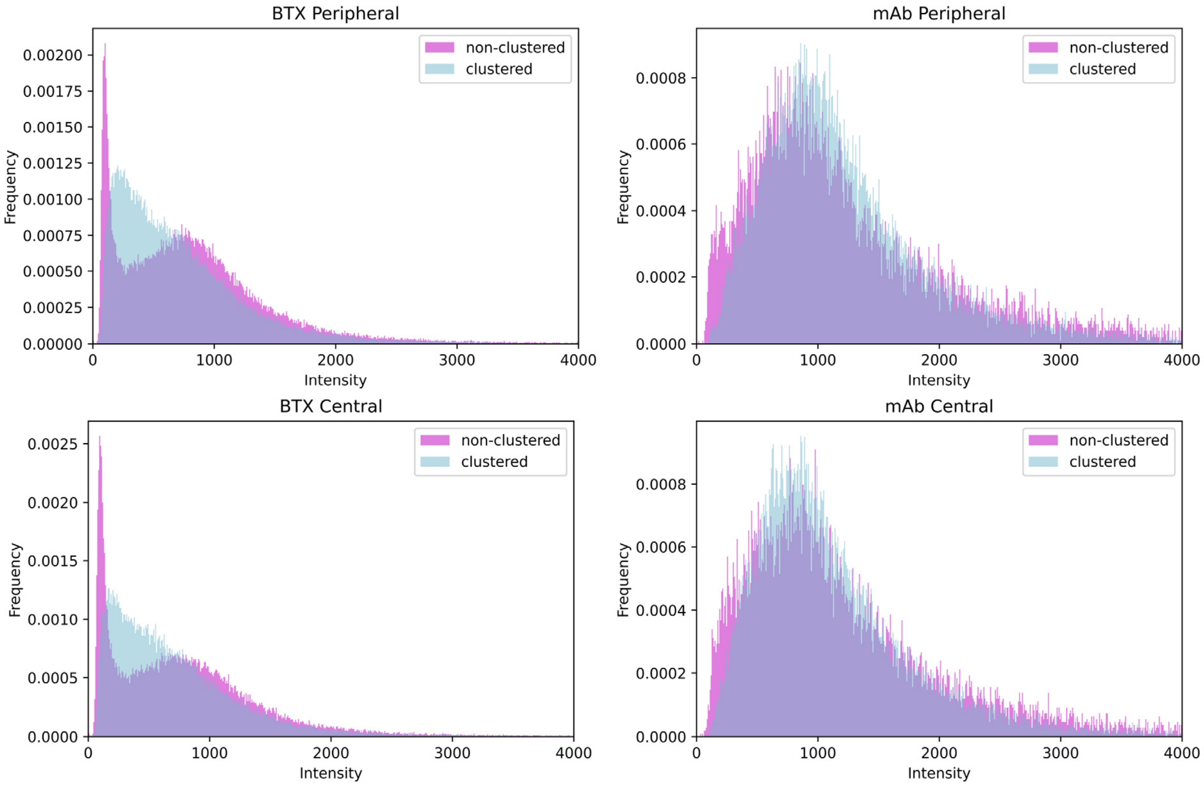

Figure S8: Probability density function (PDF) of the intensities for BTX- and mAb-labeled nAChR clustered and non-clustered localizations from peripheral and central regions of the cells. A third color appears when the PDFs overlap. Note the small peak mentioned in Figure S6 appearing in both peripheral and in central regions.

Application of ThunderSTORM to STED images rendered the intensity values of the nanocluster spots, as shown in Figures S9 and S10. Intensity distributions from mAb and BTX-labeled samples differ significantly: the intensities of mAb-labeled nAChR spots in STED are significantly higher ($p < 0.0001$, Figure S9) than those of BTX-labeled nAChRs. We also found statistical differences between the intensities of non-clustered and clustered nAChR spots from samples labelled with BTX and mAb ($p < 0.0001$, Figure S10). However, no statistical differences were apparent when comparing the intensities of clustered and non-clustered localizations from the same type of sample (Figure S9).

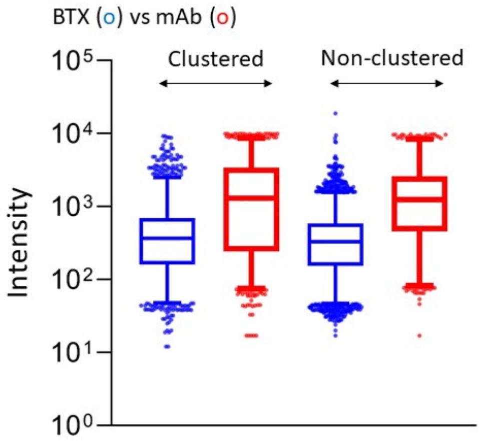

Figure S9: Intensity of clustered and non-clustered BTX- and mAb-labeled nAChR localizations from STED-imaged samples. The box plot (log scale) shows the interquartile range, with the median represented in the middle. The extremes represent the 2.5th and the 97.5th percentile. The small dots are outliers.

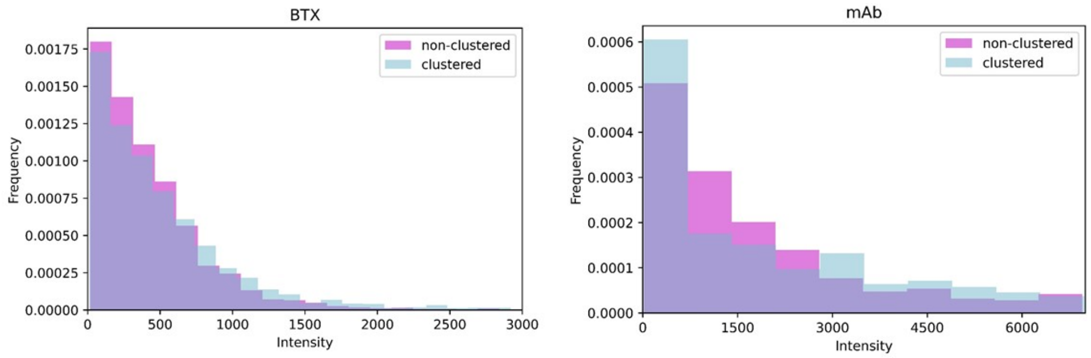

Figure S10: Probability density function (PDF) of the intensities of BTX- and mAb-labeled STED spots. A third color appears when the PDFs overlap.

ASTRICS without the CS step procedure

With every pair of clusters C_i and C_j in the second level of the hierarchy, ASTRICS skips CS steps due to the low dimensionality of the data, moves directly to ASTRI (an example of this step is shown in Supplementary Figure 11) and automatically obtains an α to determine the smallest α-shape enclosing all points from either C_i or C_j such that the similarity matrix is as sparse as possible. Next, ASTRICS generates a triangulation T^{ij}, conformed by tuples specifying the triangles' vertices that belong to the clusters of the localizations of C_i and C_j that exactly define the domain of the previously defined α-shape. Finally, if T_i^{ij} and T_j^{ij} are the triangles with vertices belonging to C_i and C_j respectively, it calculates $ASTRICS(C_i, C_j) = |T_i^{ij} \cap T_j^{ij}|/|T_i^{ij} \cup T_j^{ij}|$ i.e., the percentage of triangles sharing vertices from both clusters.

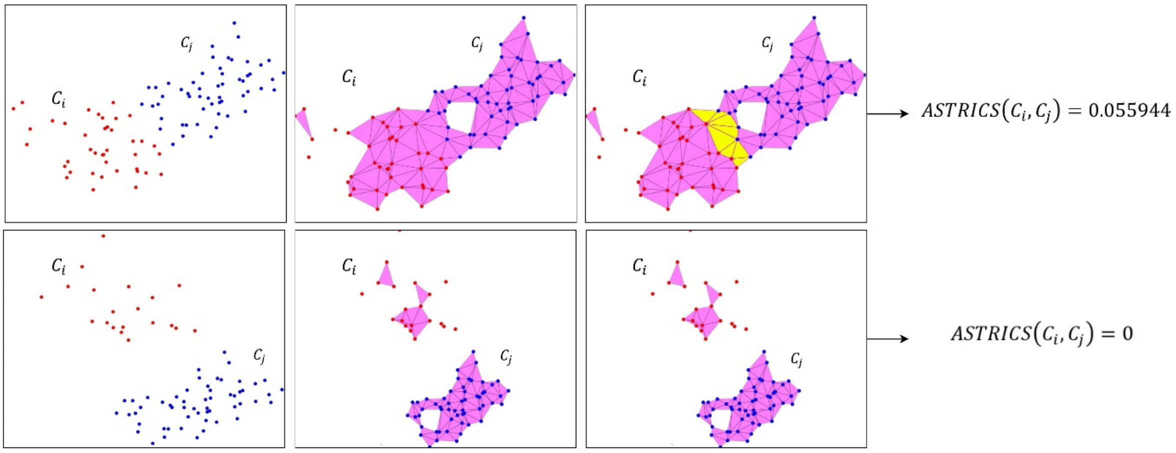

Figure S11: Examples of the ASTRI step in two different pairs of clusters. ASTRI takes a pair of seed clusters C_i (red) and C_j (blue) and calculates its ASTRICS similarity. In the upper scatter plot, once the α-shape is obtained with the smallest possible area enclosing the data from either C_i or C_j and triangulates it, ASTRICS similarity is calculated. Because of the way that the α-shape is computed, minimizing the α-shape area, several cluster pairs exhibit ASTRICS similarity equal to zero, as shown in the scatter plots below.

Comparison between STORMGraph and STORMGraph + ASTRICS

ASTRICS (Alpha Shape TRIangulation in loCal Subspaces) is an inter-cluster similarity measure and a computational geometry-based algorithm to visualize and cluster high dimensional data that consists of two steps: CS and ASTRI. It receives as input seed clusters of the data that are generated by any clustering algorithm (for example, K-Means) such that the number of seed clusters is greater than the expected number of clusters to detect and outputs a similarity matrix M such that $M_{i,j}$ is the ASTRICS similarity between seed clusters C_i and C_j. The first step seeks to reduce dimensions of input data (CS), while the second one computes ASTRICS similarity for each reduced seed cluster pair (ASTRI). Since we are working with two-dimensional data (i.e., 2D localizations) and the sparsity of the similarity matrix could be of interest, we present how ASTRICS could be used for low dimensional data skipping CS step and only using ASTRI step with STORMGraph to get seed clusters. In Figure S11 we show examples of the ASTRI step procedure for different pairs of clusters.

In the present work, seed clusters are generated from the second level of STORMGraph cluster hierarchy. If the first level were used, no clusters would be detected because ASTRICS would not be able to measure inter-cluster similarities distinct from zero in such small clusters. As shown in Figure S12, the sparsity of the similarity matrix helps detect cluster areas much smaller than those detected by STORMGraph alone ($p < 0.0001$). ASTRICS discards those nanoclusters that do not fulfill a minimum threshold number of molecules (> 15, the same number as in STORMGraph). In addition, we found a lower percentage of molecules occurring

in clusters than with the original STORMGraph algorithm ($p < 0.05$) (see Figure S12). However, particle densities from STORMGraph + ASTRICS were twice as high as those obtained with STORMGraph alone ($p < 0.0001$), although the number of molecules per nanocluster and centroid distances showed smaller values with STORMGraph + ASTRICS than with STORMGraph ($p < 0.0001$).

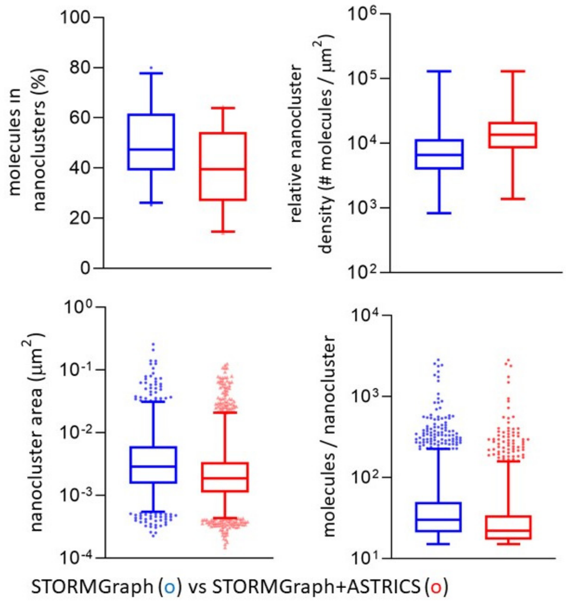

Figure S12. Main parameters derived from the STORMGraph analysis and STORMGraph + ASTRICS of the STORM experimental data. The whiskers represent the interquartile range and the median. The extremes of the line indicate the 2.5 – 97.5 percentile. The dots are outliers. All plots (except for the one depicting molecules in nanoclusters) are in log scale.

We found noticeable statistical differences between STORMGraph and its modification when comparing BTX- and mAb-tagged samples, as shown in the Figure S13. Nanocluster areas identified with STORMGraph + ASTRICS were ~3.1 times smaller in BTX-tagged samples and ~1.6 times smaller in mAb-labeled samples than those depicted with the STORMGraph technique ($p < 0.0001$). In addition, densities in BTX and mAb samples were ~1.8 and ~1.4 times higher upon application of the STORMGraph + ASTRICS algorithms ($p < 0.0001$), respectively. Centroid distances were shorter with STORMGraph + ASTRICS than with STORMGraph alone in both labels BTX- ($p < 0.0001$) and mAb-labeled samples ($p < 0.01$). Number of clustered molecules and molecules per cluster were lower in both labels in STORMGraph + ASTRICS than STORMGraph ($p < 0.0001$).

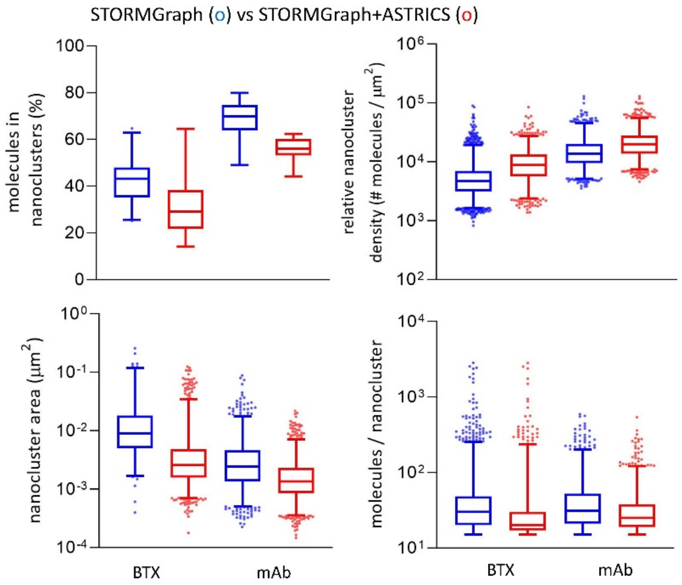

Figure S13: Main parameters derived from the STORMGraph and STORMGraph+ASTRICS analyses of the STORM experimental data of BTX- and mAb-labeled specimens. The whiskers represent the interquartile range and the median. The extremes of the line indicate the 2.5 – 97.5 percentile. The dots are outliers. All plots (except for the one showing molecules in nanoclusters) are in log scale.

Cluster ellipticity analysis

The main purpose of this algorithm is to determine the ellipticity of a given cluster. This is of interest when we compare the shape of the clusters in the samples imaged with either STED or STORM. Algorithms based on least-squares methods are not suitable for this purpose because one must fit an ellipse covering the entire cluster and not just its boundary.

The algorithm (Figure S14) receives as input a set of data $\{x_0, x_1, \dots, x_n\}$ such that $x_i \in \mathbb{R}^2$. Its outputs are the elliptic parameters (axes a and b) which can be used to measure the eccentricity ε of the ellipse. First, x_a and x_b, the farthest points of the dataset, are found (Suppl. Figure 14.b) and a cluster rotation is performed at the middle between these points until both are horizontally aligned (Suppl. Figure 14.c). $x_a{}'$ and $x_b{}'$ are the resulting rotated positions of x_a and x_b respectively, $a = \frac{\|x_a{}' - x_b{}'\|}{2}$. To calculate b we take the uppermost and farthest down points of the rotated cluster, $x_c' = (x'_{c_1}, x'_{c_2})$ and $x_d' = (x'_{d_1}, x'_{d_2})$ respectively. Finally, we calculate $b = \frac{x'_{c_2} - x'_{d_2}}{2}$.

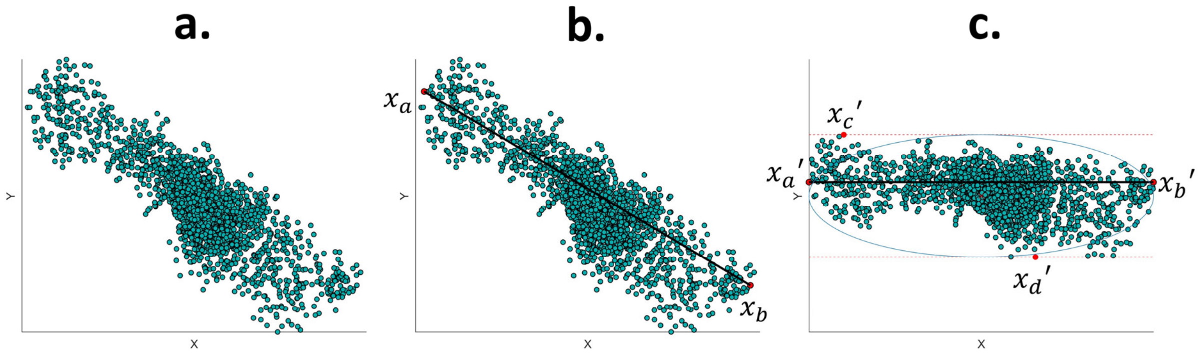

Figure S14: Cluster ellipticity analysis algorithm. **a.** First, we take any dataset of points. **b.** We next take the two farthest points of the dataset and rotate them until they are horizontally aligned. **c.** Finally, we calculate the main ellipse parameters a and b. The center of the ellipse on the original cluster and its inclination are also calculated.

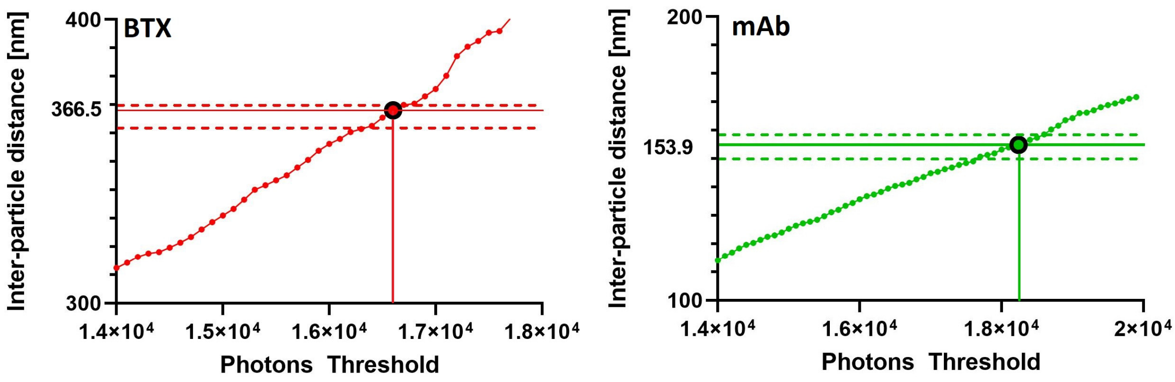

Figure S15: Inter-particle distance as a function of photons threshold. Continuous horizontal lines correspond to the inter-particle distance obtained from STED. Dashed horizontal lines corresponds to the upper and lower values of the confidence interval of inter-particle distance from STED. Vertical line represents photon threshold that gives the closest inter-particle distance to the distance obtained from STED.

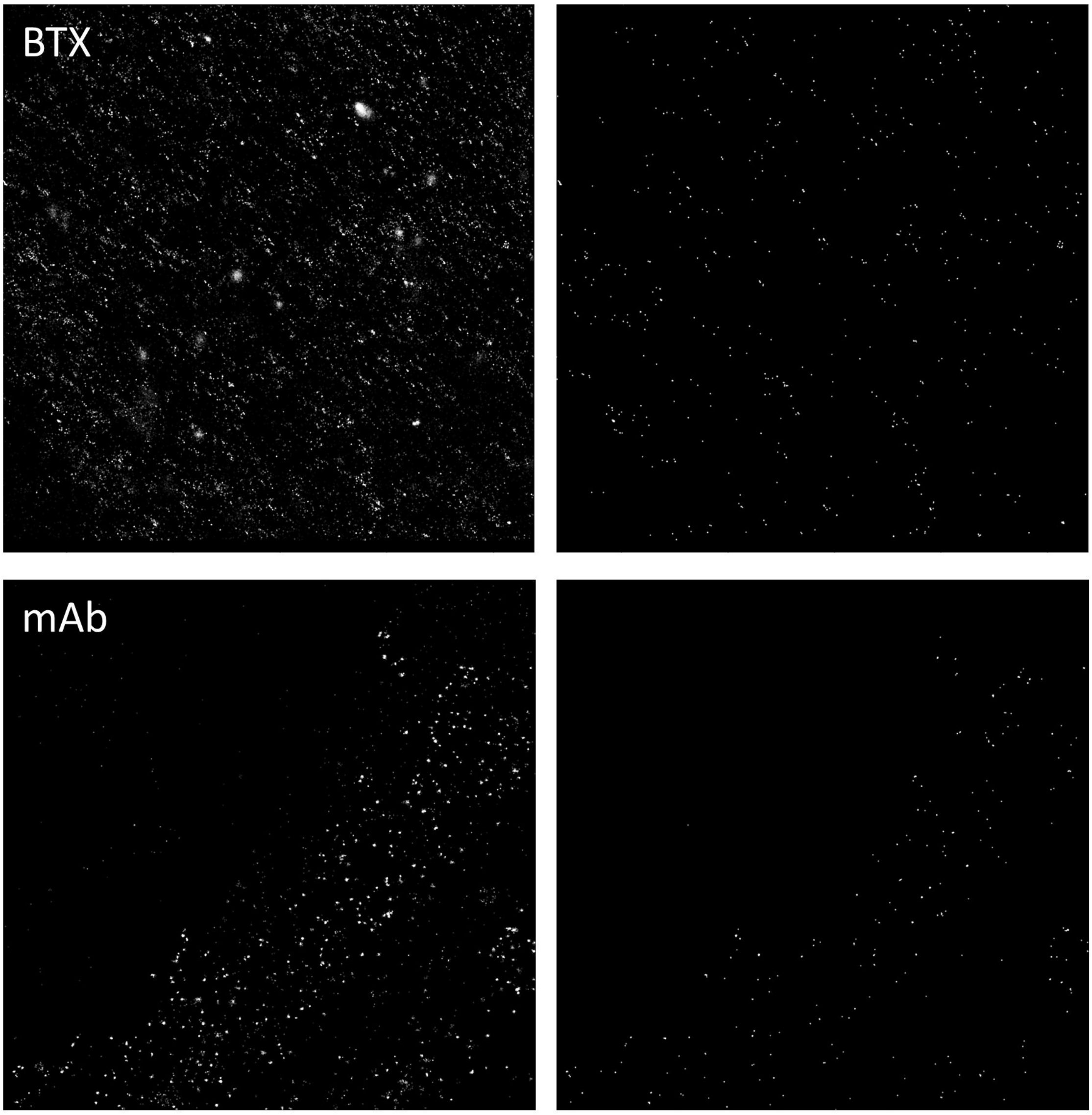

Figure S16: Original raw STORM localizations (*left*) and corresponding images upon filtering by the number of emitted photons (*right*). 10 µm x 10 µm ROIs are shown. In the case of BTX, localizations with less of 16,500 emitted photons where removed. In mAb, this threshold is 18,000.

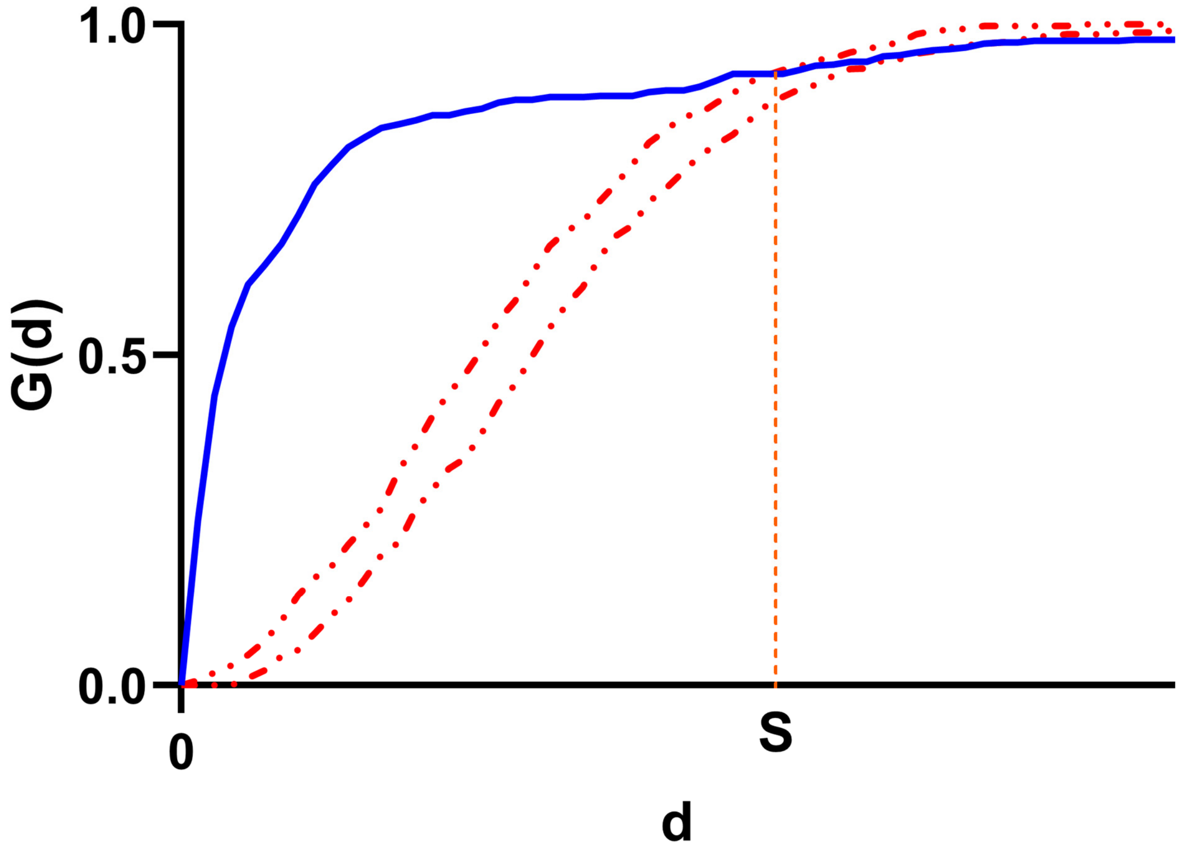

Figure S17: Example of the G(d) function. The blue line corresponds to the G(d) function and the upper and lower dotted-dashed lines correspond to the upper and lower values of the CI of the simulations, respectively. *S* is the distance at which clustering is no further detected.

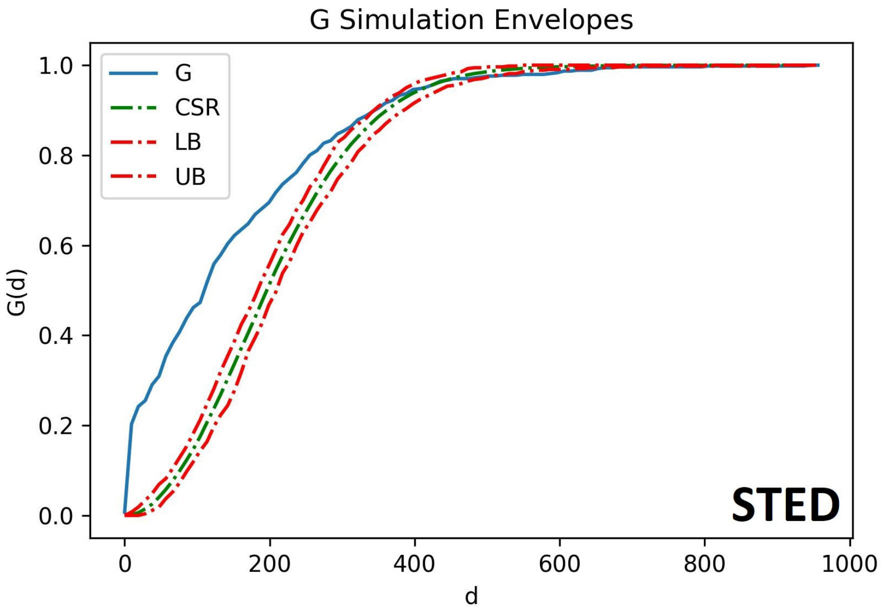

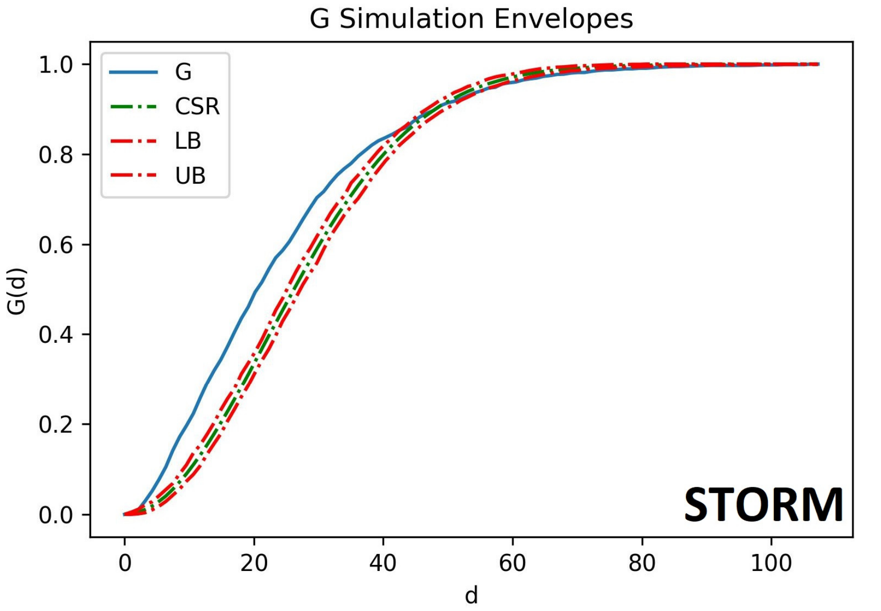

Figure S18: Comparison of G(d) function between STED and STORM. The G(d) function is above the upper-bound line (UB), indicating that there is clustering in both STORM and STED samples. Otherwise, the point pattern corresponds to a random process. Green dot-dashed line represents the G(d) function of a Complete Spatial Randomness (CSR) process. d is in nanometers.
