# Supplementary figures and images for: Mapping the Nicotinic Acetylcholine Receptor Nanocluster Topography at the Cell Membrane with STED and STORM Nanoscopies
Source: Int J Mol Sci. 2022 Sep 9;23(18):10435. doi: 10.3390/ijms231810435 (PMC9499342; doi:10.3390/ijms231810435)

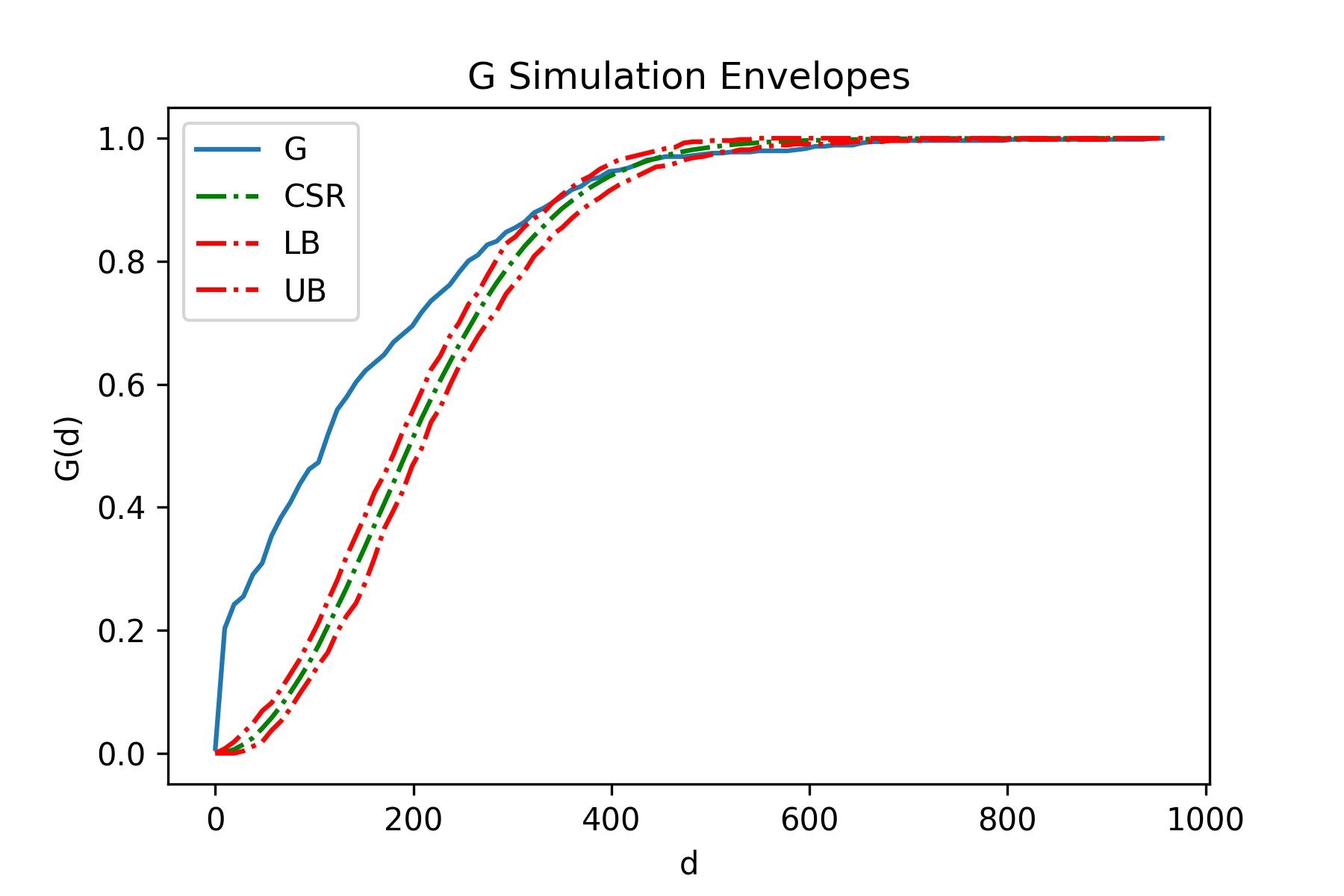

Supplement: Supplementary file 1 [file ijms-23-10435-s001.zip › supplementary File S1/STED G/sted_btx_1.jpg]

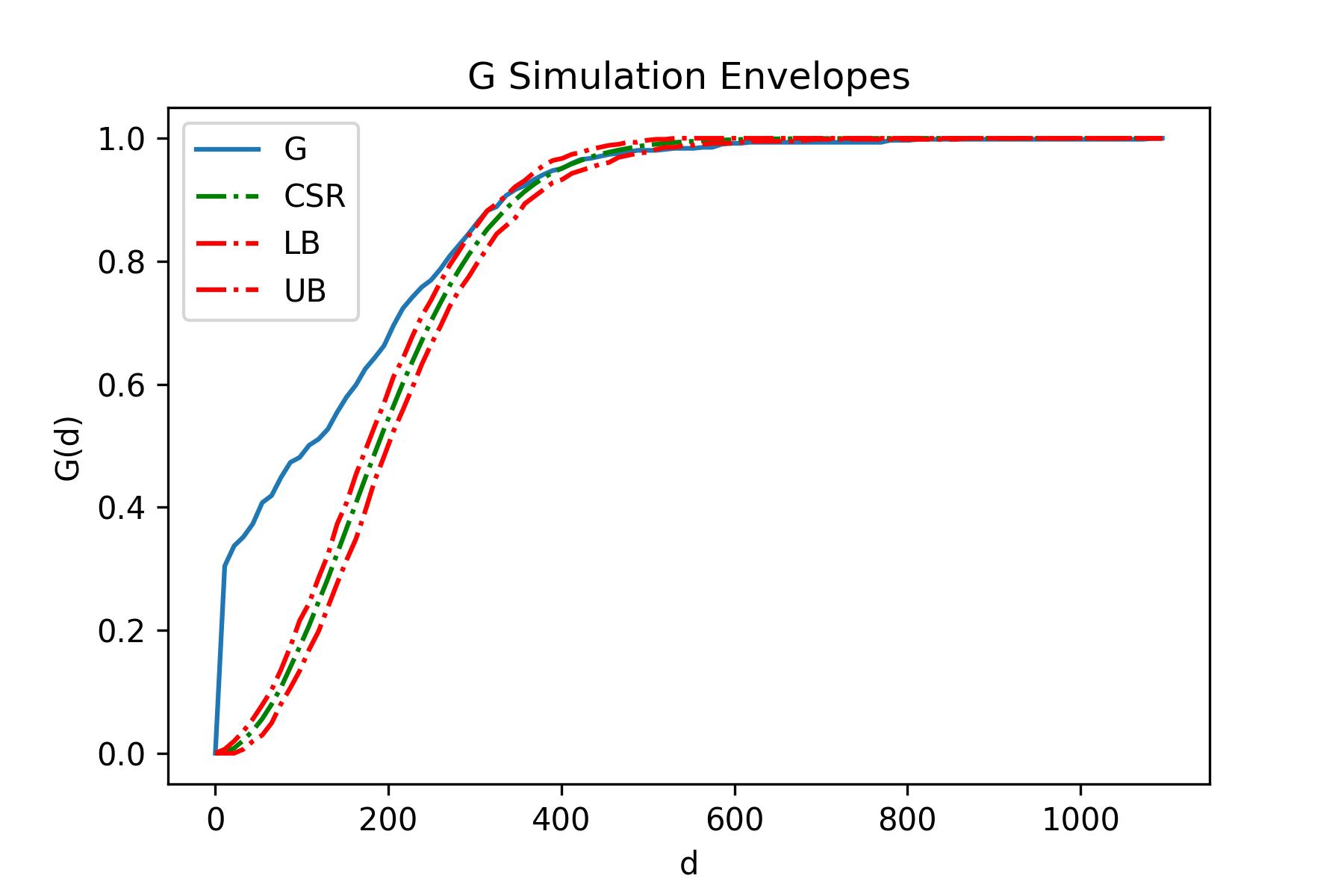

Supplement: Supplementary file 1 [file ijms-23-10435-s001.zip › supplementary File S1/STED G/sted_btx_10.jpg]

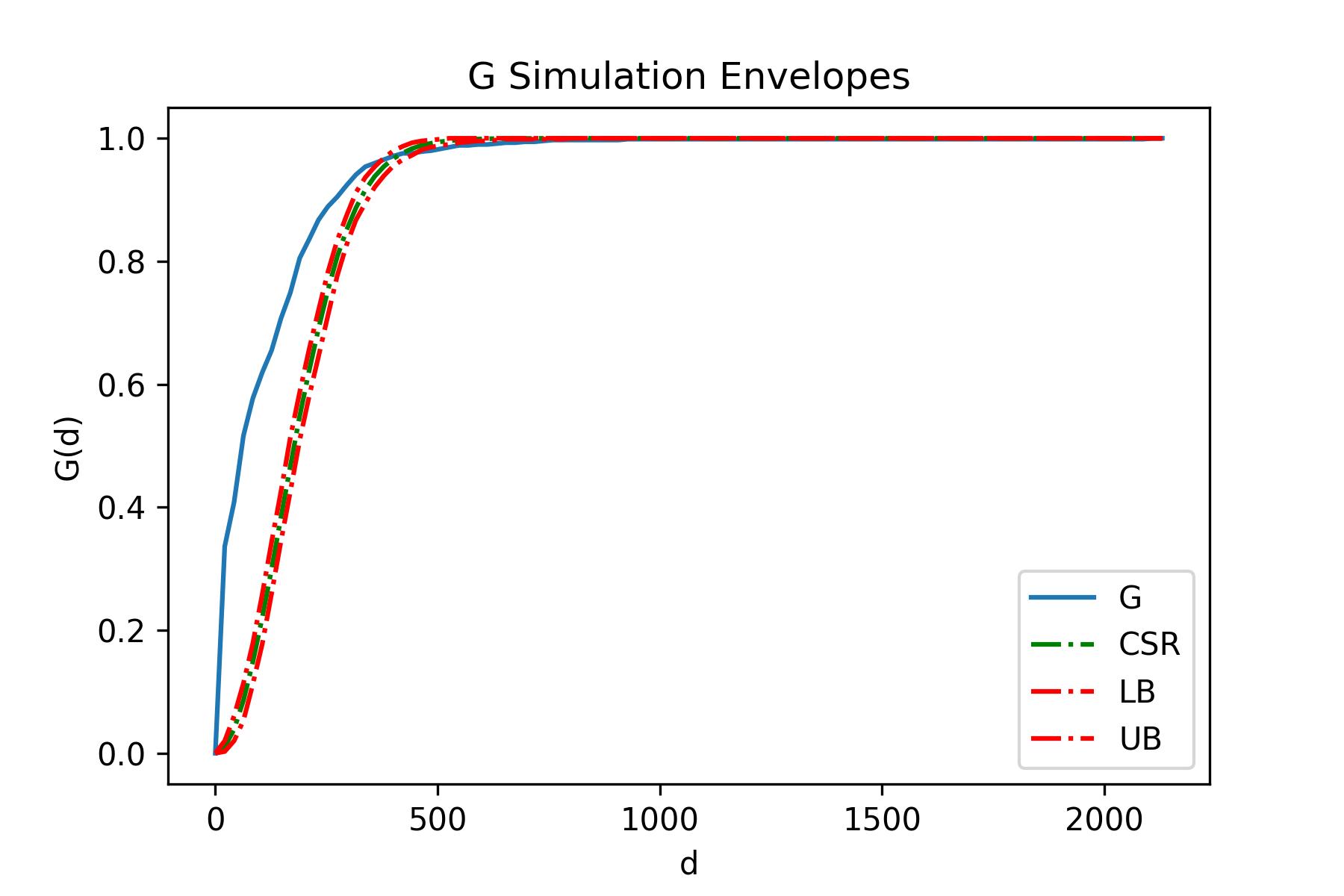

Supplement: Supplementary file 1 [file ijms-23-10435-s001.zip › supplementary File S1/STED G/sted_btx_11.jpg]

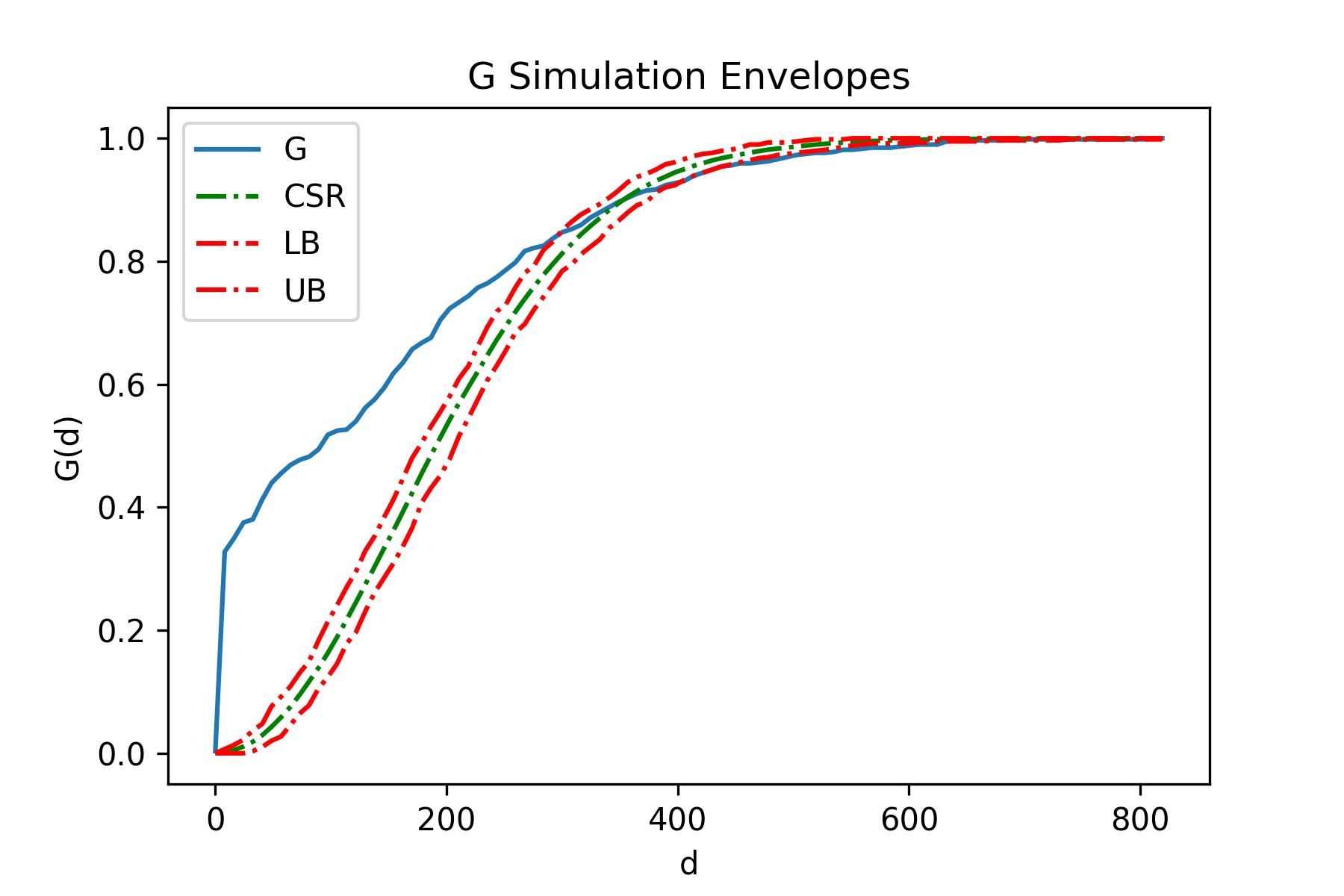

Supplement: Supplementary file 1 [file ijms-23-10435-s001.zip › supplementary File S1/STED G/sted_btx_12.jpg]

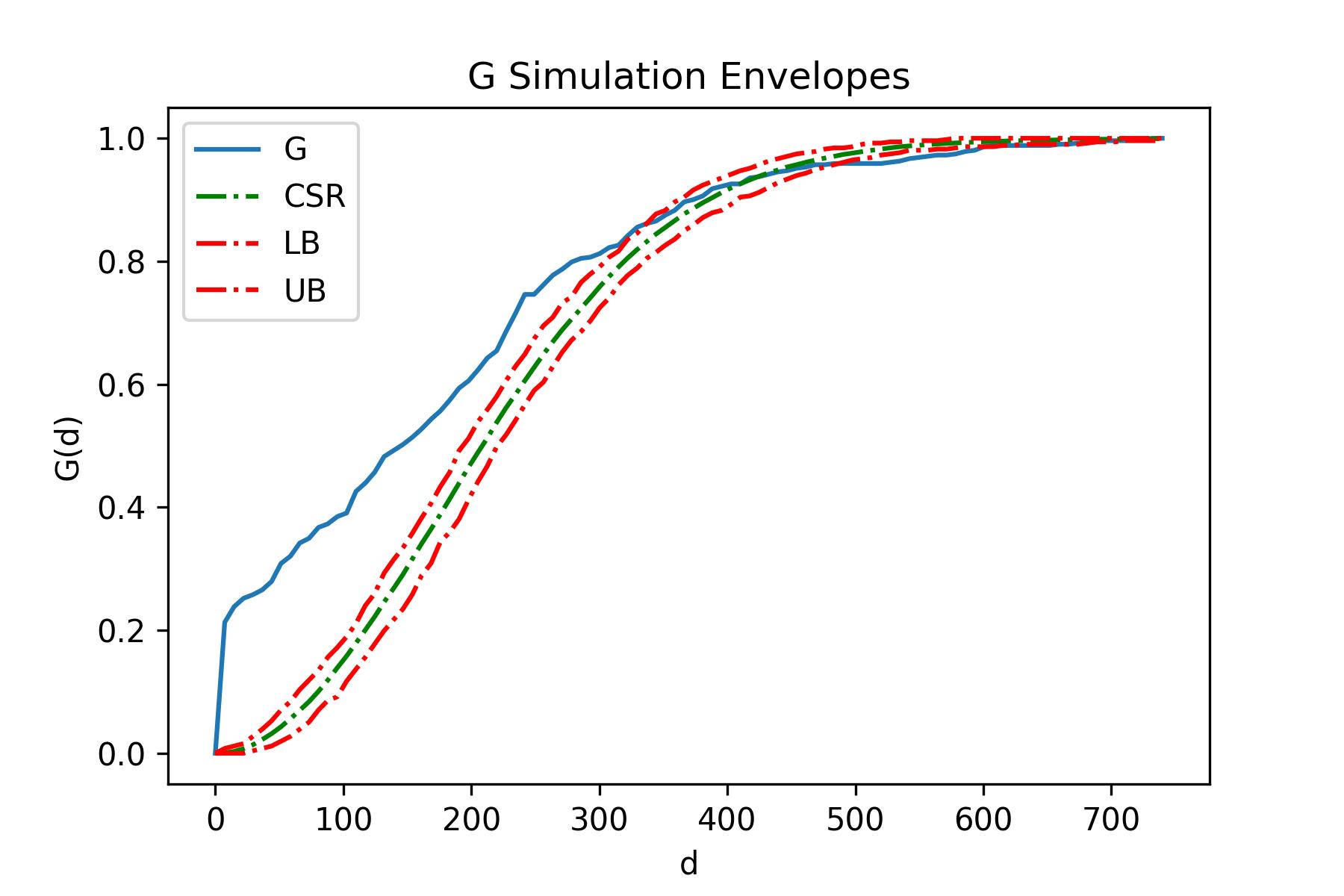

Supplement: Supplementary file 1 [file ijms-23-10435-s001.zip › supplementary File S1/STED G/sted_btx_13.jpg]

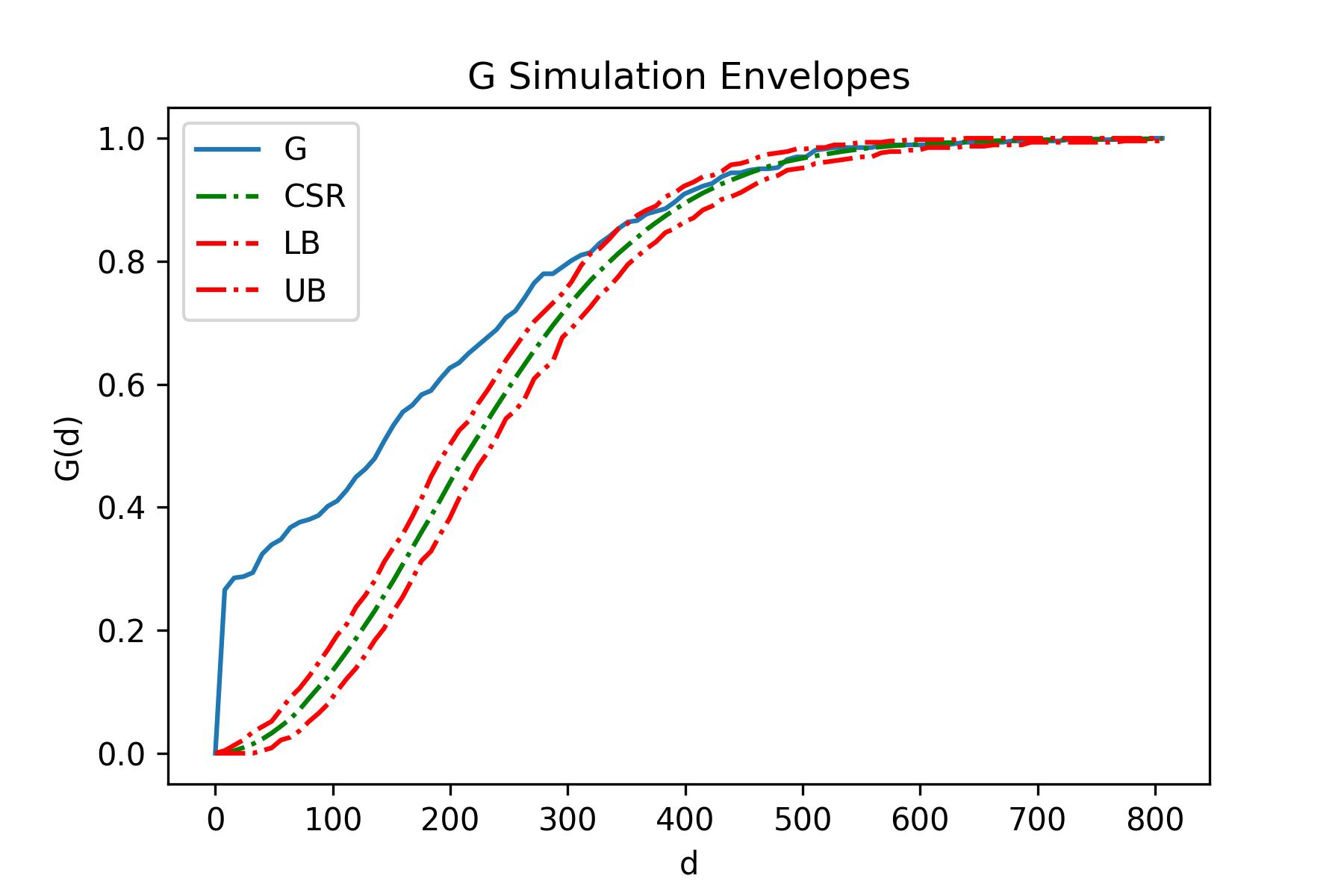

Supplement: Supplementary file 1 [file ijms-23-10435-s001.zip › supplementary File S1/STED G/sted_btx_14.jpg]

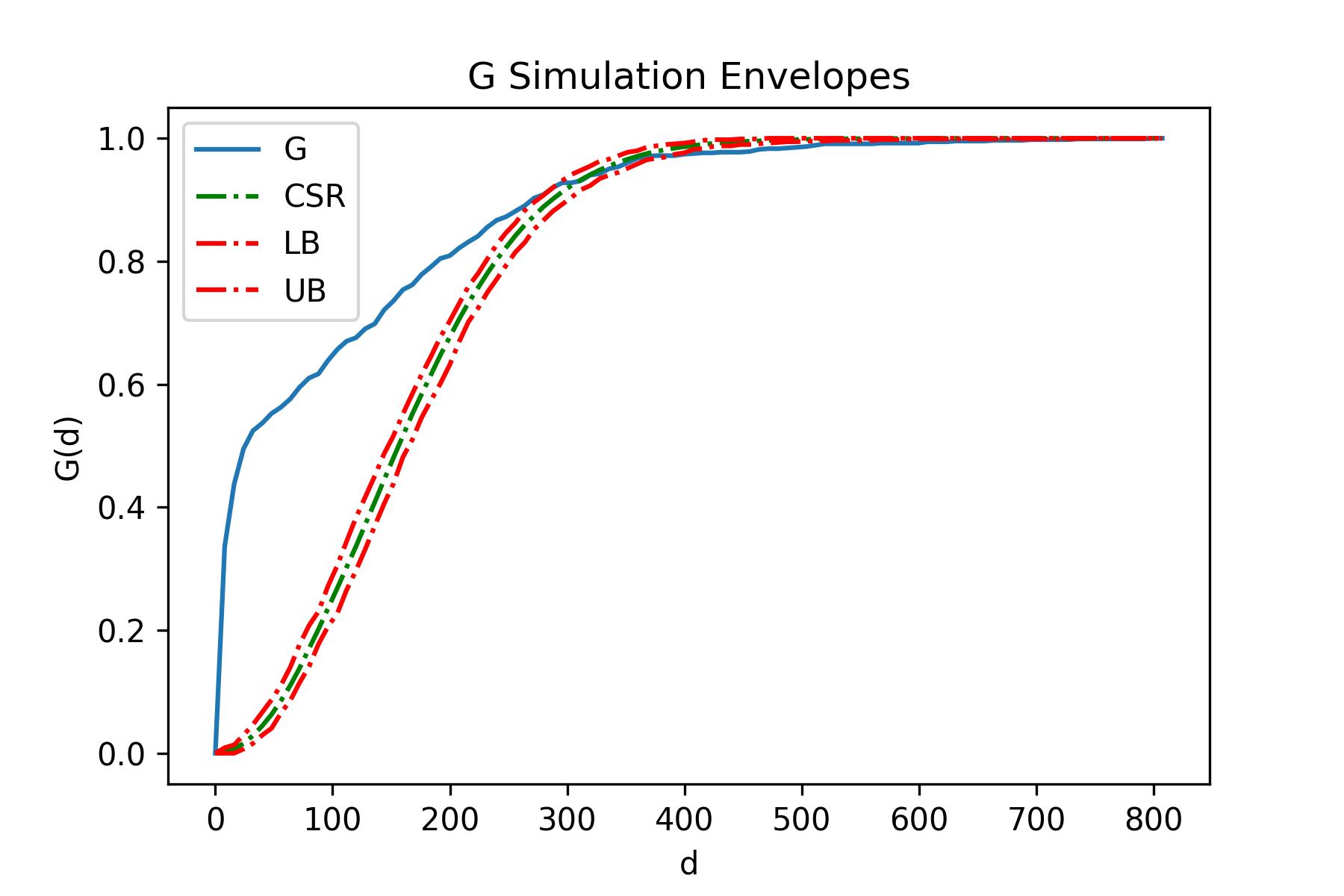

Supplement: Supplementary file 1 [file ijms-23-10435-s001.zip › supplementary File S1/STED G/sted_btx_2.jpg]

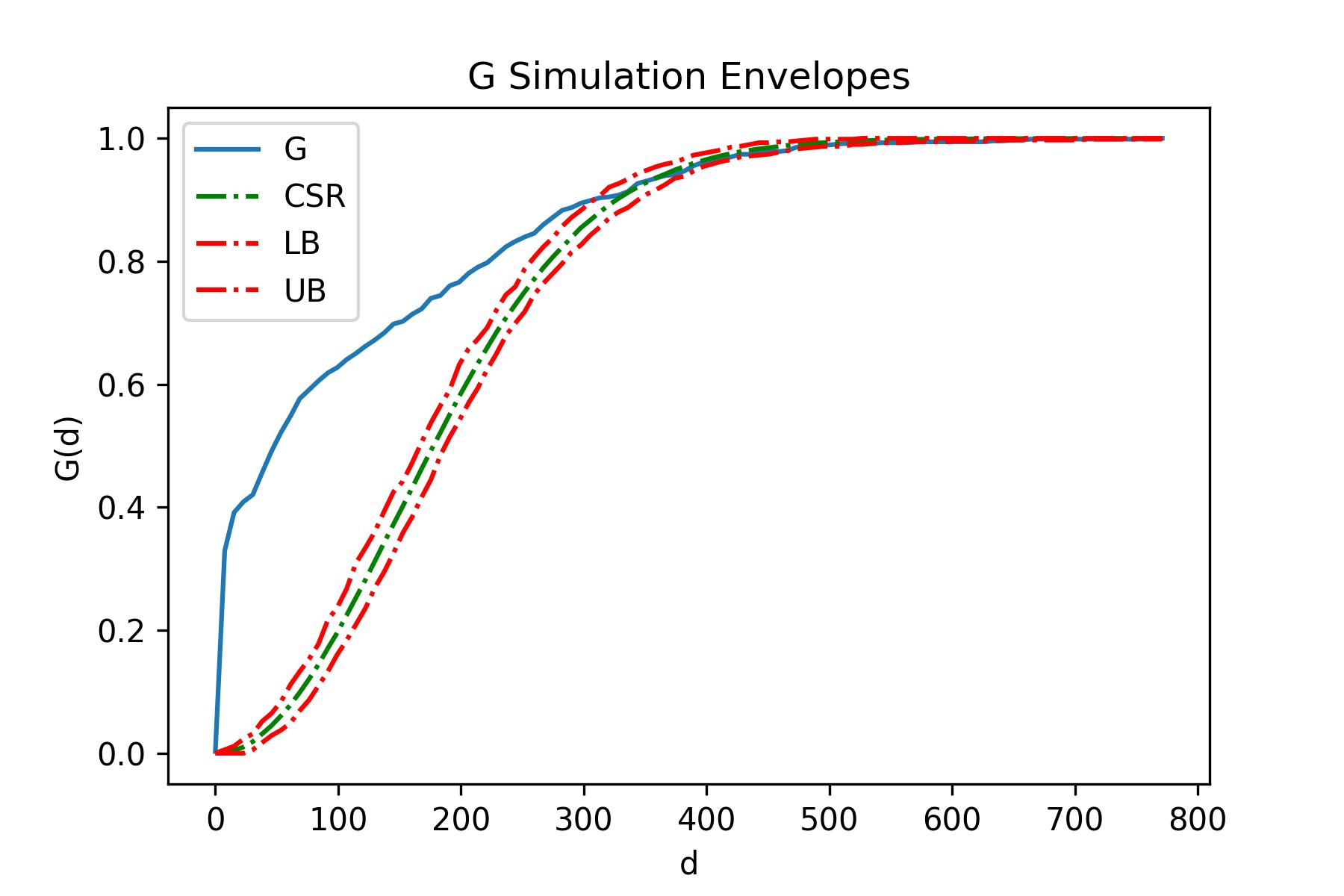

Supplement: Supplementary file 1 [file ijms-23-10435-s001.zip › supplementary File S1/STED G/sted_btx_3.jpg]

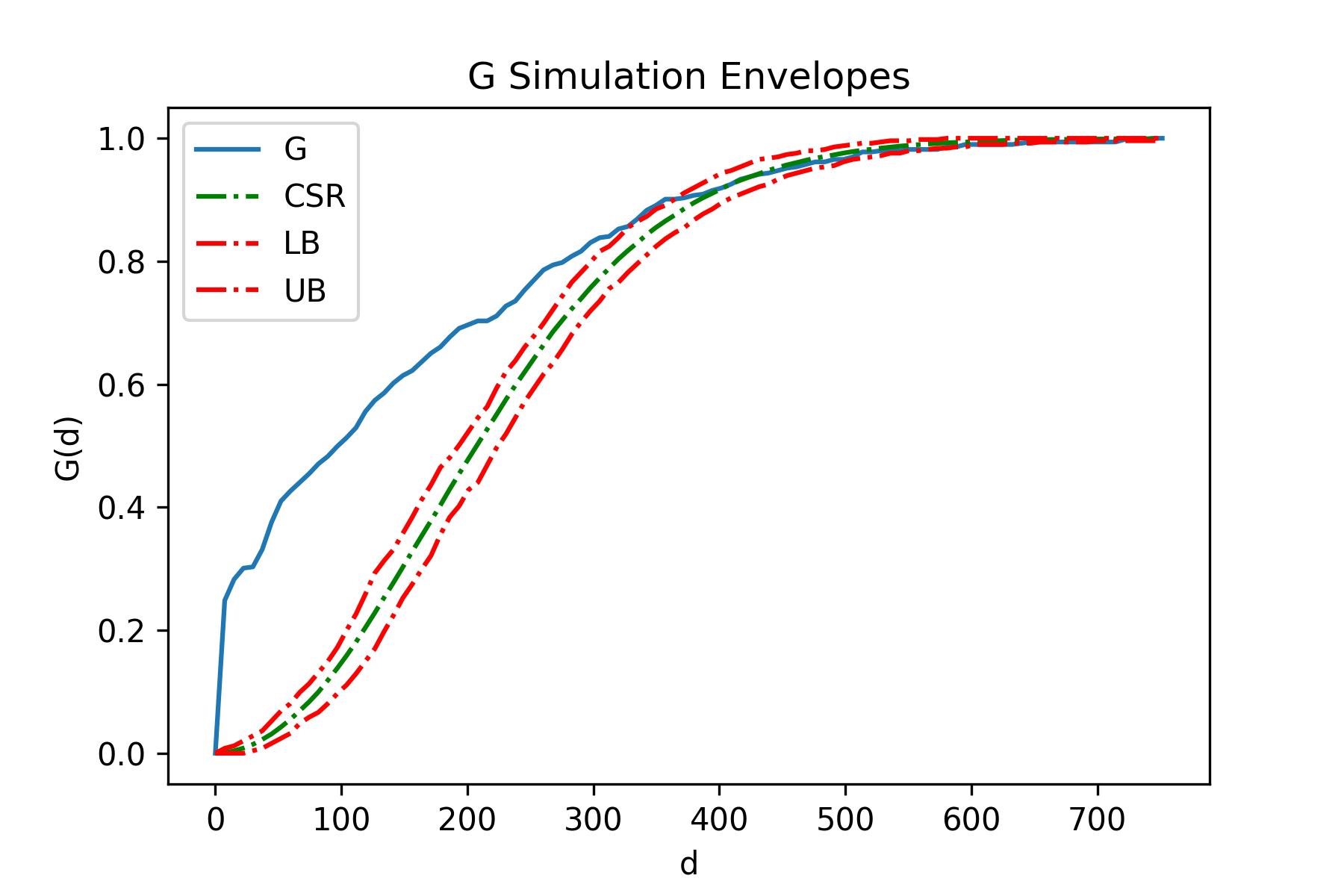

Supplement: Supplementary file 1 [file ijms-23-10435-s001.zip › supplementary File S1/STED G/sted_btx_4.jpg]

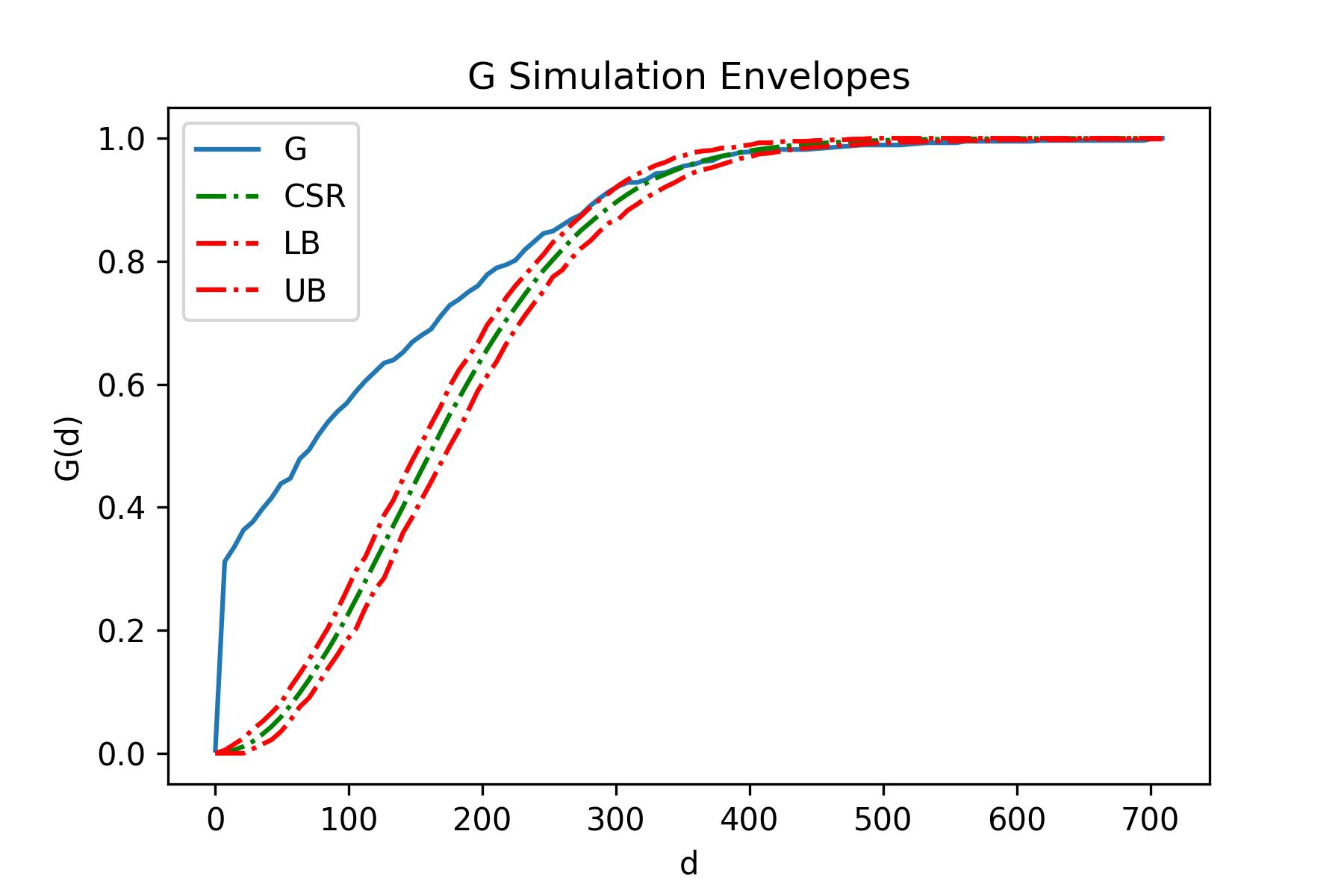

Supplement: Supplementary file 1 [file ijms-23-10435-s001.zip › supplementary File S1/STED G/sted_btx_5.jpg]

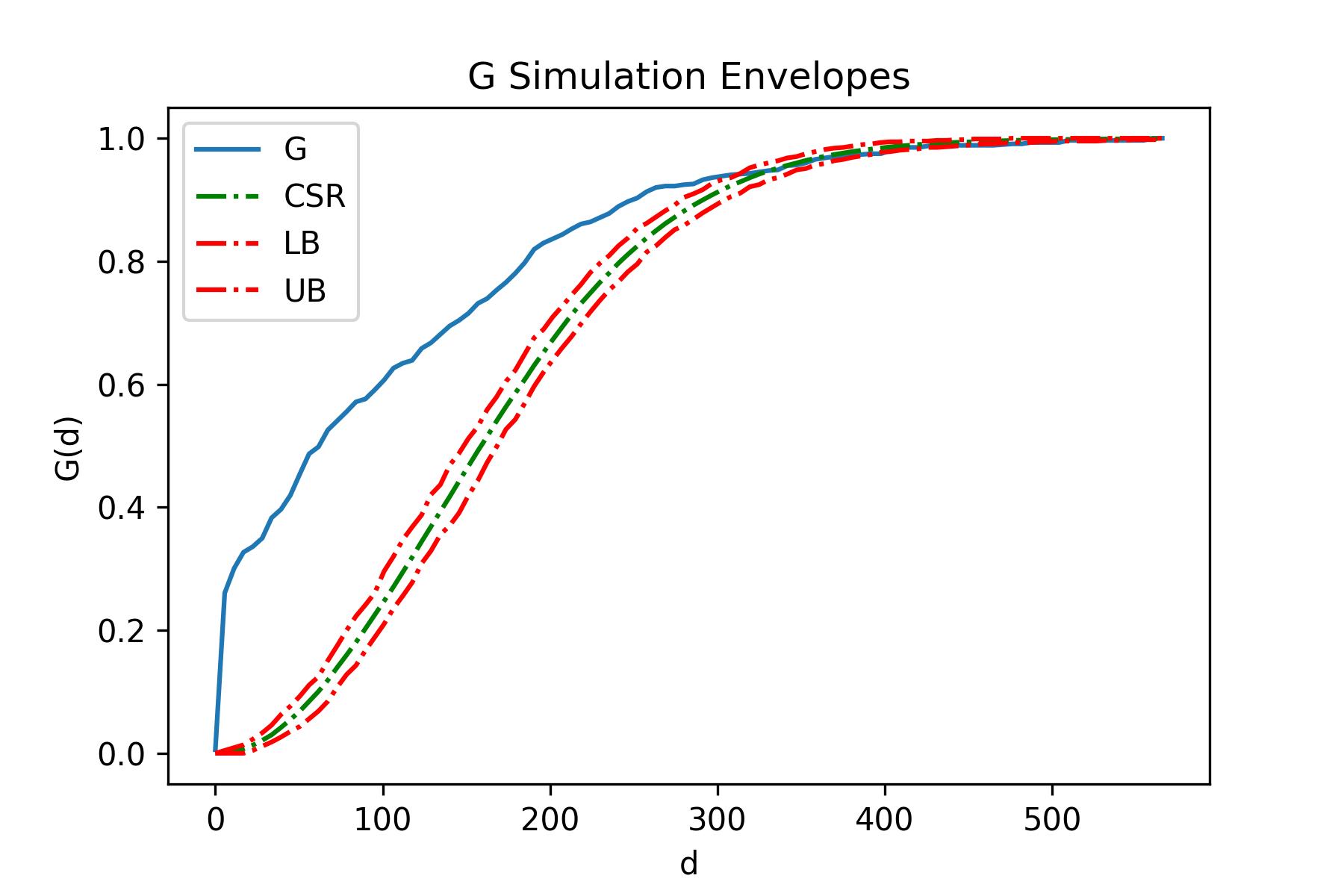

Supplement: Supplementary file 1 [file ijms-23-10435-s001.zip › supplementary File S1/STED G/sted_btx_6.jpg]

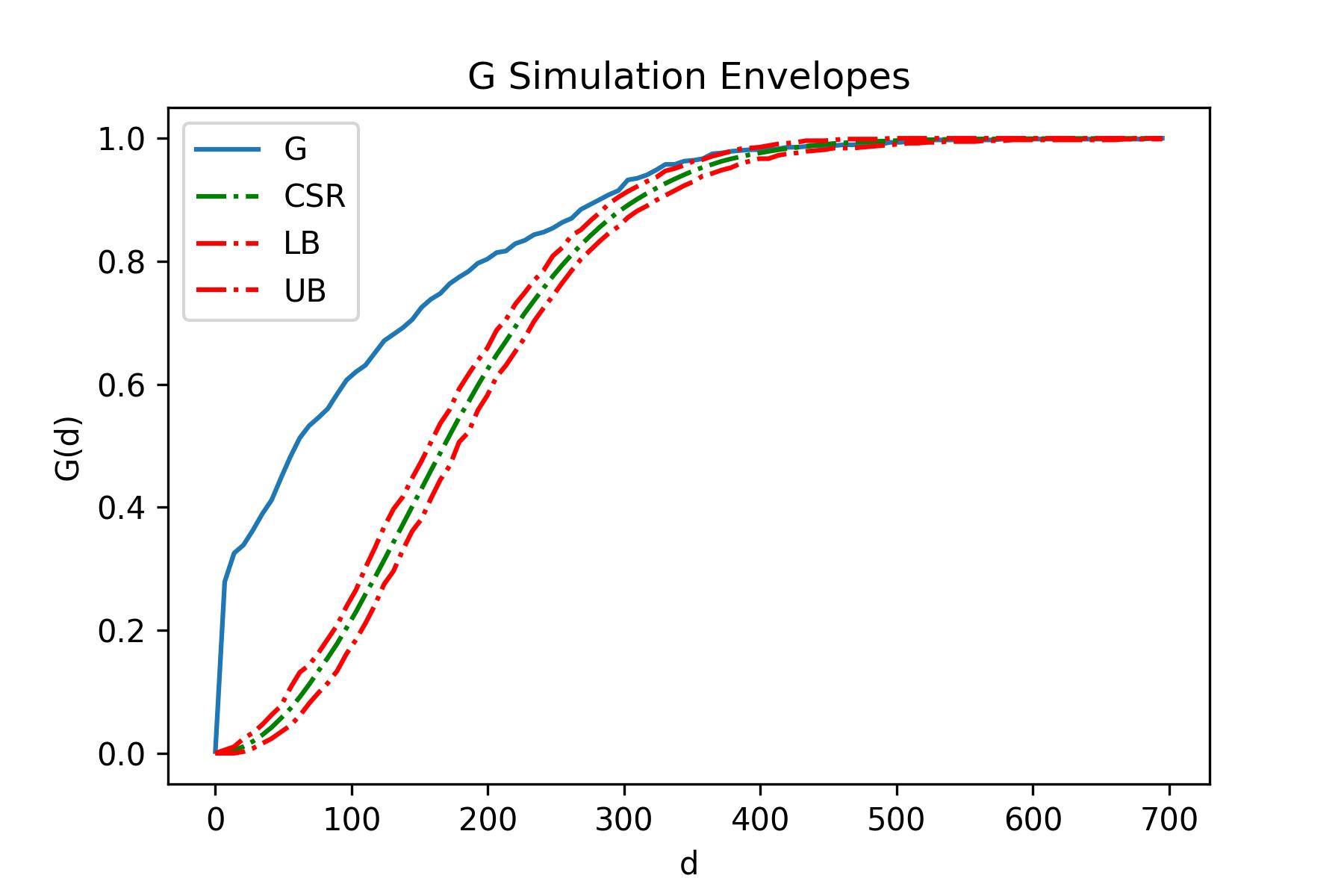

Supplement: Supplementary file 1 [file ijms-23-10435-s001.zip › supplementary File S1/STED G/sted_btx_7.jpg]

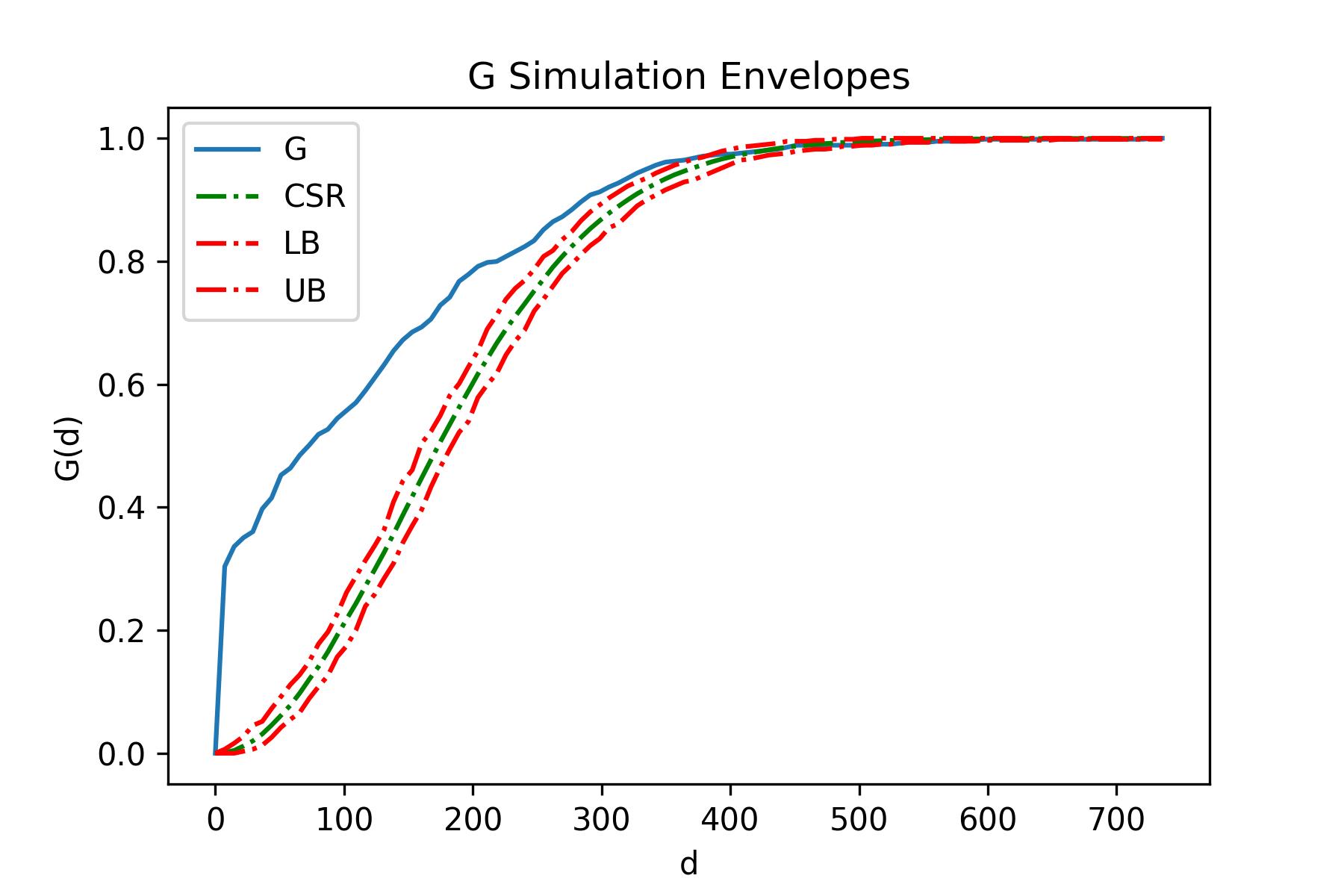

Supplement: Supplementary file 1 [file ijms-23-10435-s001.zip › supplementary File S1/STED G/sted_btx_8.jpg]

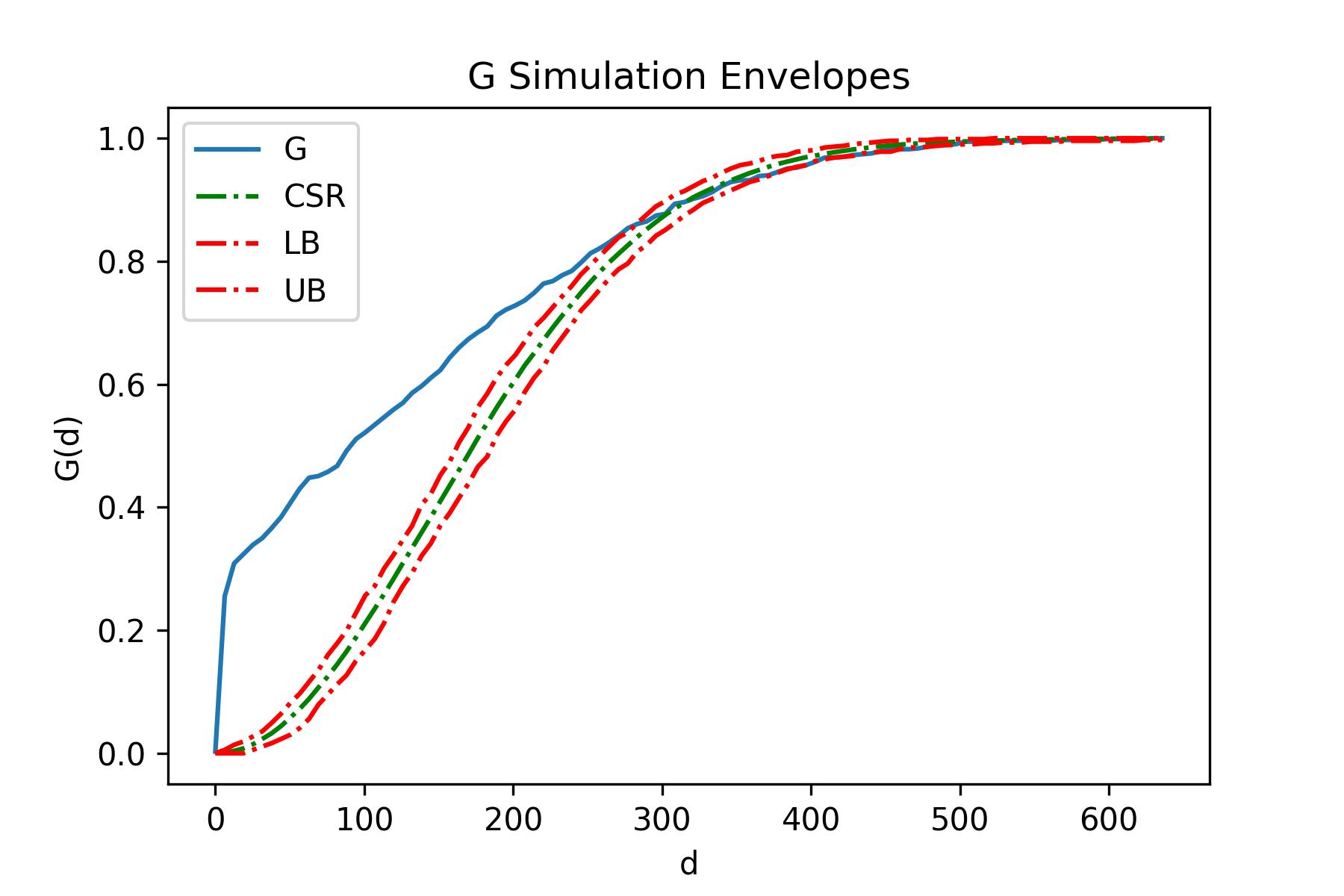

Supplement: Supplementary file 1 [file ijms-23-10435-s001.zip › supplementary File S1/STED G/sted_btx_9.jpg]

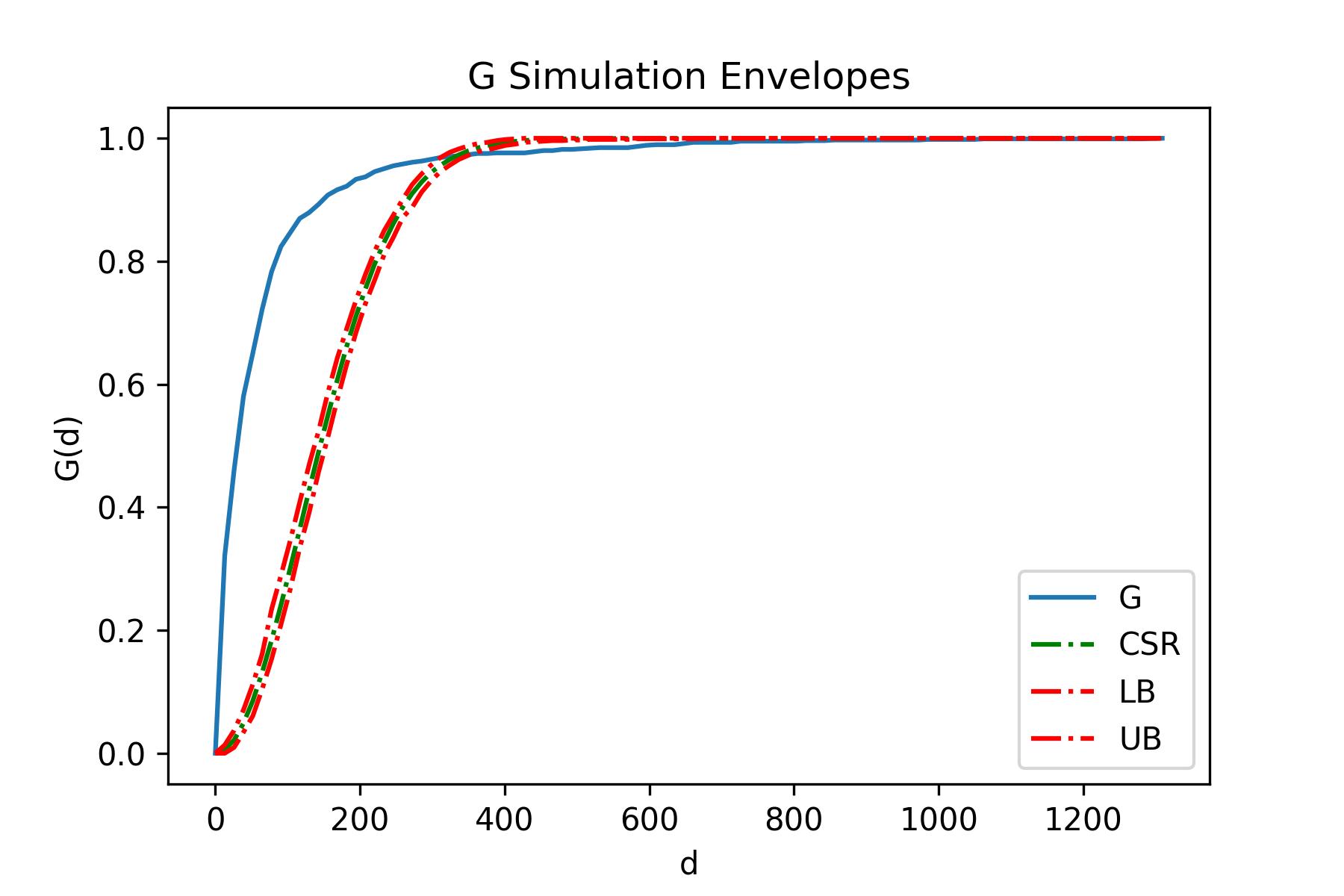

Supplement: Supplementary file 1 [file ijms-23-10435-s001.zip › supplementary File S1/STED G/sted_mab_15.jpg]

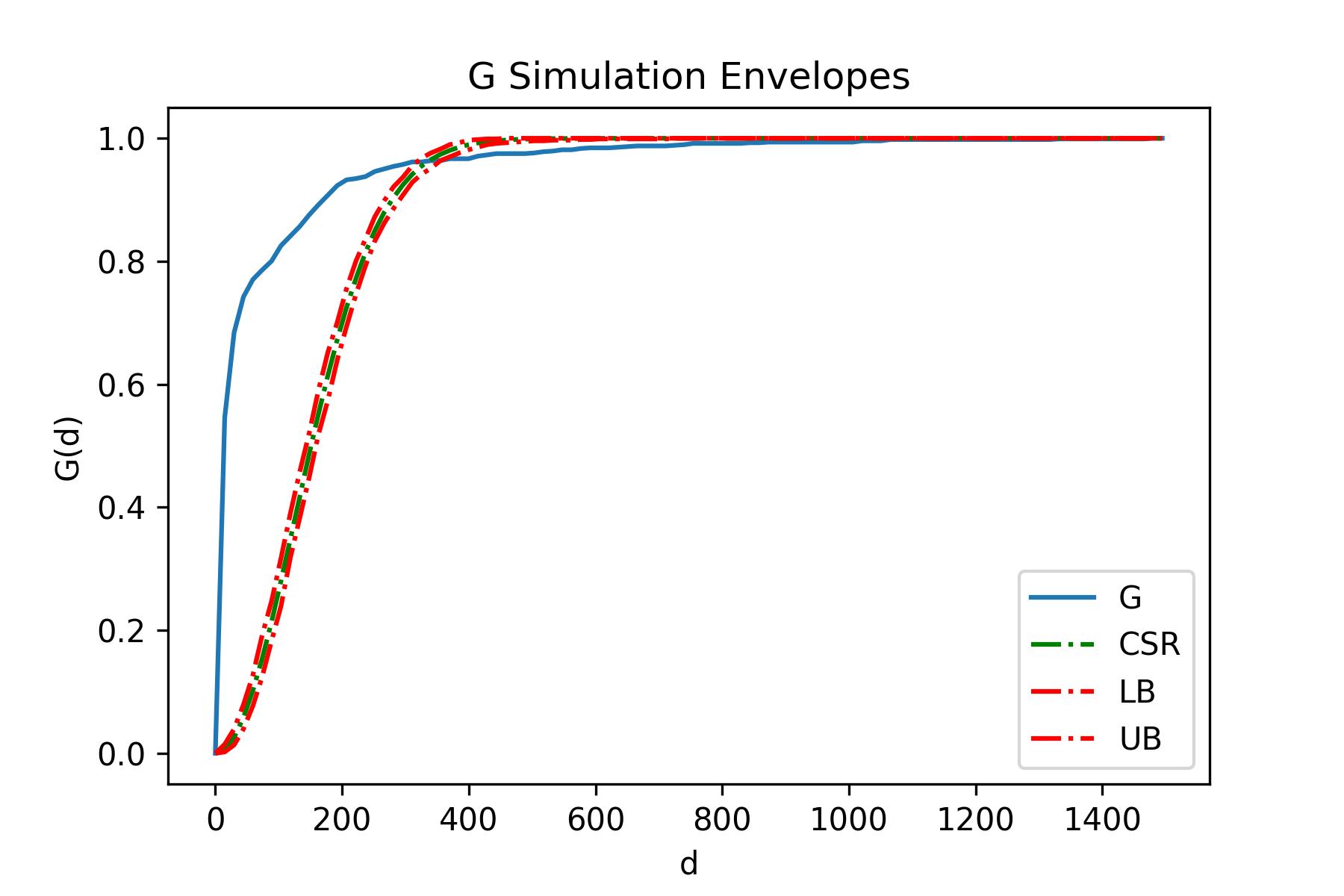

Supplement: Supplementary file 1 [file ijms-23-10435-s001.zip › supplementary File S1/STED G/sted_mab_16.jpg]

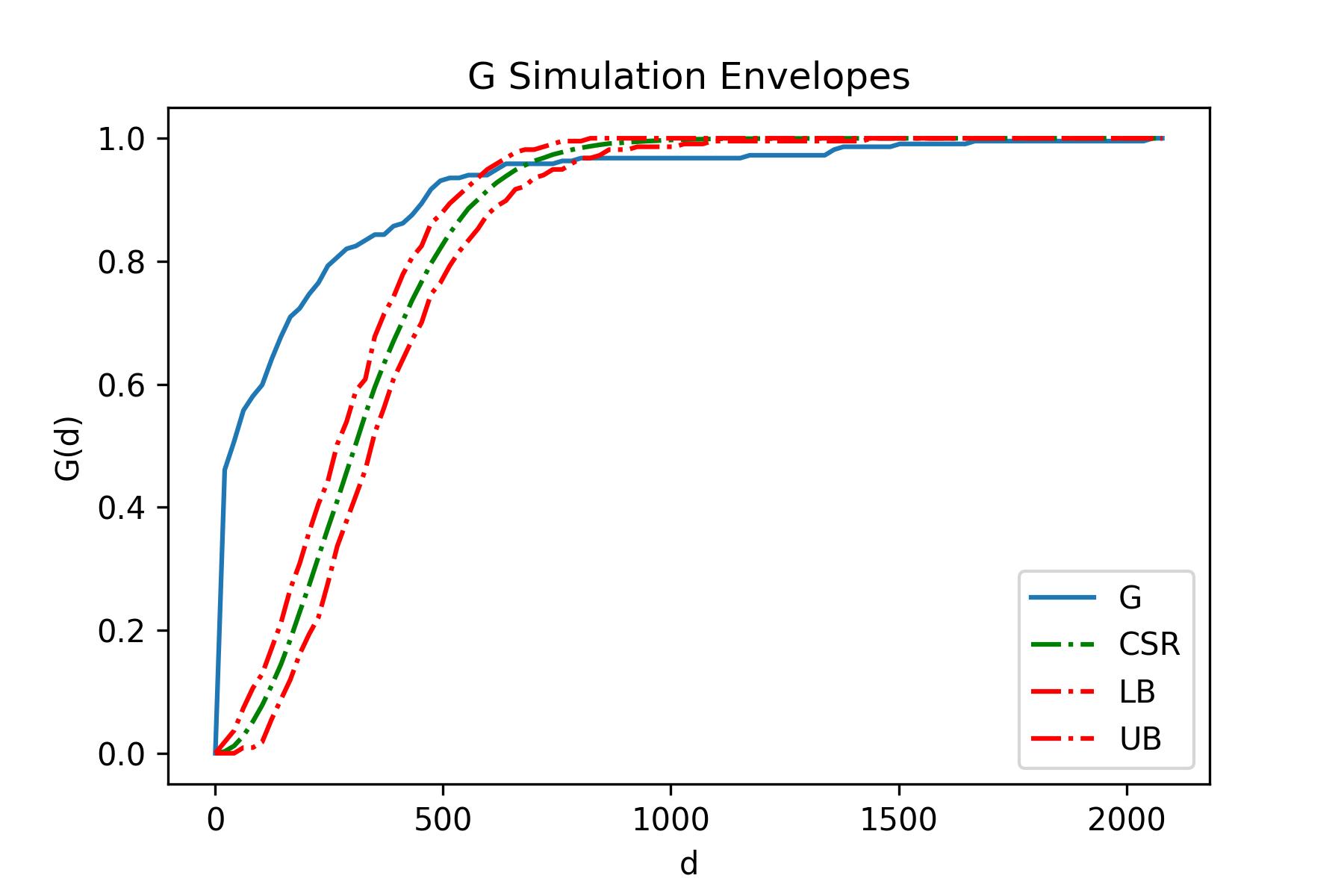

Supplement: Supplementary file 1 [file ijms-23-10435-s001.zip › supplementary File S1/STED G/sted_mab_17.jpg]

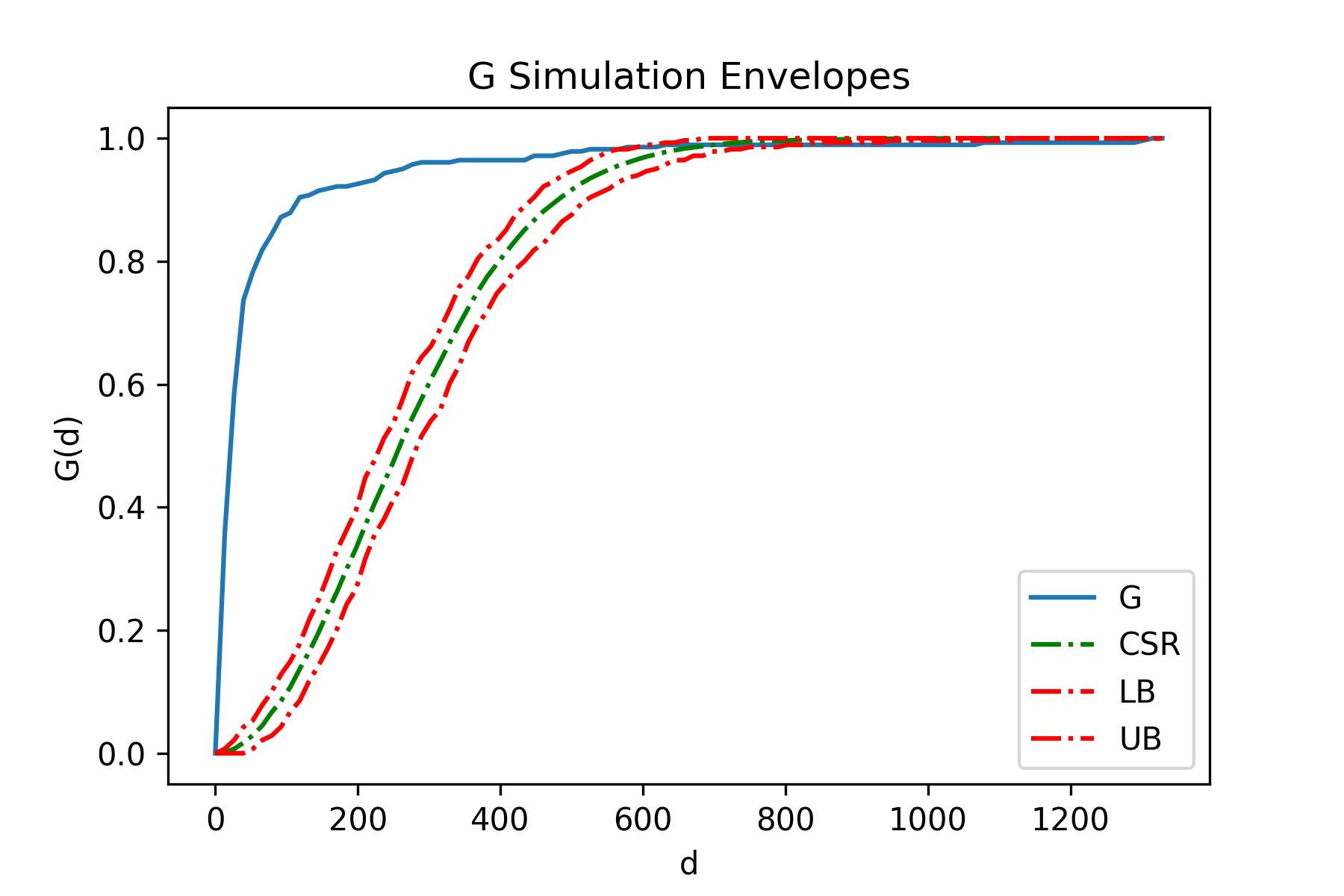

Supplement: Supplementary file 1 [file ijms-23-10435-s001.zip › supplementary File S1/STED G/sted_mab_18.jpg]

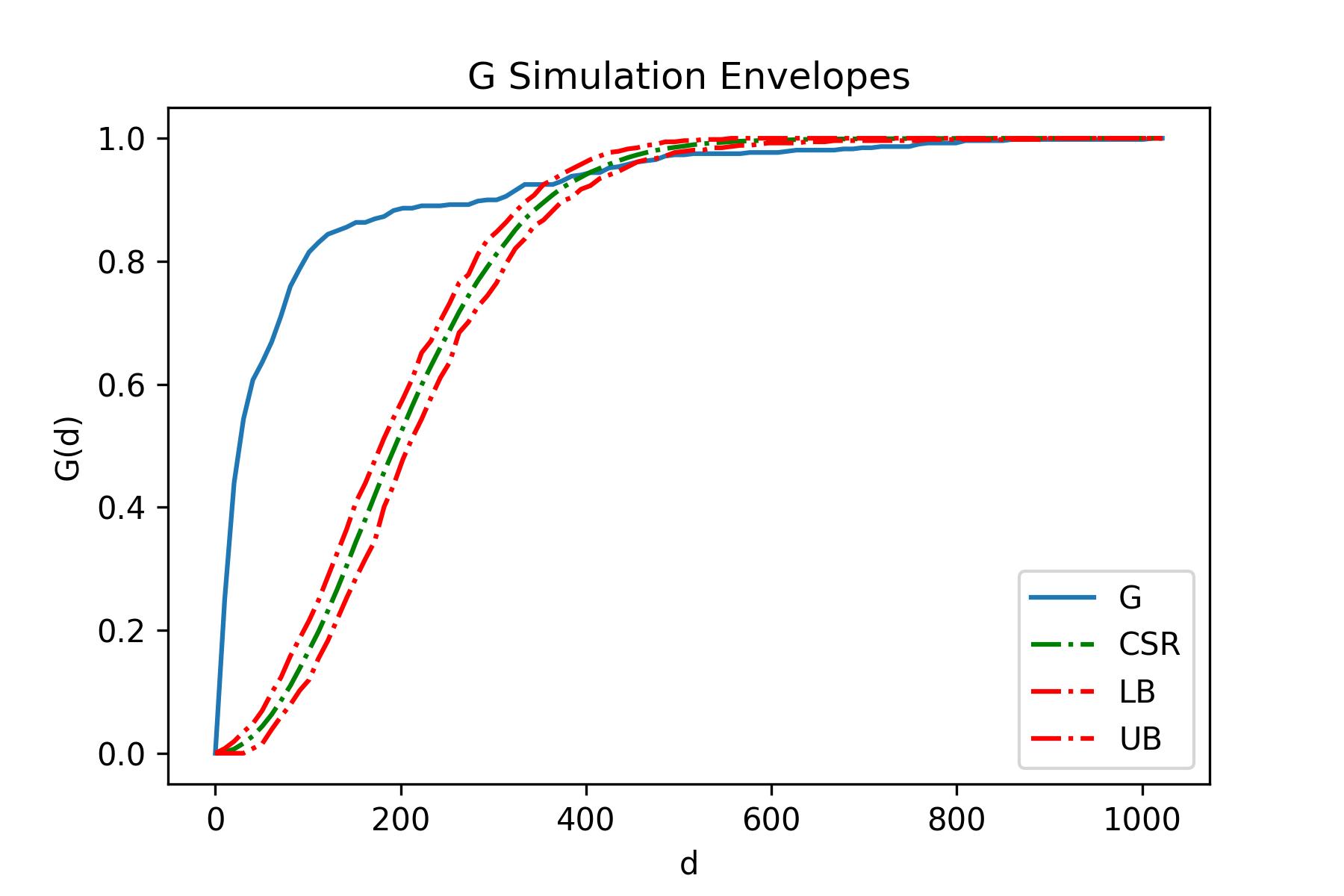

Supplement: Supplementary file 1 [file ijms-23-10435-s001.zip › supplementary File S1/STED G/sted_mab_19.jpg]

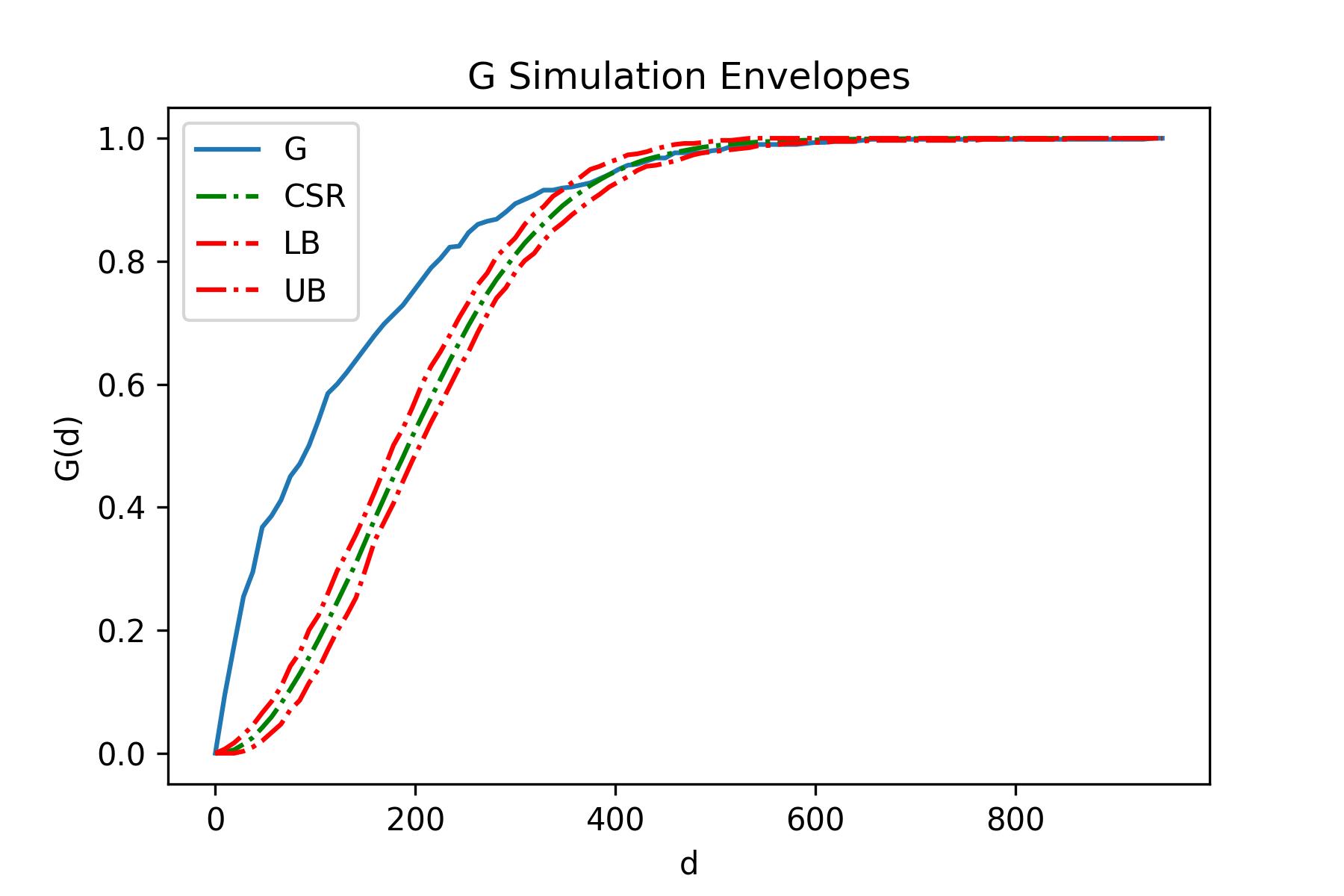

Supplement: Supplementary file 1 [file ijms-23-10435-s001.zip › supplementary File S1/STORM FILTERED G/storm_btx_0_filtered.jpg]

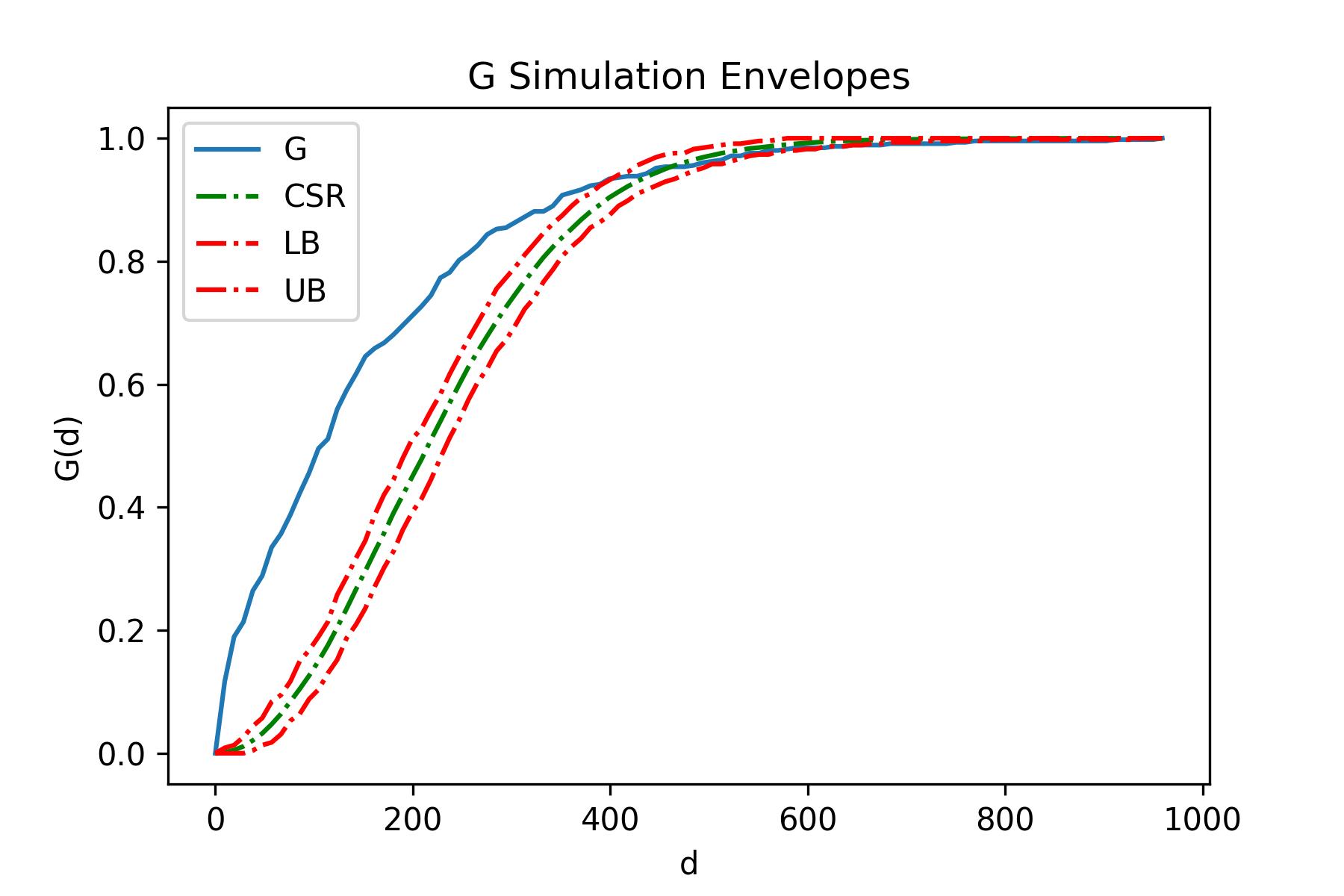

Supplement: Supplementary file 1 [file ijms-23-10435-s001.zip › supplementary File S1/STORM FILTERED G/storm_btx_1_filtered.jpg]

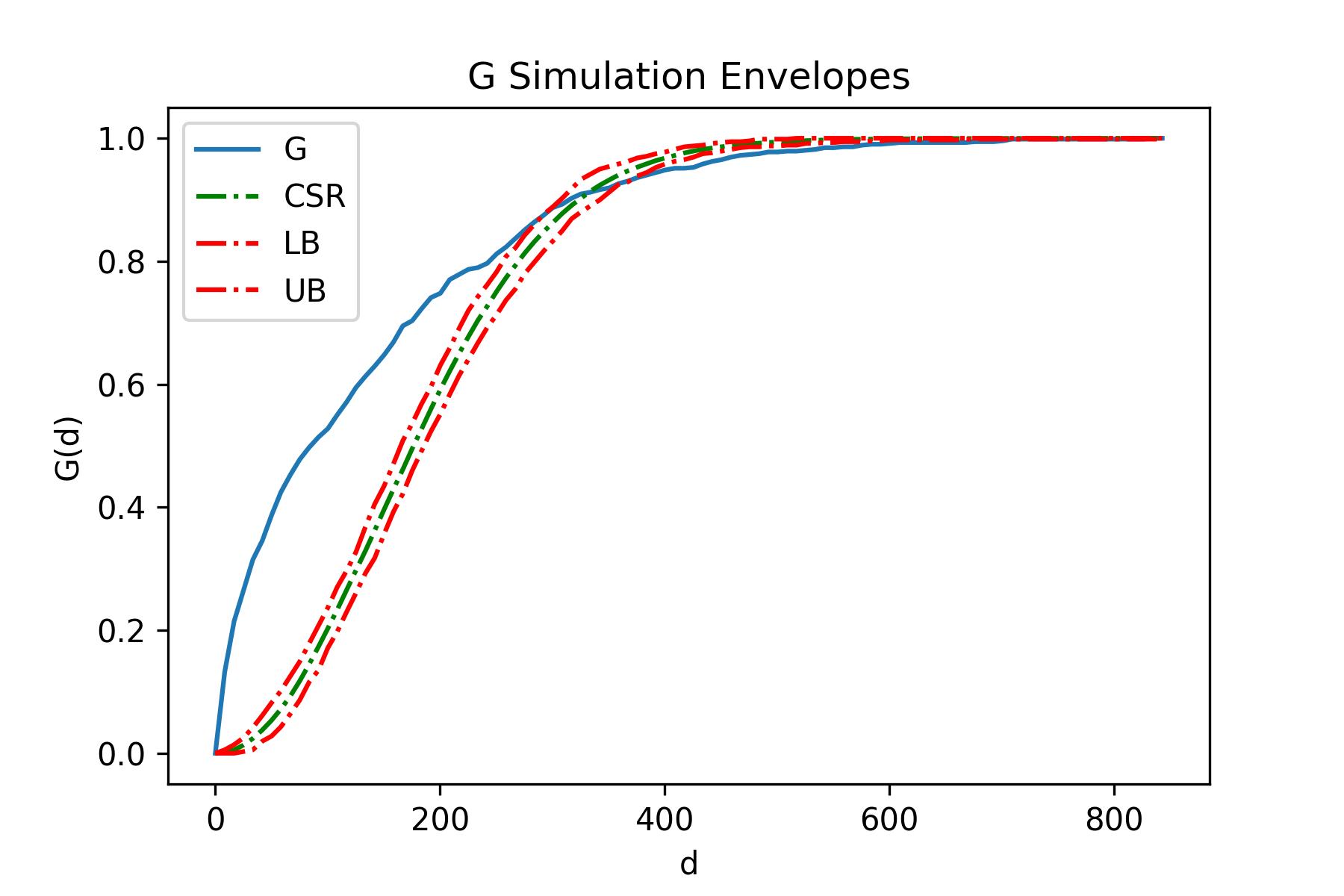

Supplement: Supplementary file 1 [file ijms-23-10435-s001.zip › supplementary File S1/STORM FILTERED G/storm_btx_2_filtered.jpg]

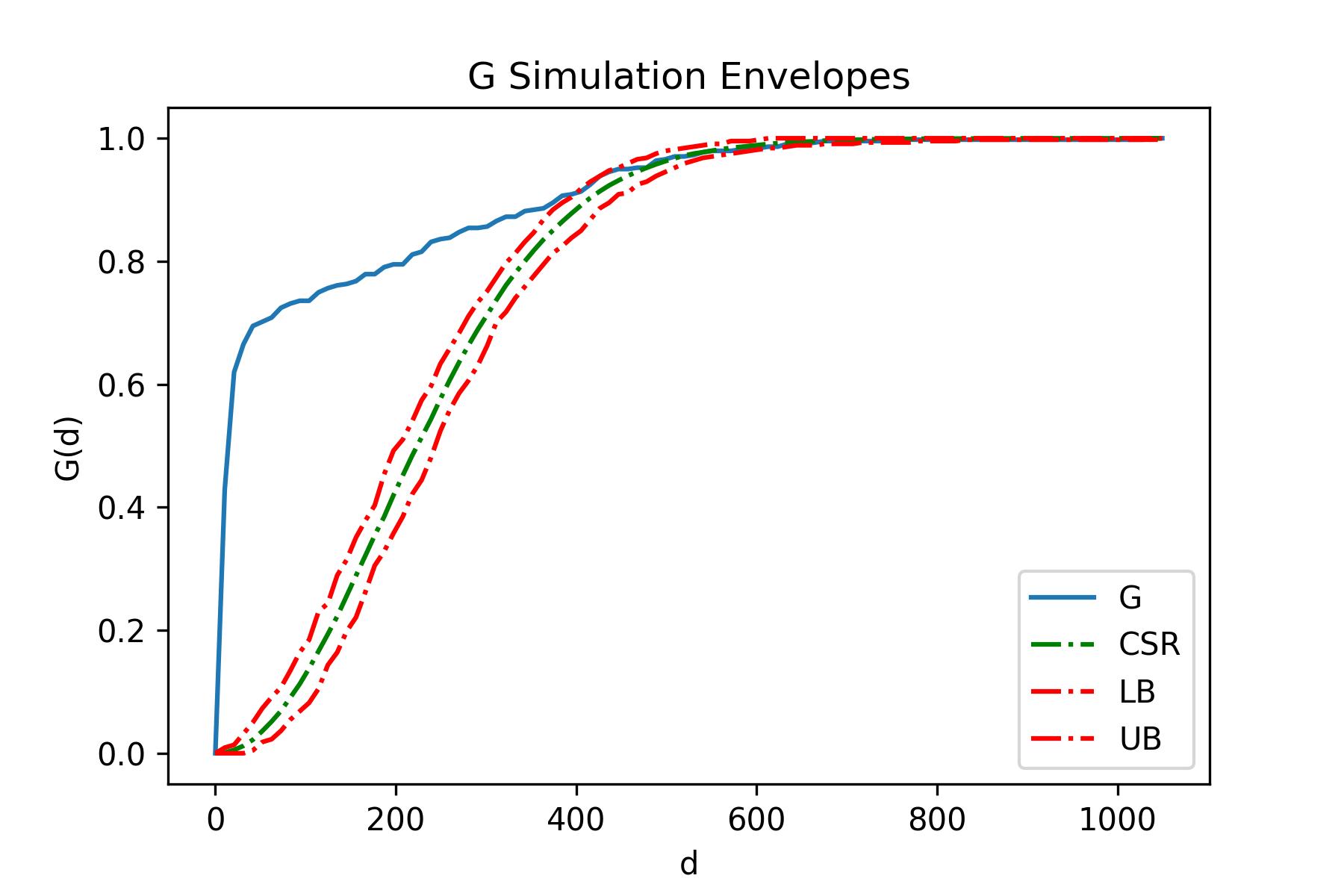

Supplement: Supplementary file 1 [file ijms-23-10435-s001.zip › supplementary File S1/STORM FILTERED G/storm_mab_0_filtered.jpg]

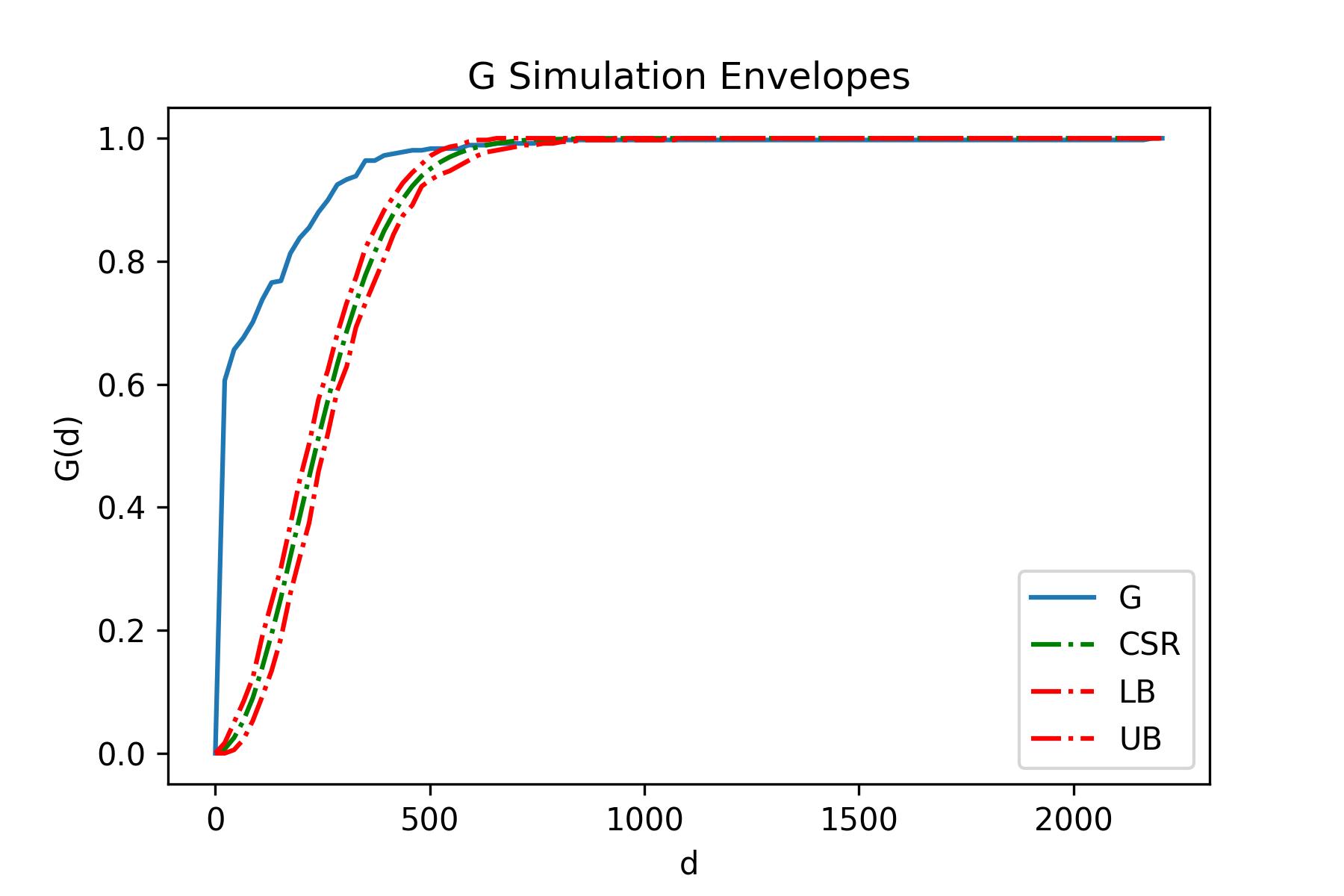

Supplement: Supplementary file 1 [file ijms-23-10435-s001.zip › supplementary File S1/STORM FILTERED G/storm_mab_1_filtered.jpg]

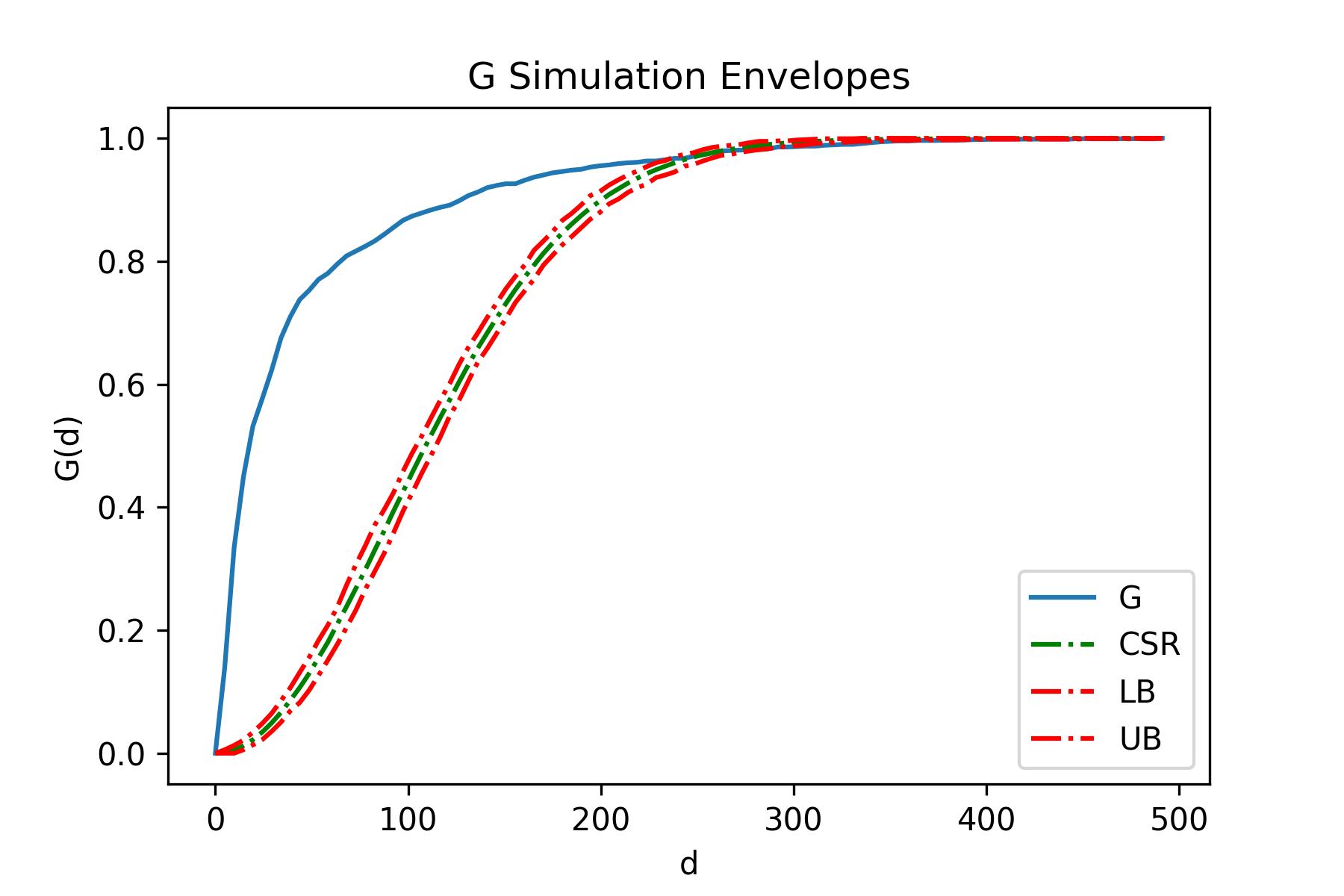

Supplement: Supplementary file 1 [file ijms-23-10435-s001.zip › supplementary File S1/STORM FILTERED G/storm_mab_2_filtered.jpg]

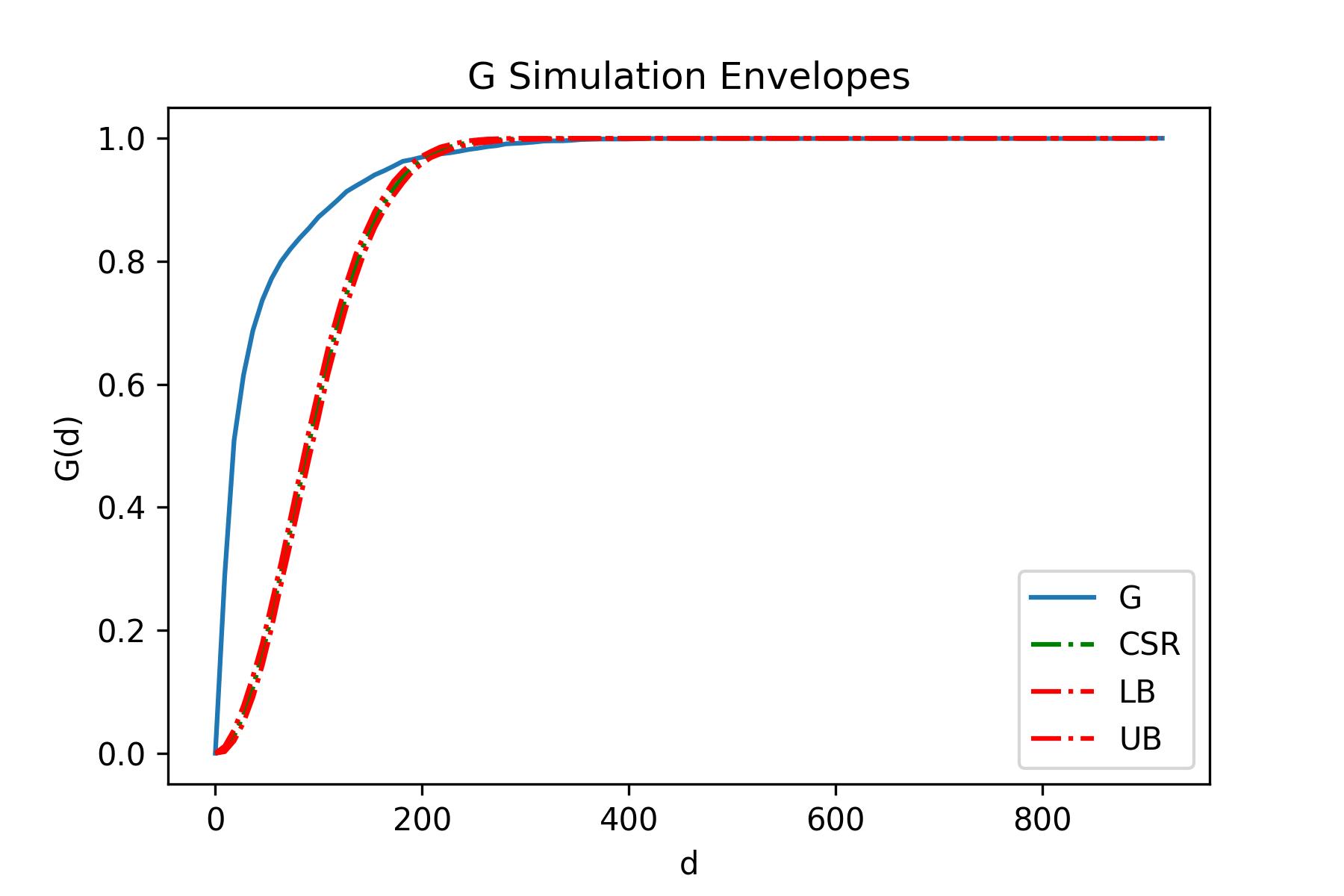

Supplement: Supplementary file 1 [file ijms-23-10435-s001.zip › supplementary File S1/STORM FILTERED G/storm_mab_3_filtered.jpg]

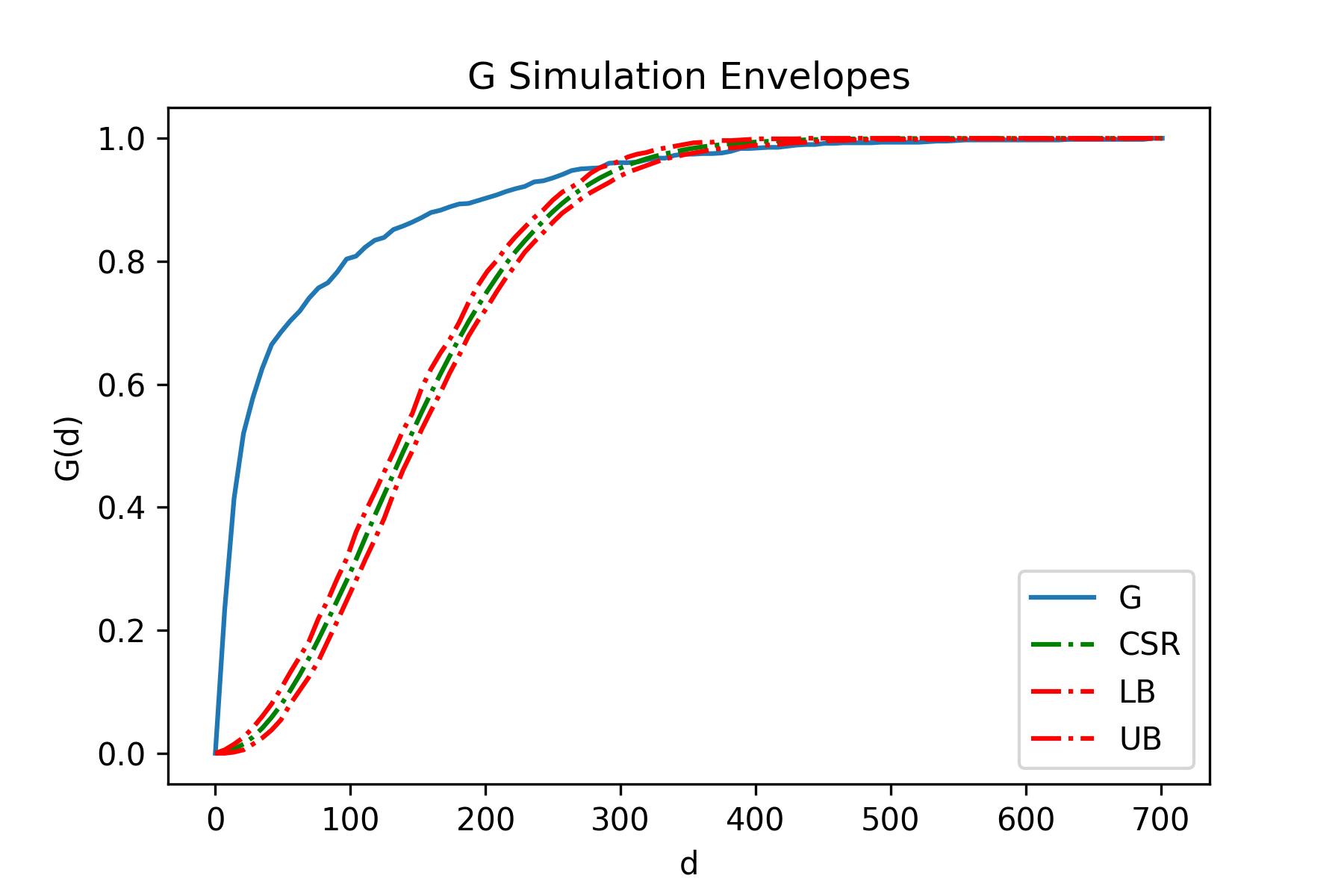

Supplement: Supplementary file 1 [file ijms-23-10435-s001.zip › supplementary File S1/STORM FILTERED G/storm_mab_4_filtered.jpg]

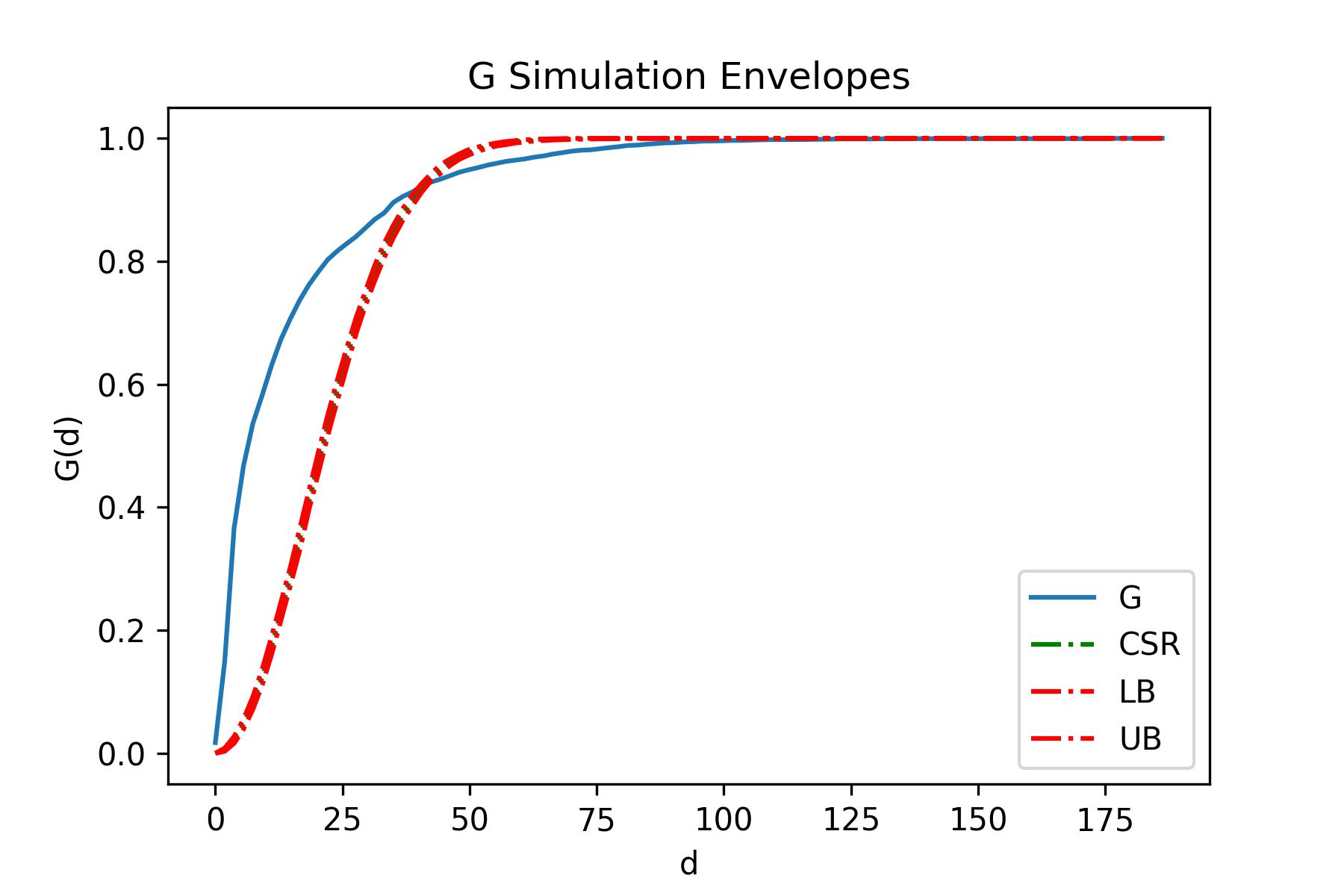

Supplement: Supplementary file 1 [file ijms-23-10435-s001.zip › supplementary File S1/STORM G/storm_btx_0.jpg]

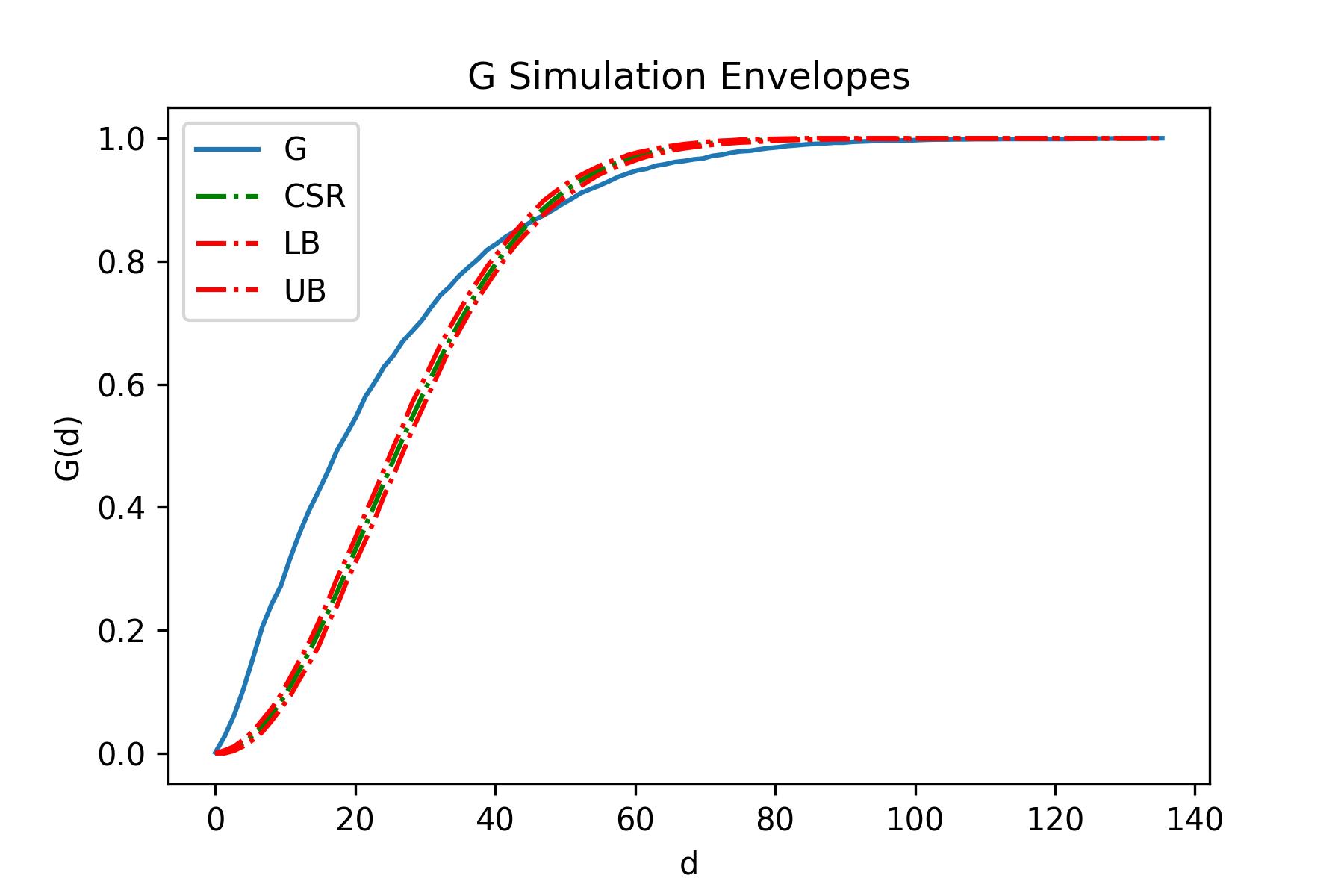

Supplement: Supplementary file 1 [file ijms-23-10435-s001.zip › supplementary File S1/STORM G/storm_btx_1.jpg]

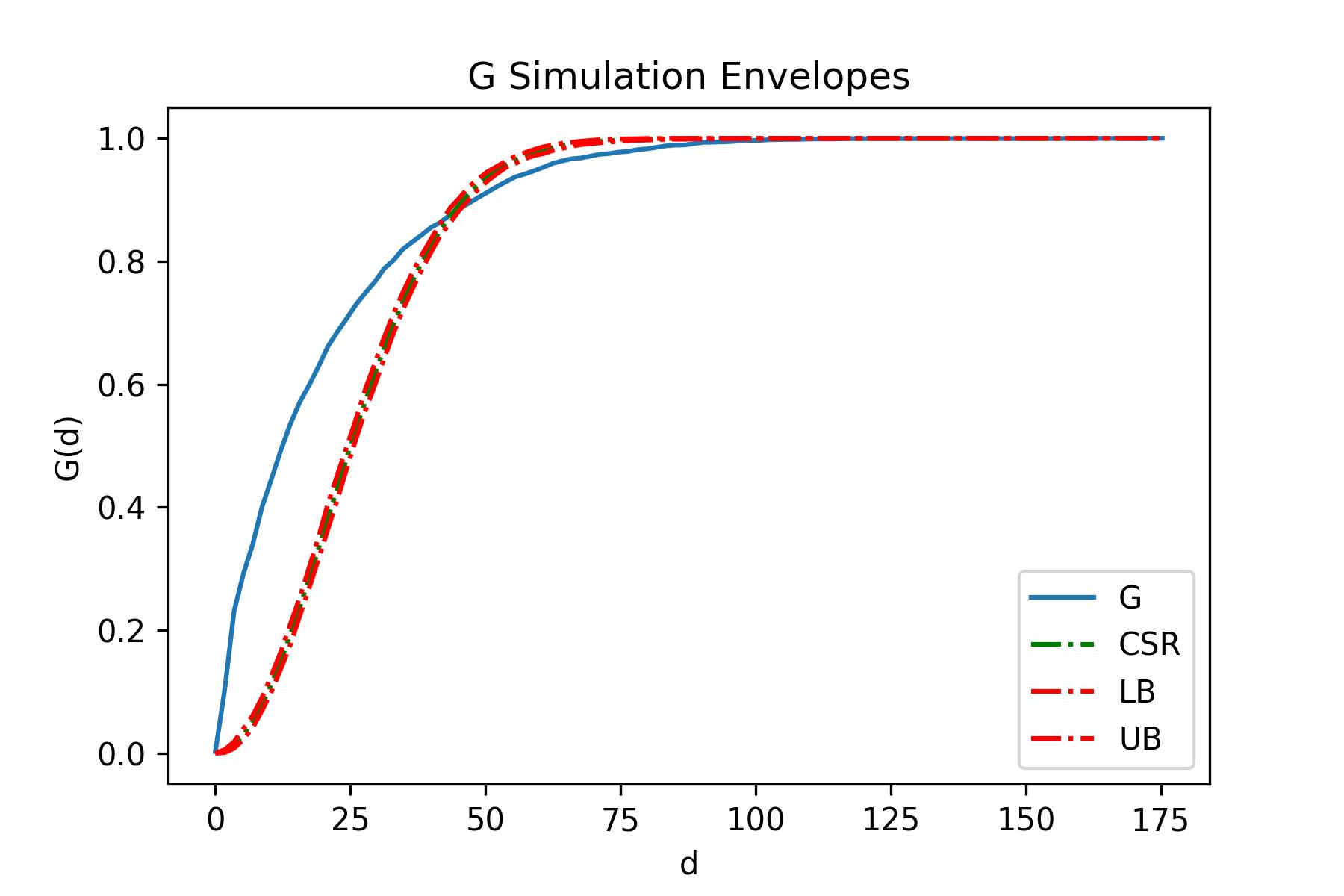

Supplement: Supplementary file 1 [file ijms-23-10435-s001.zip › supplementary File S1/STORM G/storm_btx_10.jpg]

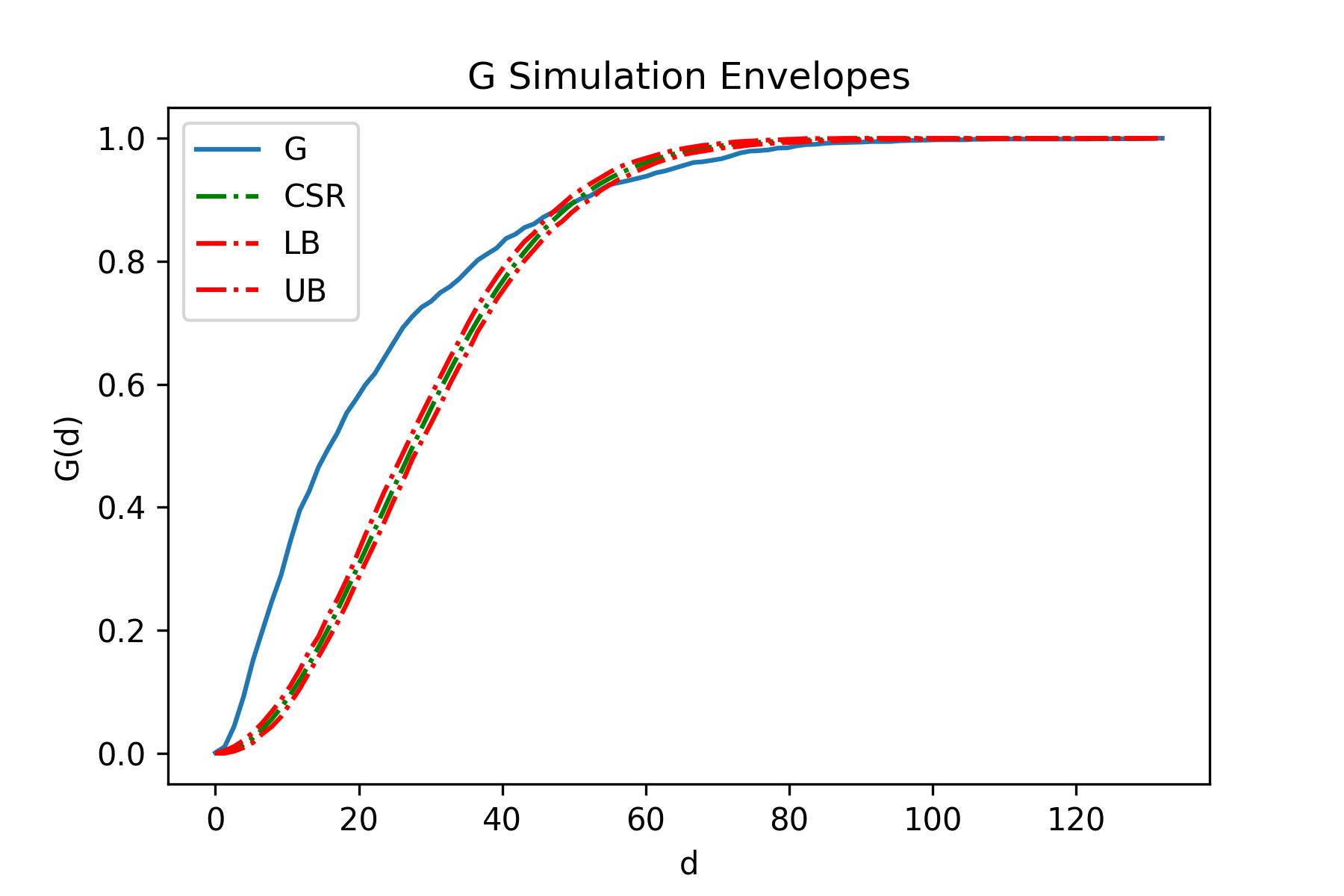

Supplement: Supplementary file 1 [file ijms-23-10435-s001.zip › supplementary File S1/STORM G/storm_btx_11.jpg]

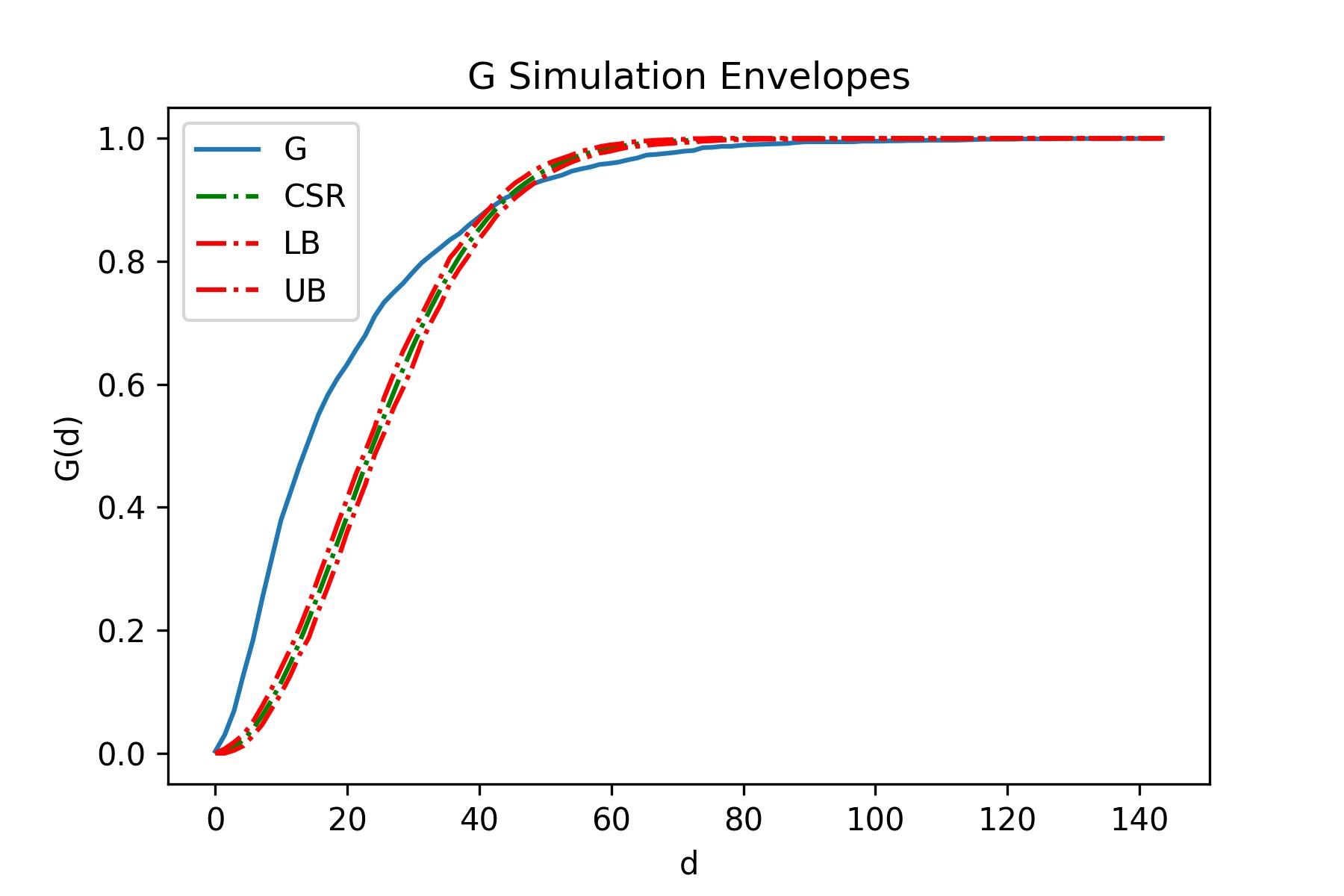

Supplement: Supplementary file 1 [file ijms-23-10435-s001.zip › supplementary File S1/STORM G/storm_btx_12.jpg]

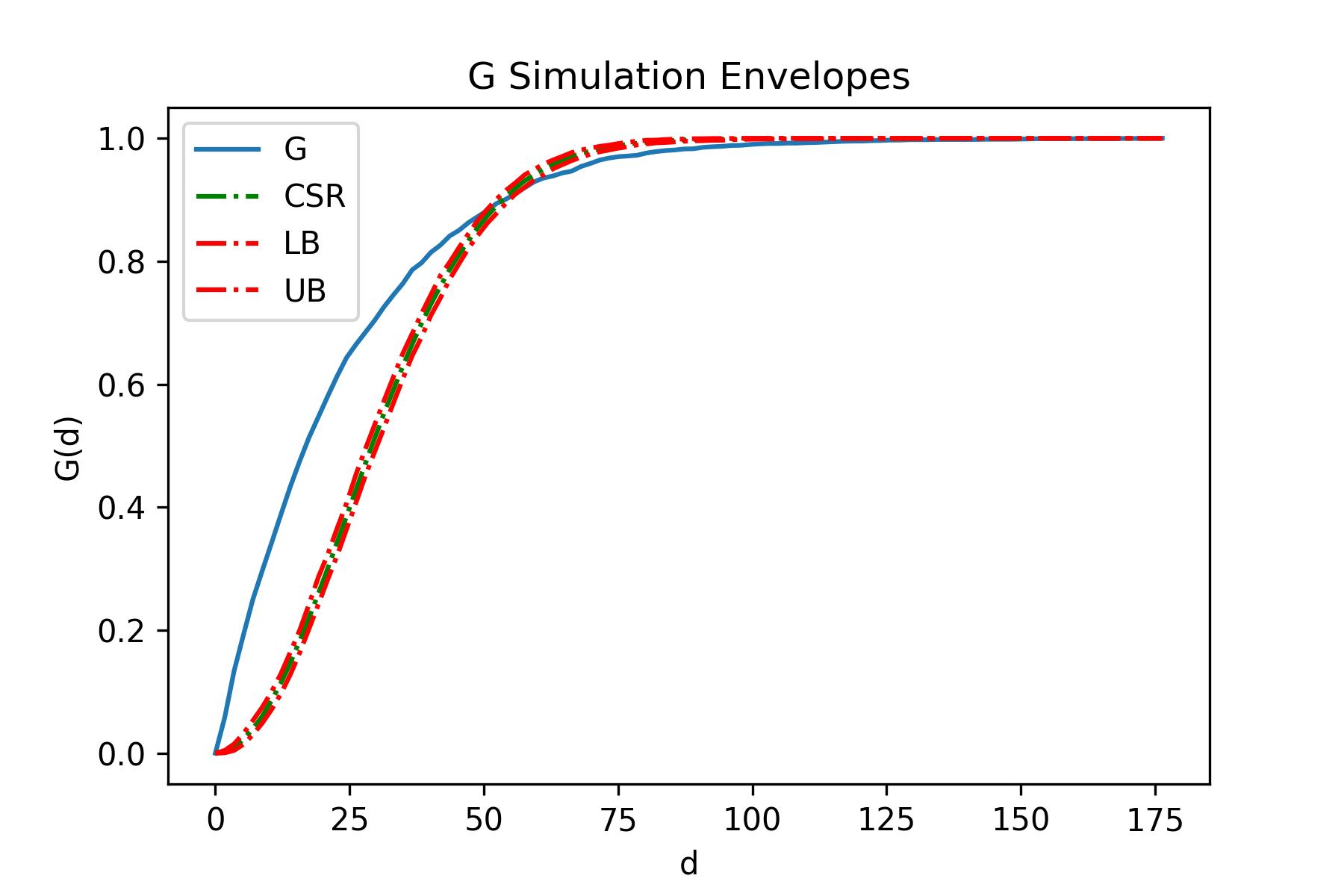

Supplement: Supplementary file 1 [file ijms-23-10435-s001.zip › supplementary File S1/STORM G/storm_btx_13.jpg]

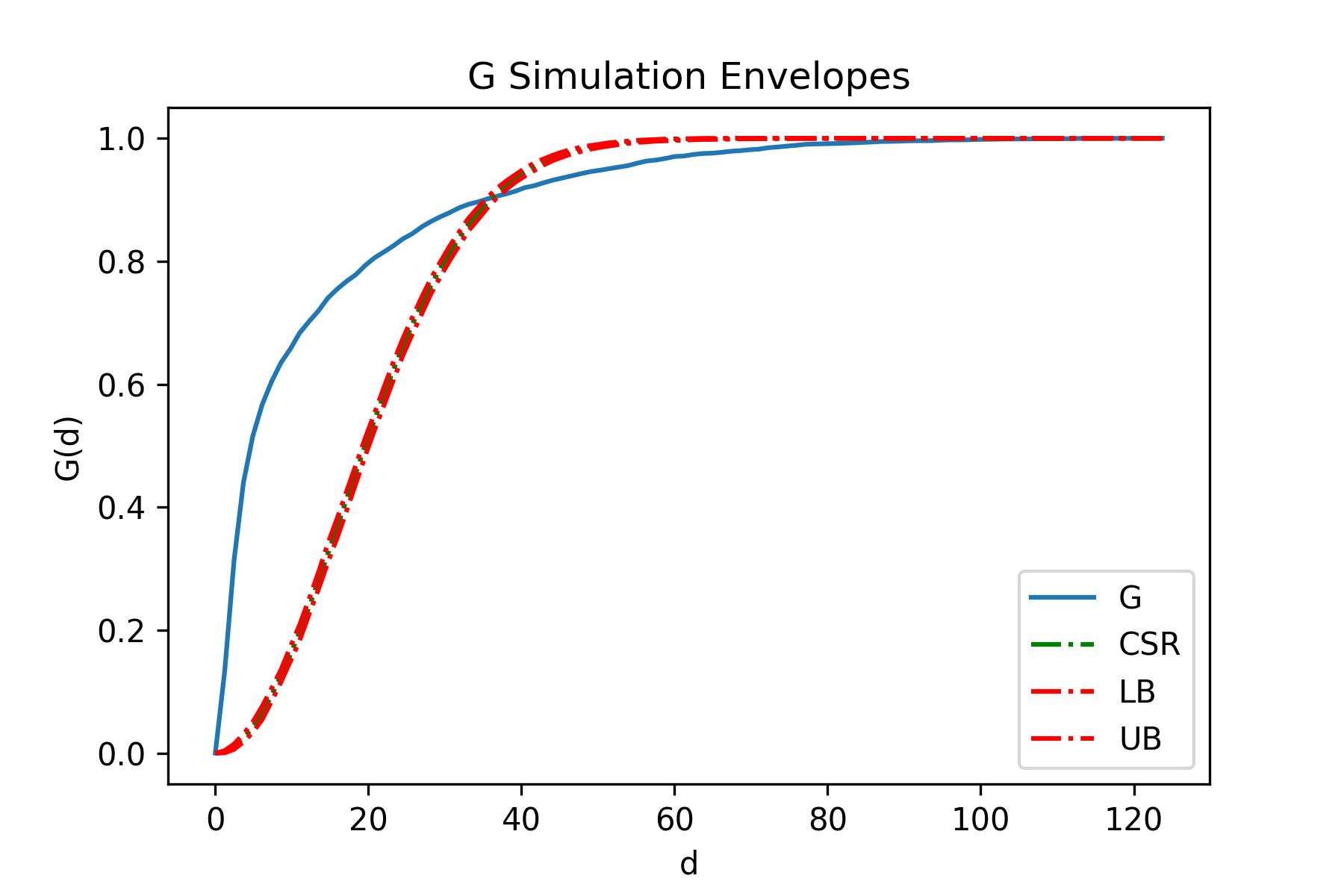

Supplement: Supplementary file 1 [file ijms-23-10435-s001.zip › supplementary File S1/STORM G/storm_btx_14.jpg]

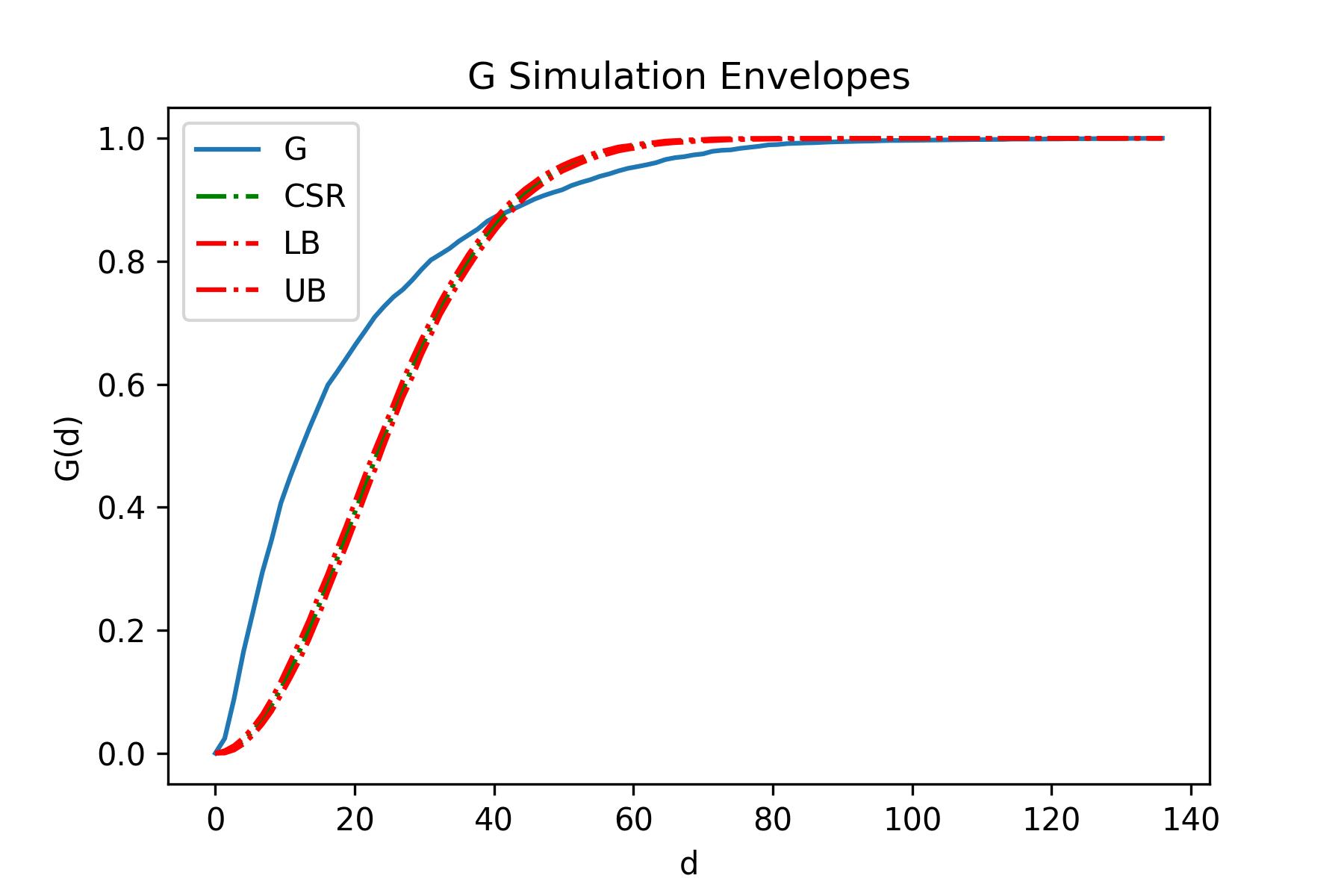

Supplement: Supplementary file 1 [file ijms-23-10435-s001.zip › supplementary File S1/STORM G/storm_btx_15.jpg]

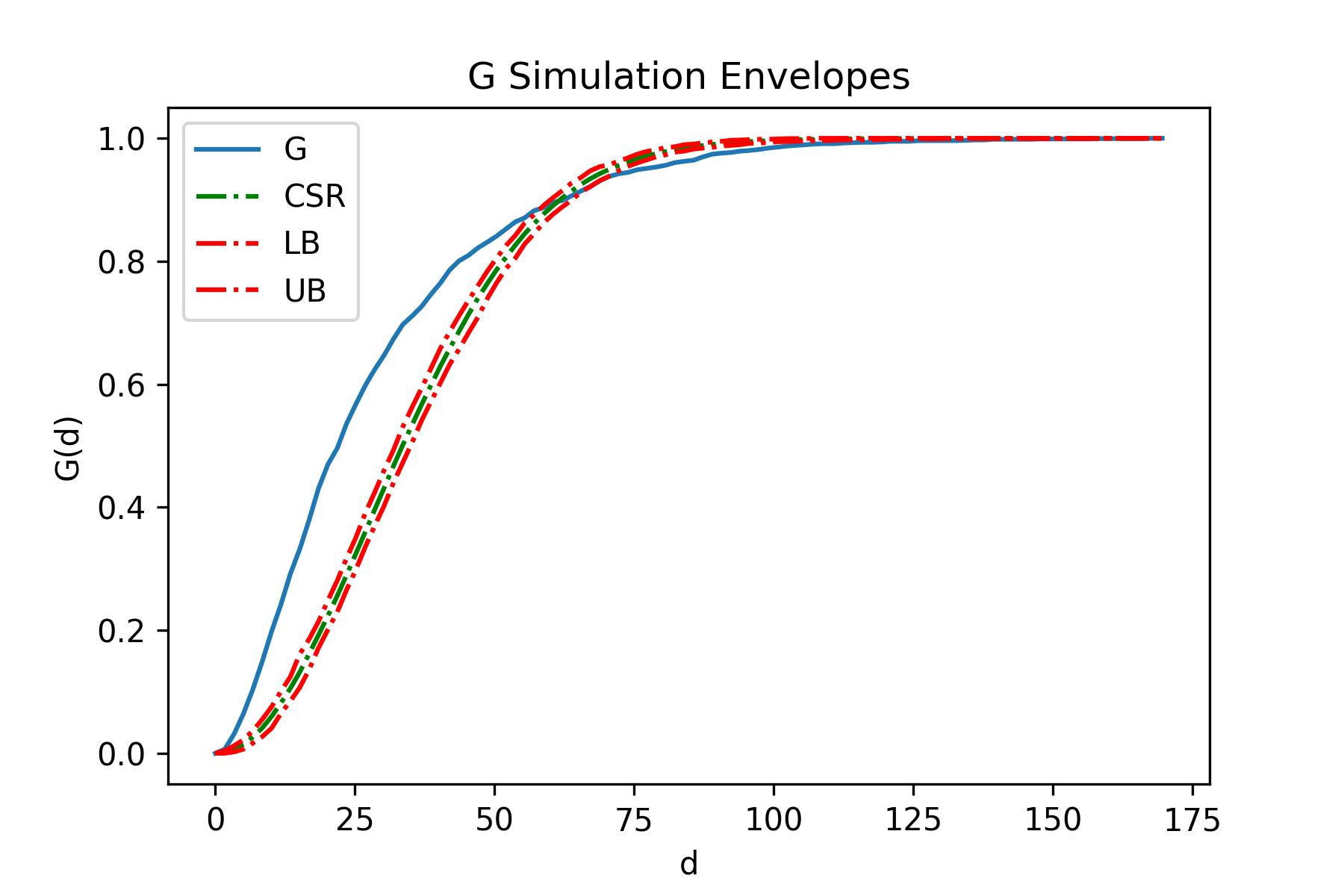

Supplement: Supplementary file 1 [file ijms-23-10435-s001.zip › supplementary File S1/STORM G/storm_btx_16.jpg]

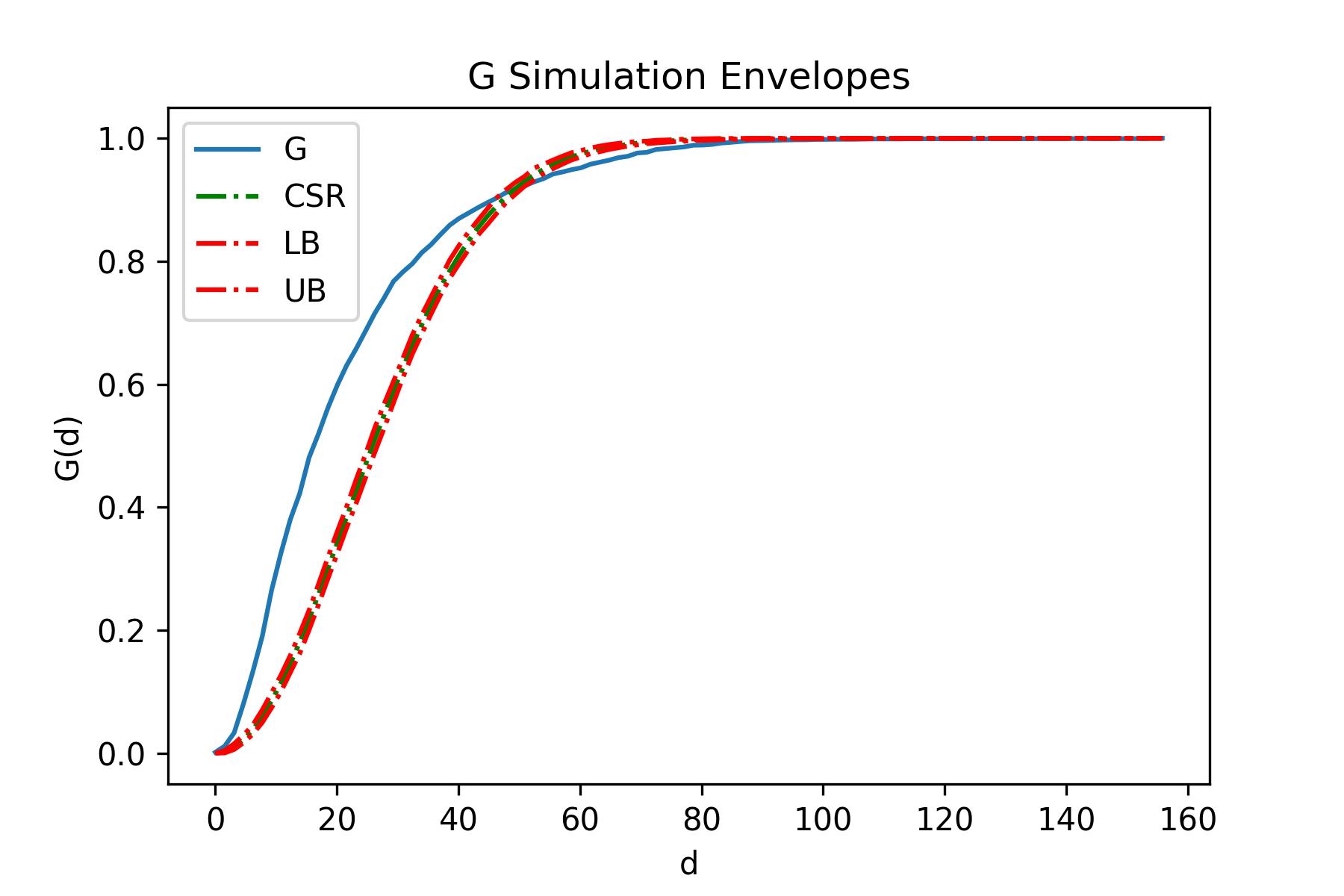

Supplement: Supplementary file 1 [file ijms-23-10435-s001.zip › supplementary File S1/STORM G/storm_btx_17.jpg]

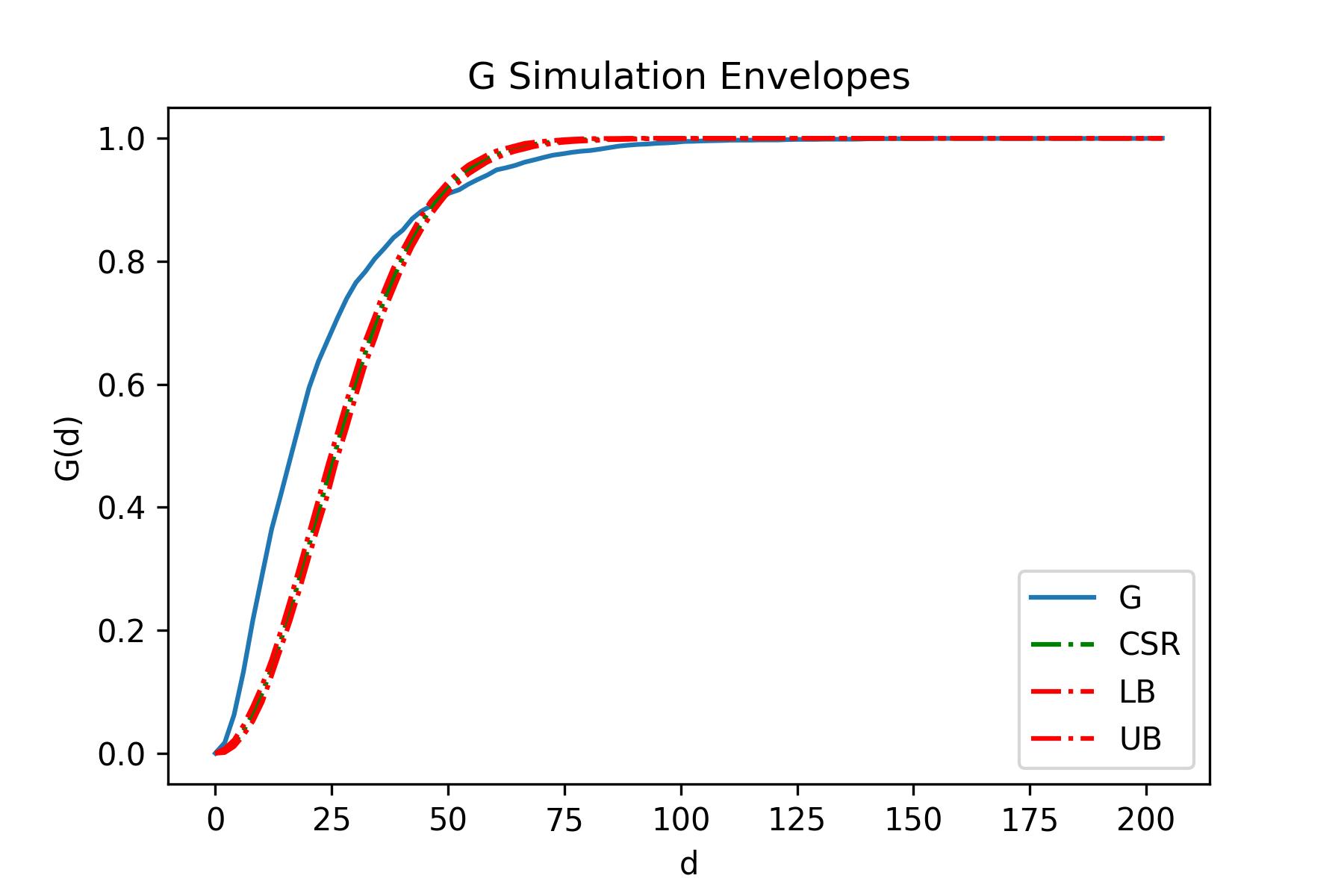

Supplement: Supplementary file 1 [file ijms-23-10435-s001.zip › supplementary File S1/STORM G/storm_btx_18.jpg]

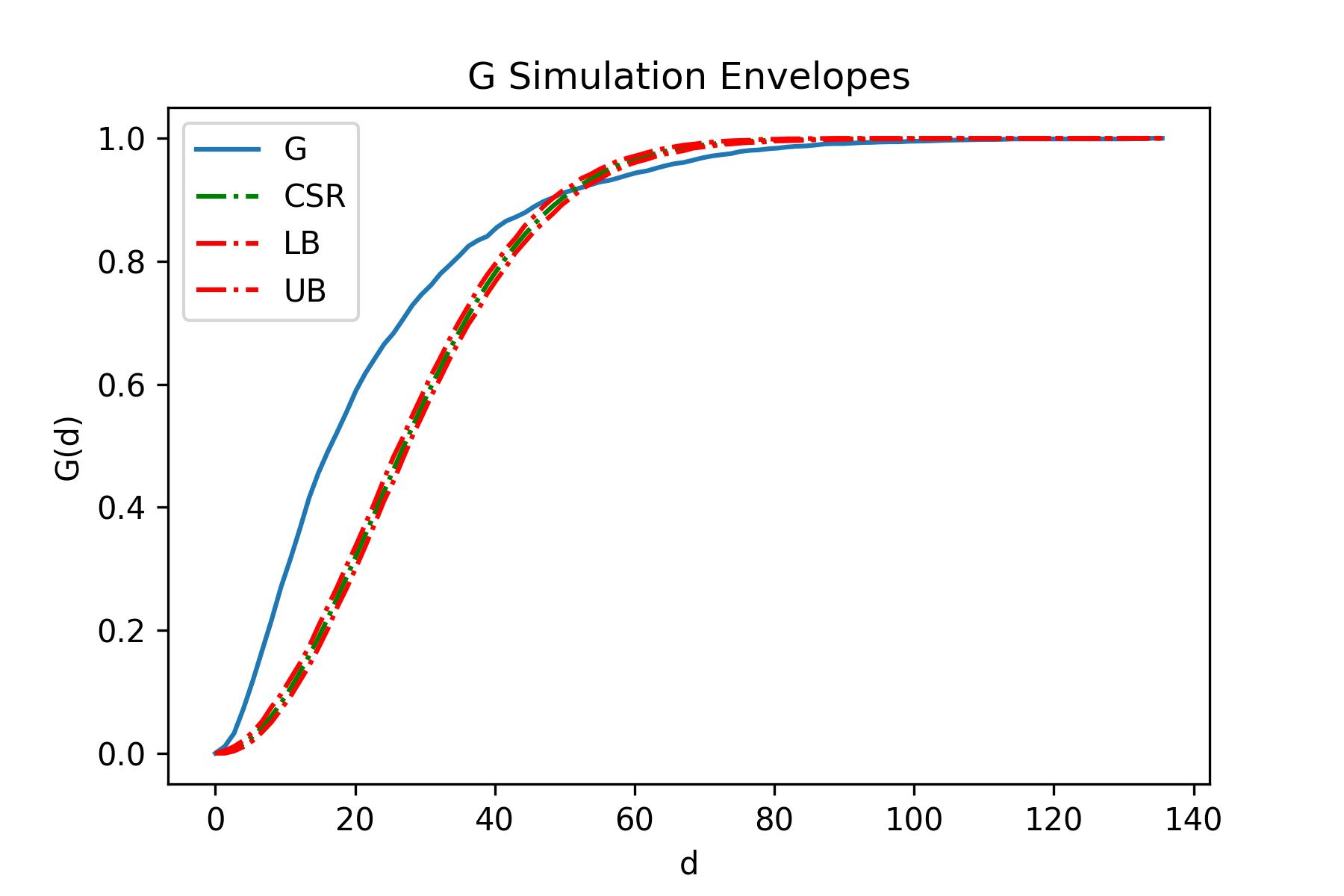

Supplement: Supplementary file 1 [file ijms-23-10435-s001.zip › supplementary File S1/STORM G/storm_btx_19.jpg]

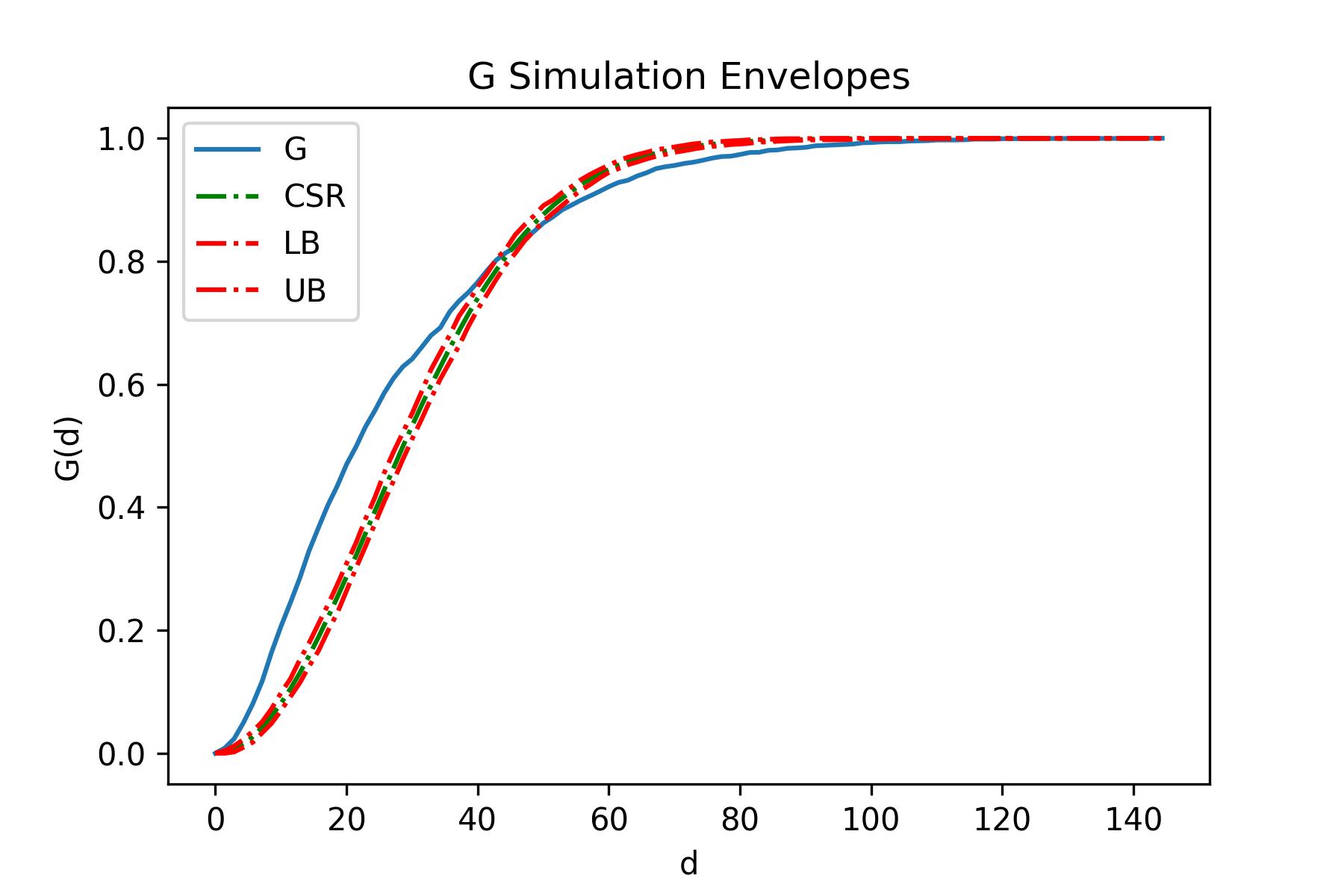

Supplement: Supplementary file 1 [file ijms-23-10435-s001.zip › supplementary File S1/STORM G/storm_btx_2.jpg]

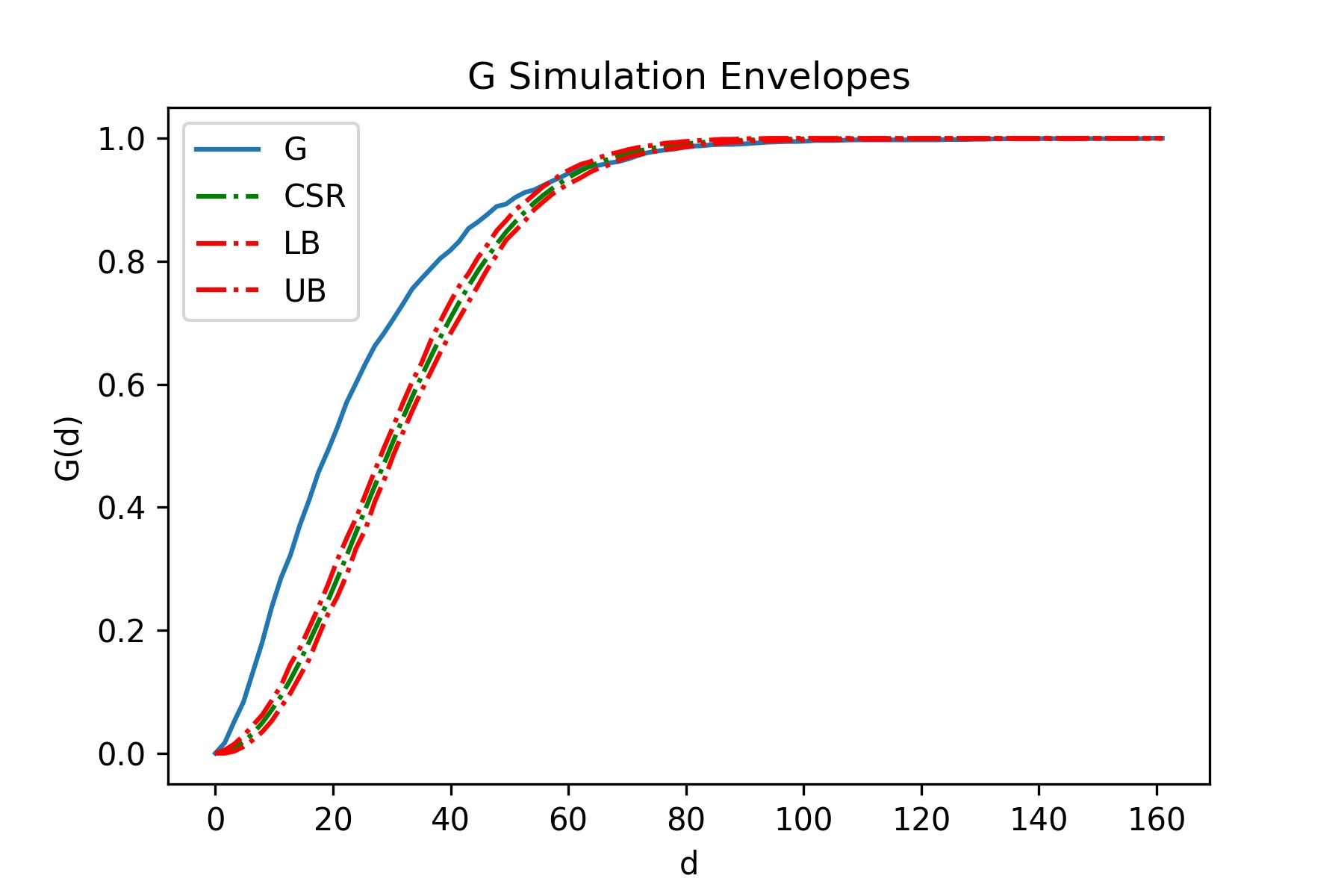

Supplement: Supplementary file 1 [file ijms-23-10435-s001.zip › supplementary File S1/STORM G/storm_btx_20.jpg]

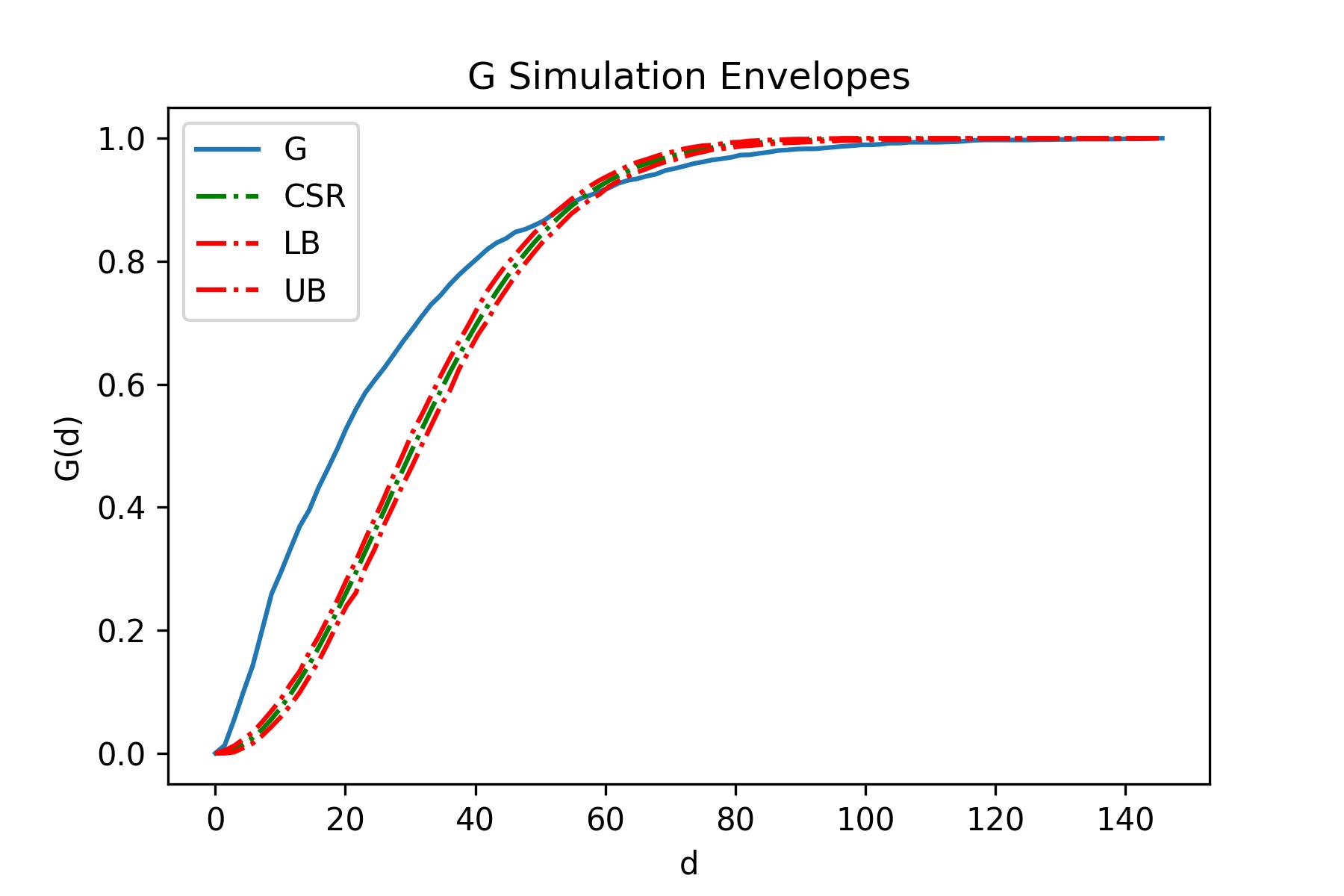

Supplement: Supplementary file 1 [file ijms-23-10435-s001.zip › supplementary File S1/STORM G/storm_btx_21.jpg]

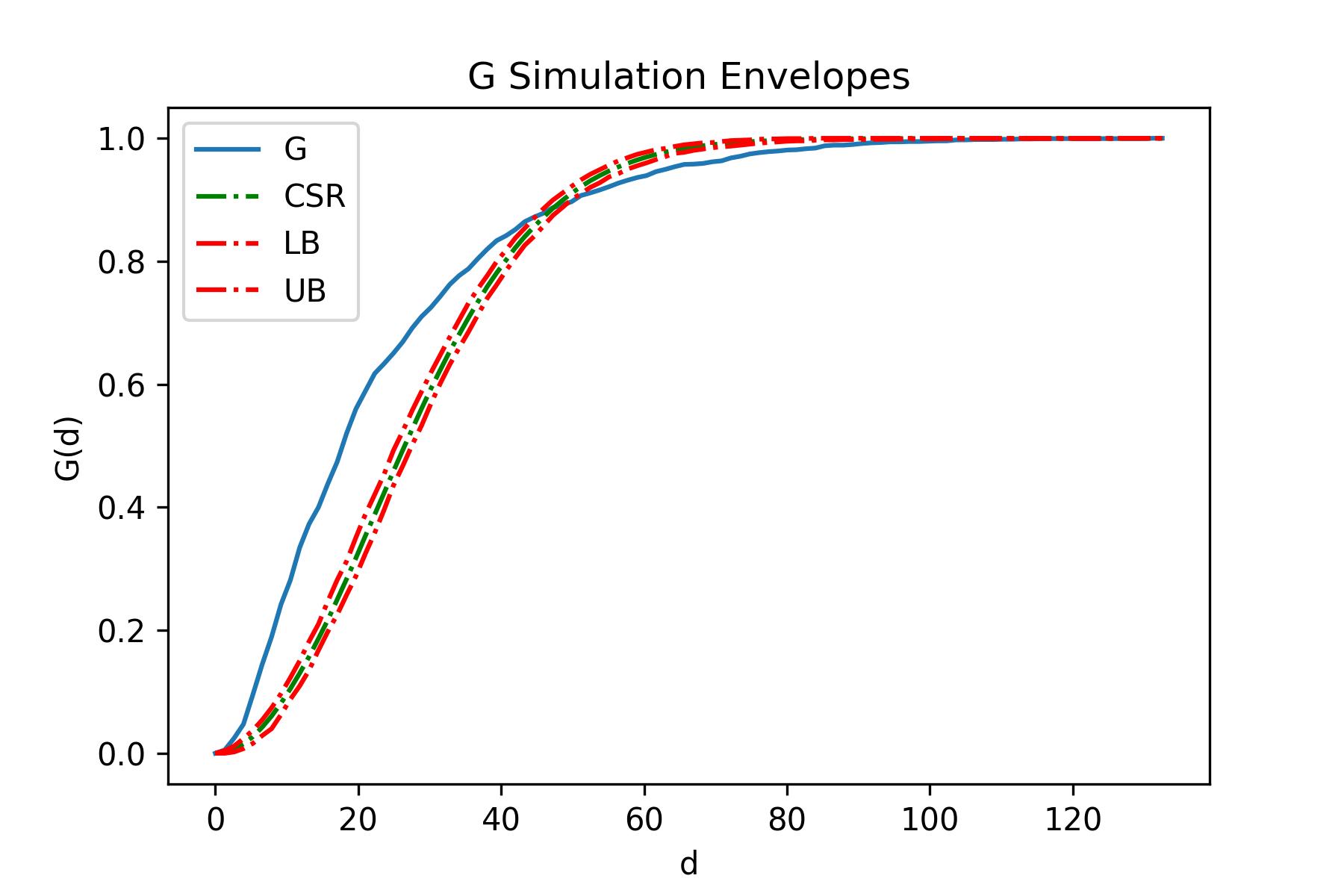

Supplement: Supplementary file 1 [file ijms-23-10435-s001.zip › supplementary File S1/STORM G/storm_btx_22.jpg]

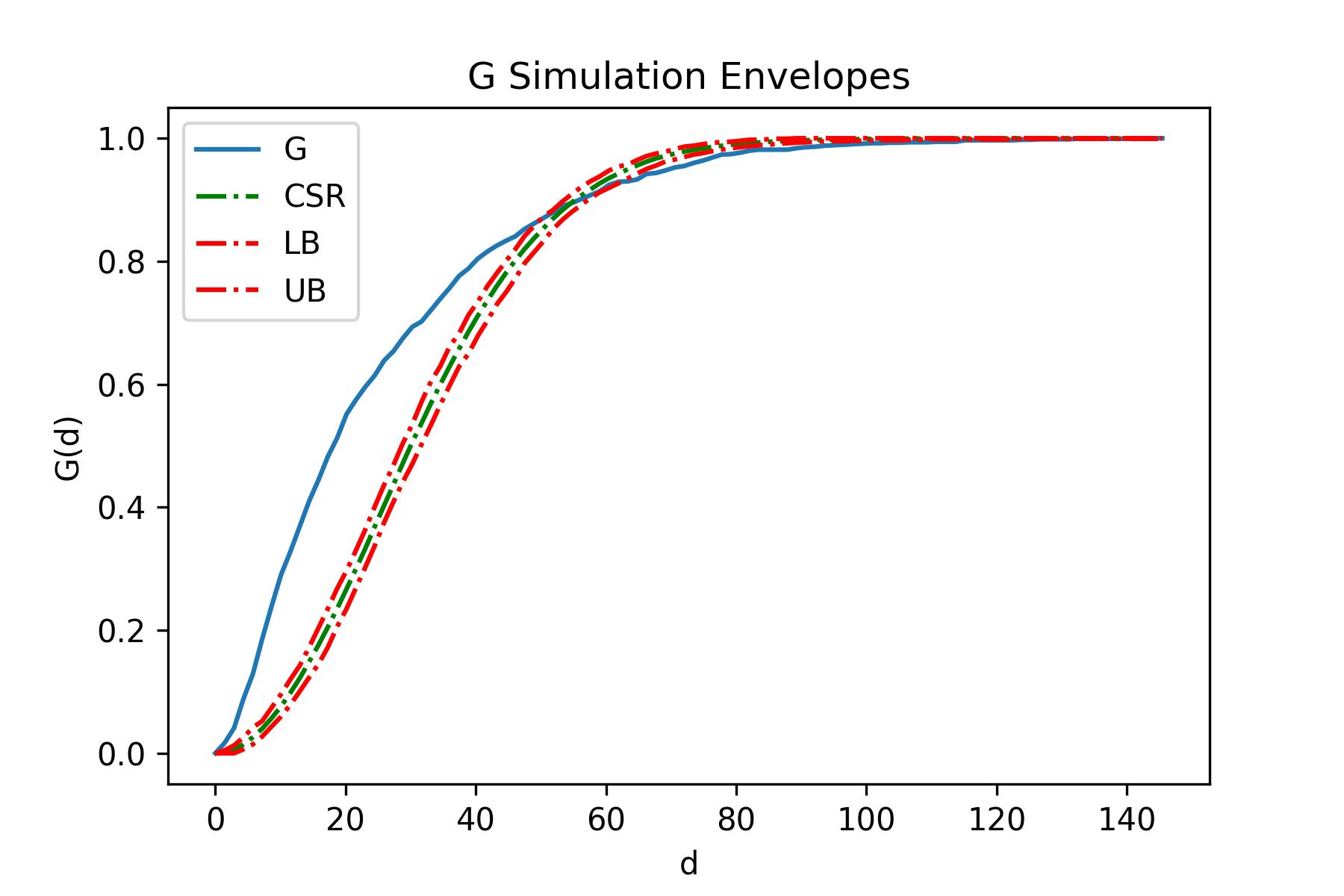

Supplement: Supplementary file 1 [file ijms-23-10435-s001.zip › supplementary File S1/STORM G/storm_btx_23.jpg]

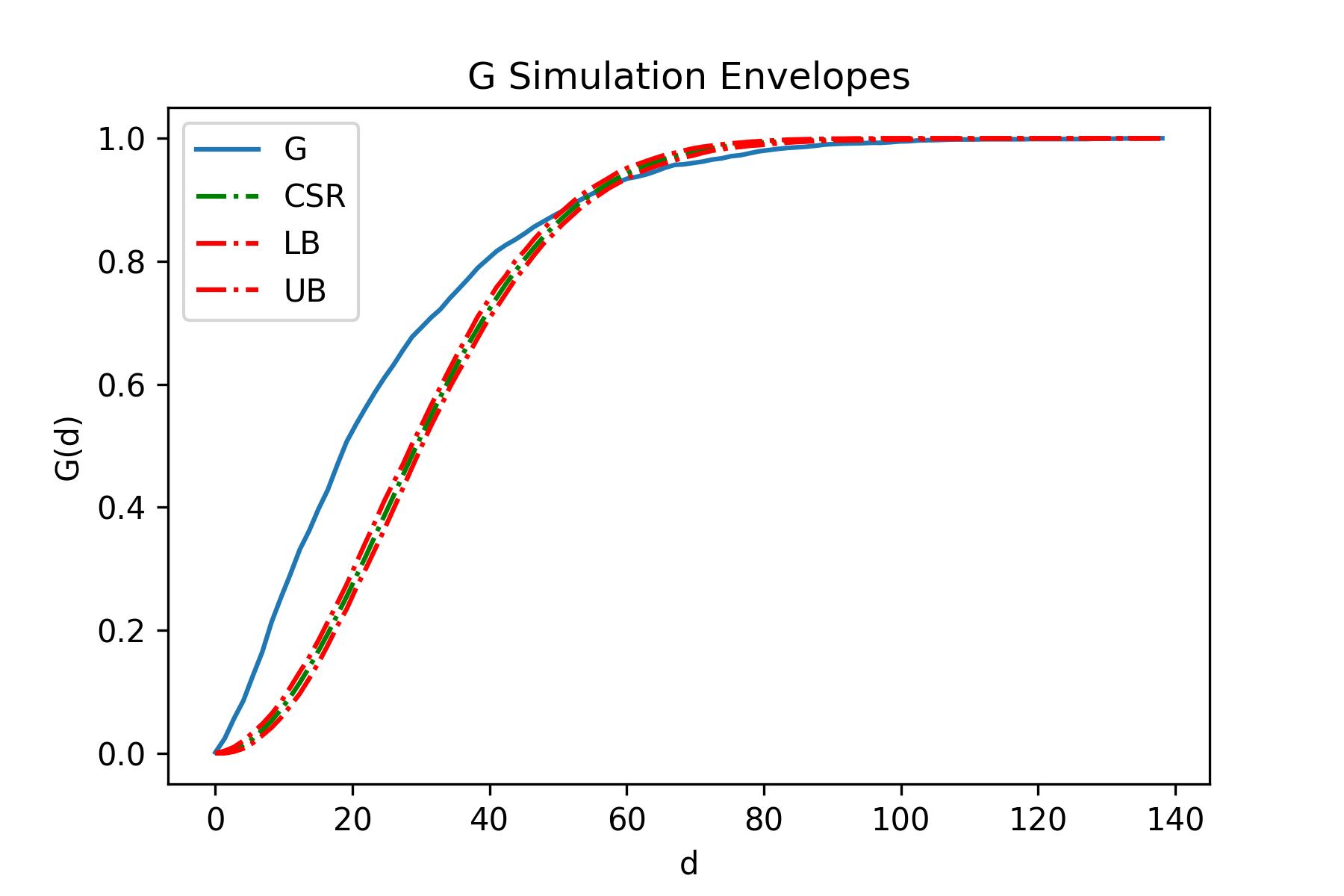

Supplement: Supplementary file 1 [file ijms-23-10435-s001.zip › supplementary File S1/STORM G/storm_btx_24.jpg]

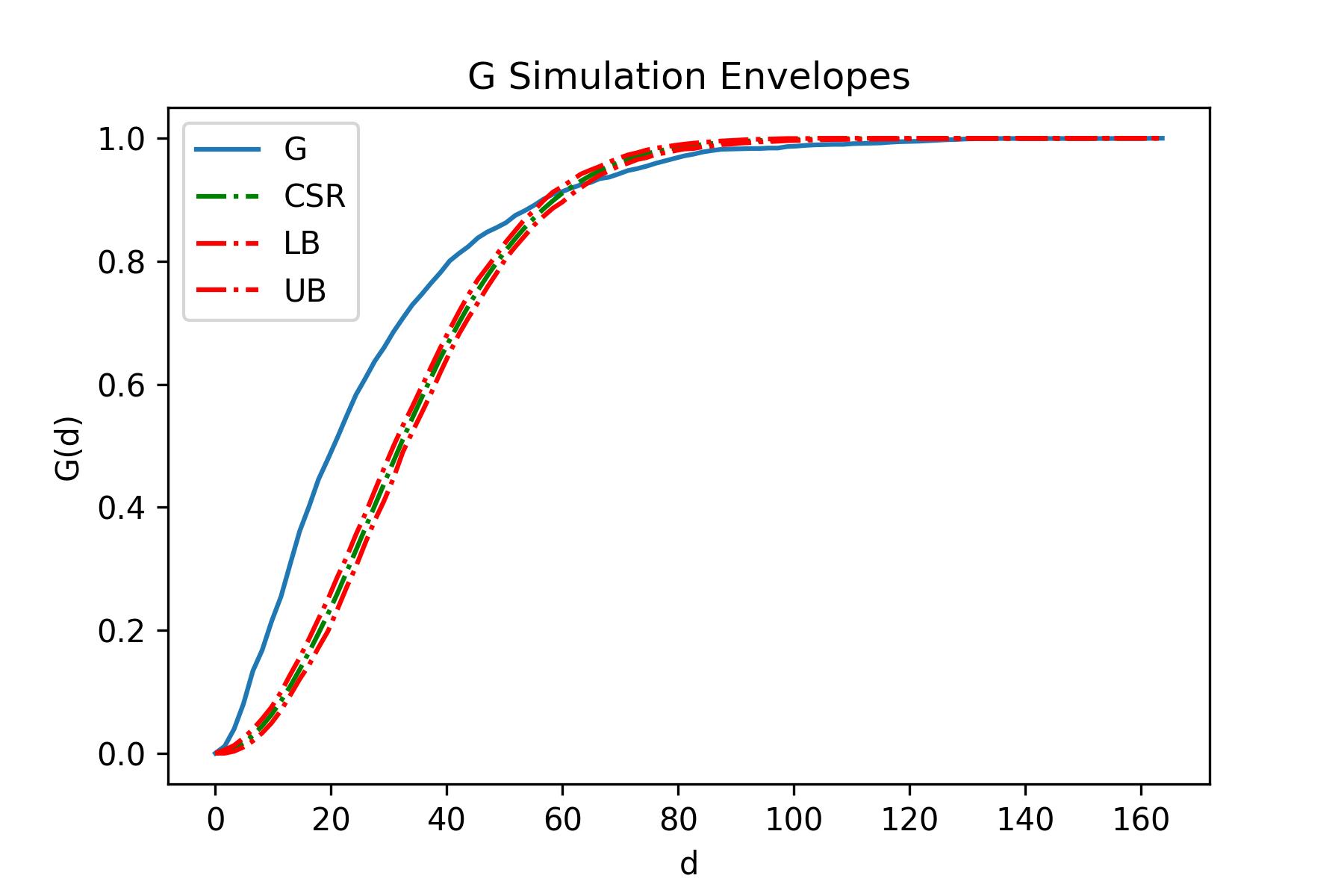

Supplement: Supplementary file 1 [file ijms-23-10435-s001.zip › supplementary File S1/STORM G/storm_btx_25.jpg]

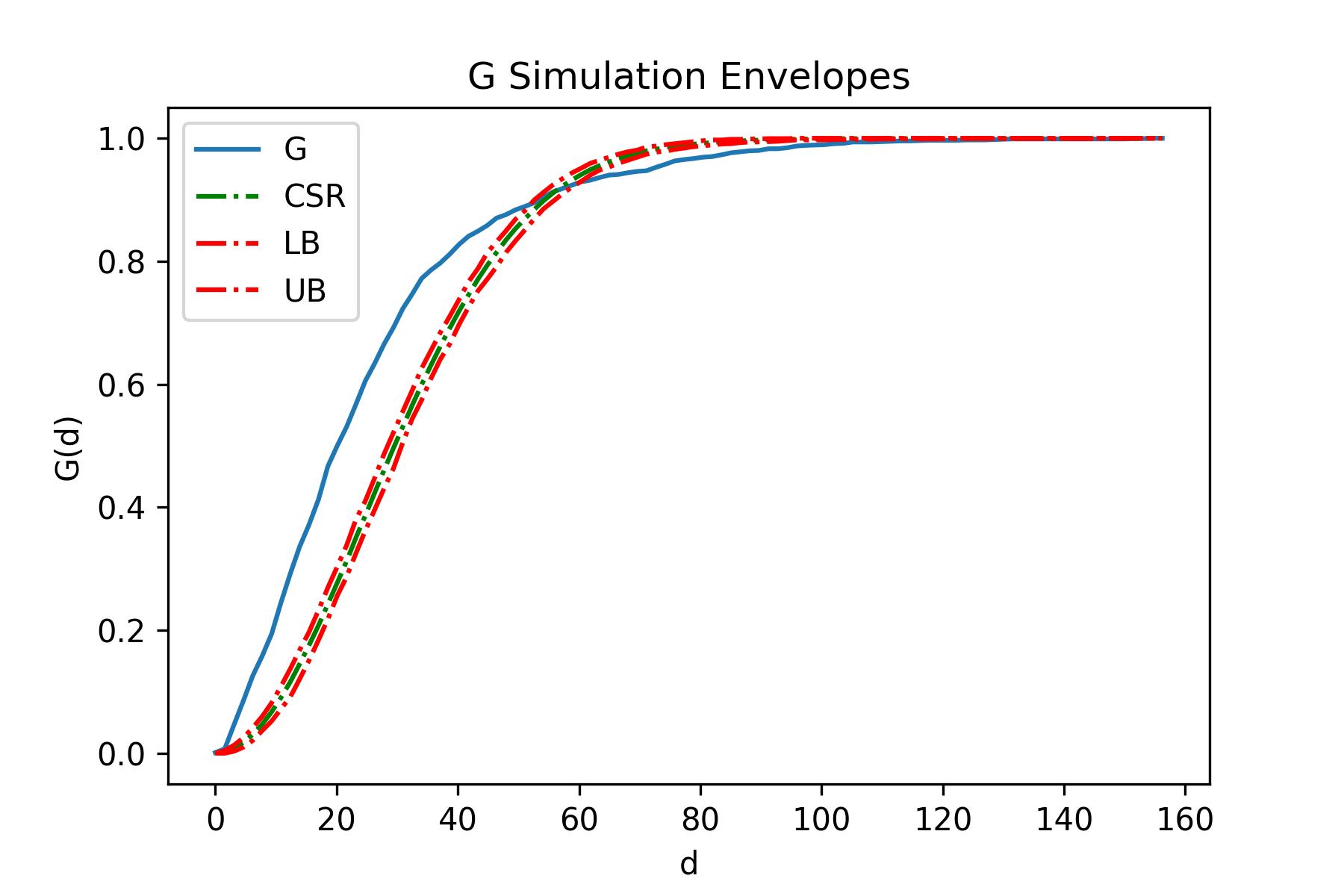

Supplement: Supplementary file 1 [file ijms-23-10435-s001.zip › supplementary File S1/STORM G/storm_btx_26.jpg]

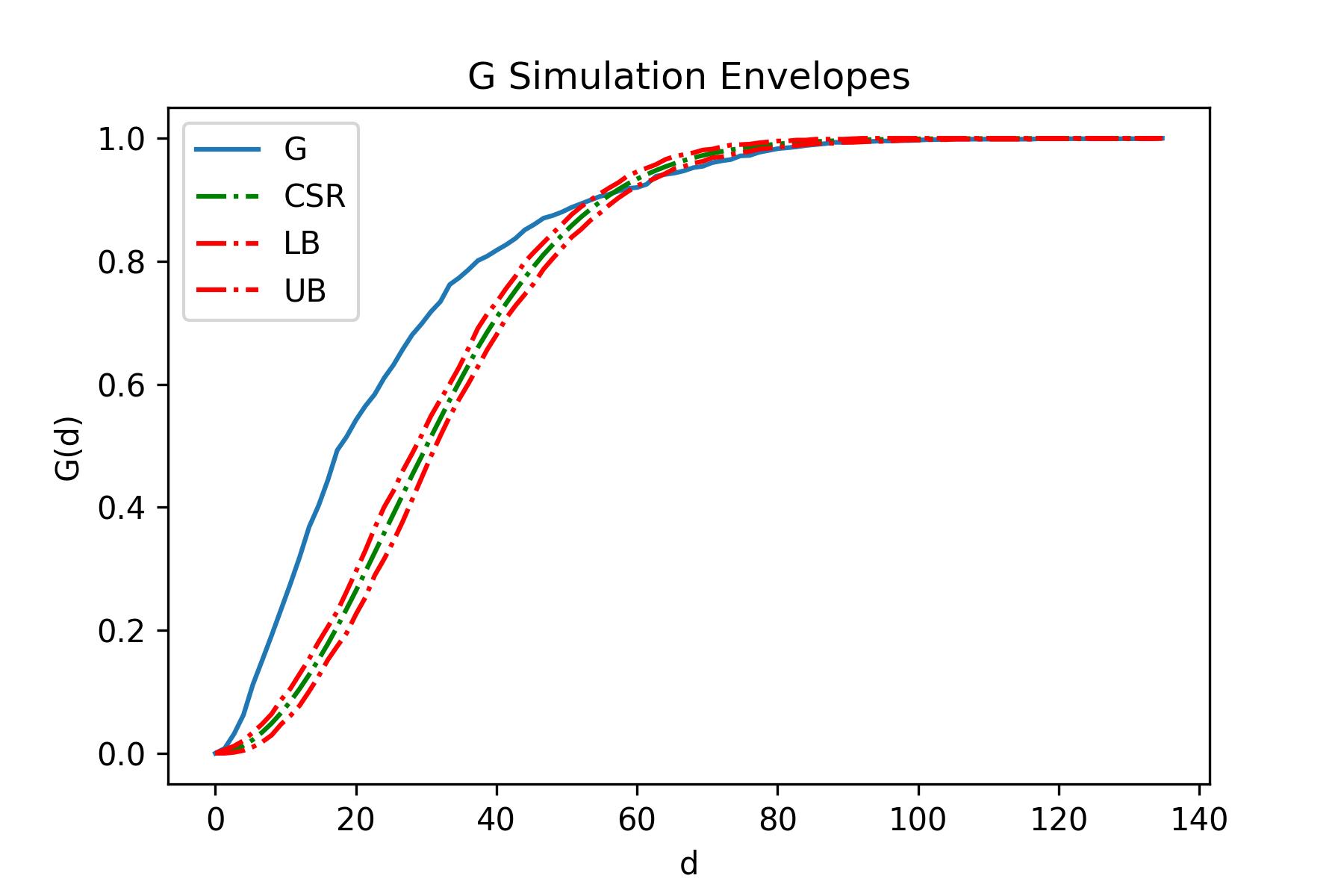

Supplement: Supplementary file 1 [file ijms-23-10435-s001.zip › supplementary File S1/STORM G/storm_btx_27.jpg]

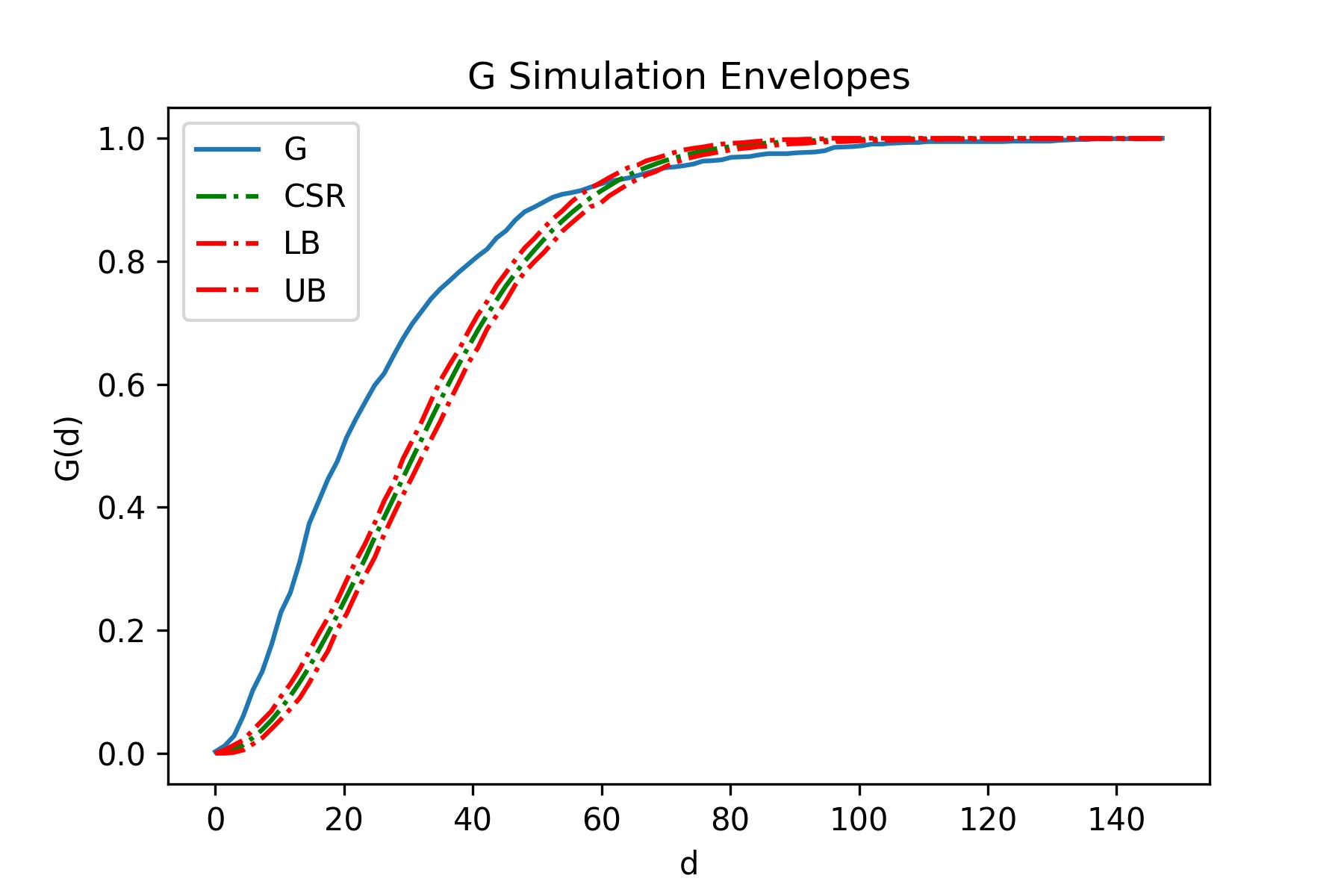

Supplement: Supplementary file 1 [file ijms-23-10435-s001.zip › supplementary File S1/STORM G/storm_btx_28.jpg]

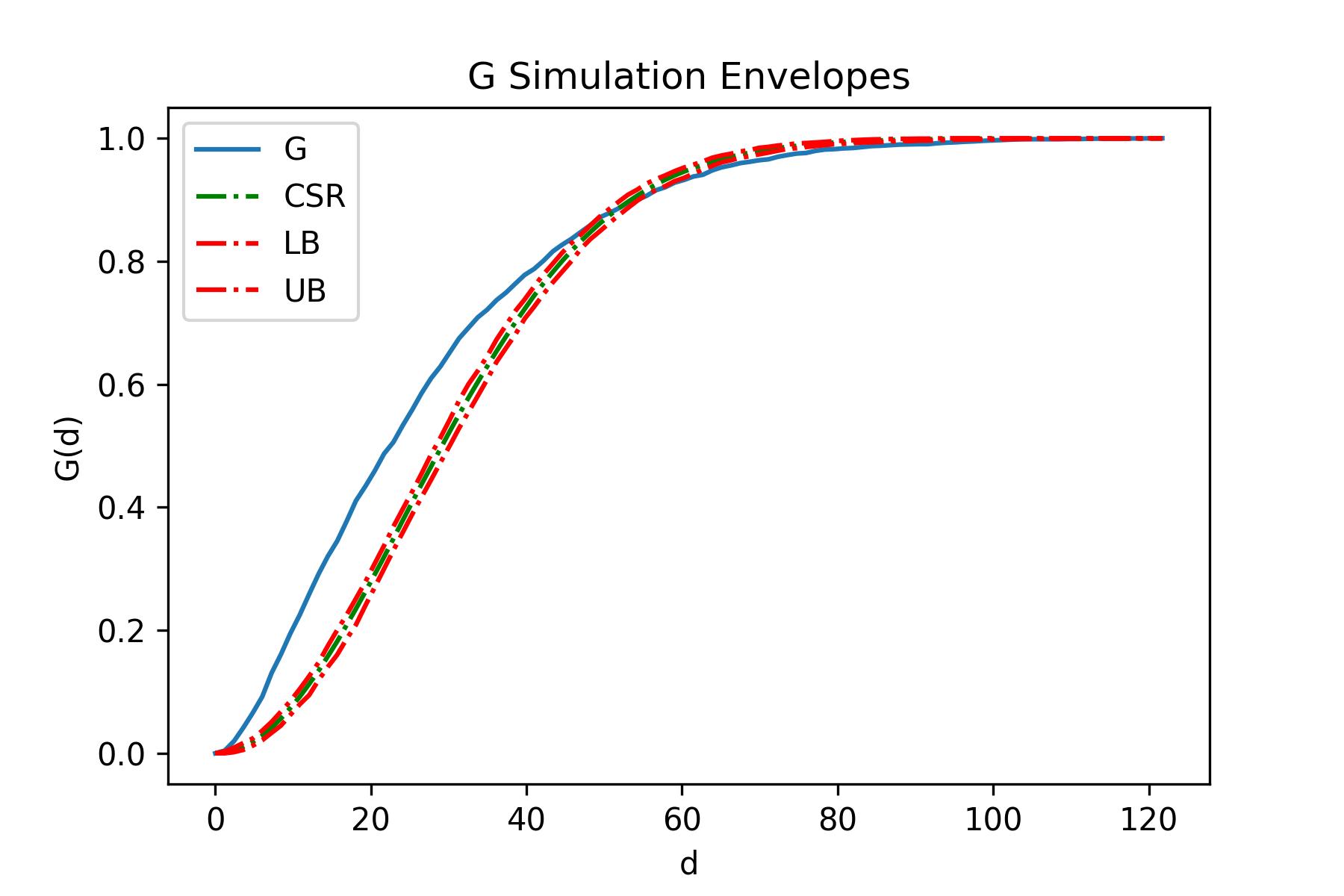

Supplement: Supplementary file 1 [file ijms-23-10435-s001.zip › supplementary File S1/STORM G/storm_btx_3.jpg]

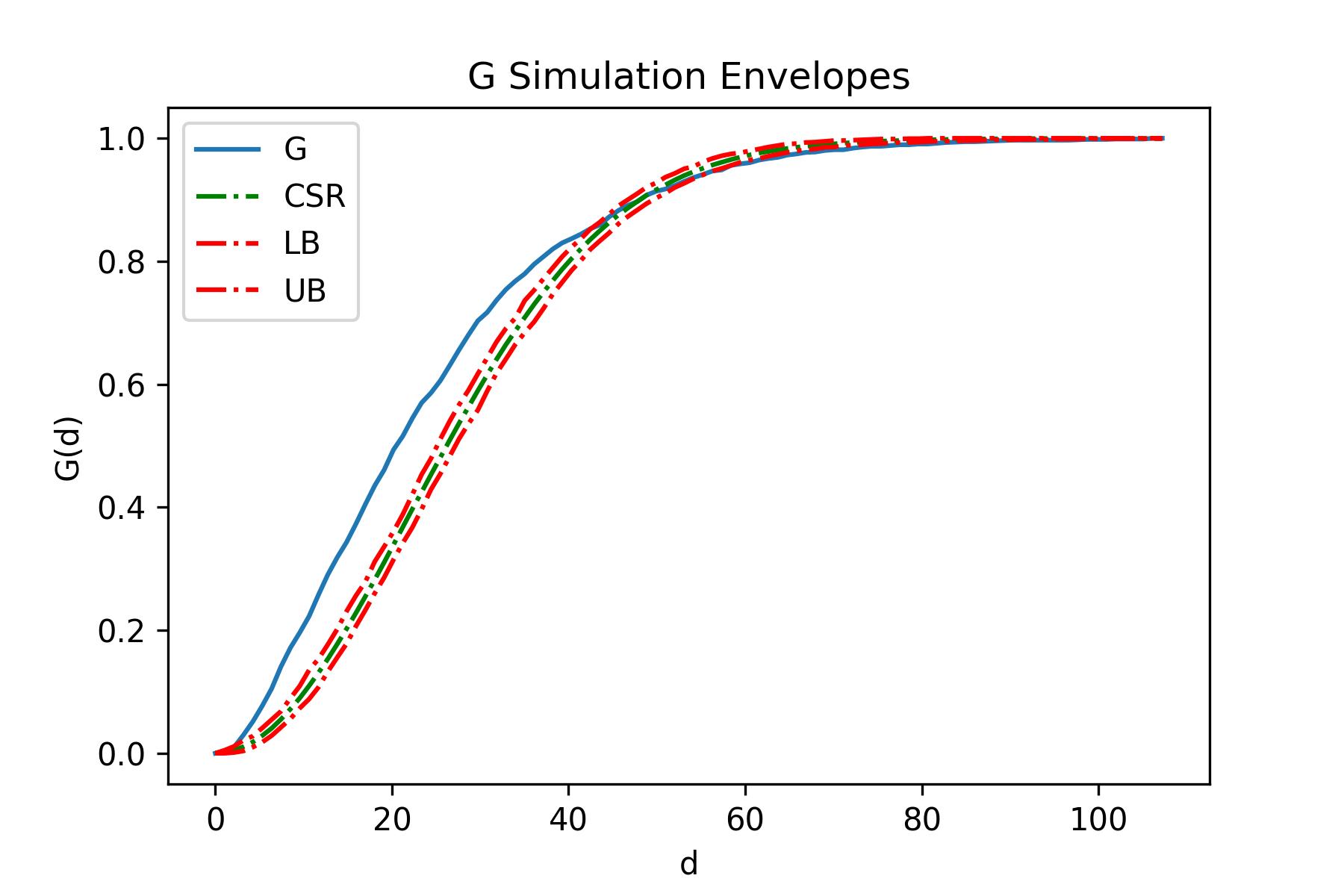

Supplement: Supplementary file 1 [file ijms-23-10435-s001.zip › supplementary File S1/STORM G/storm_btx_4.jpg]

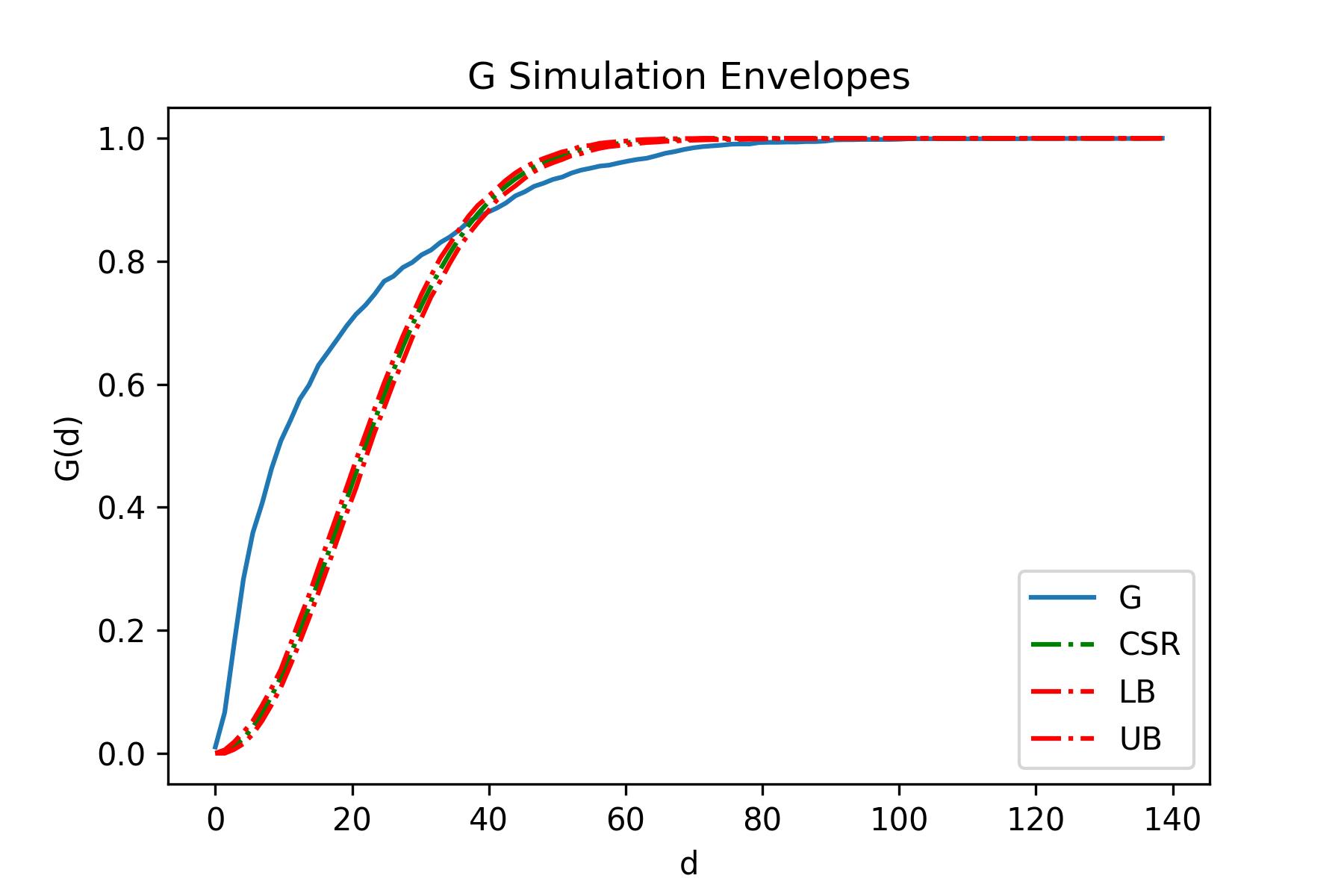

Supplement: Supplementary file 1 [file ijms-23-10435-s001.zip › supplementary File S1/STORM G/storm_btx_5.jpg]

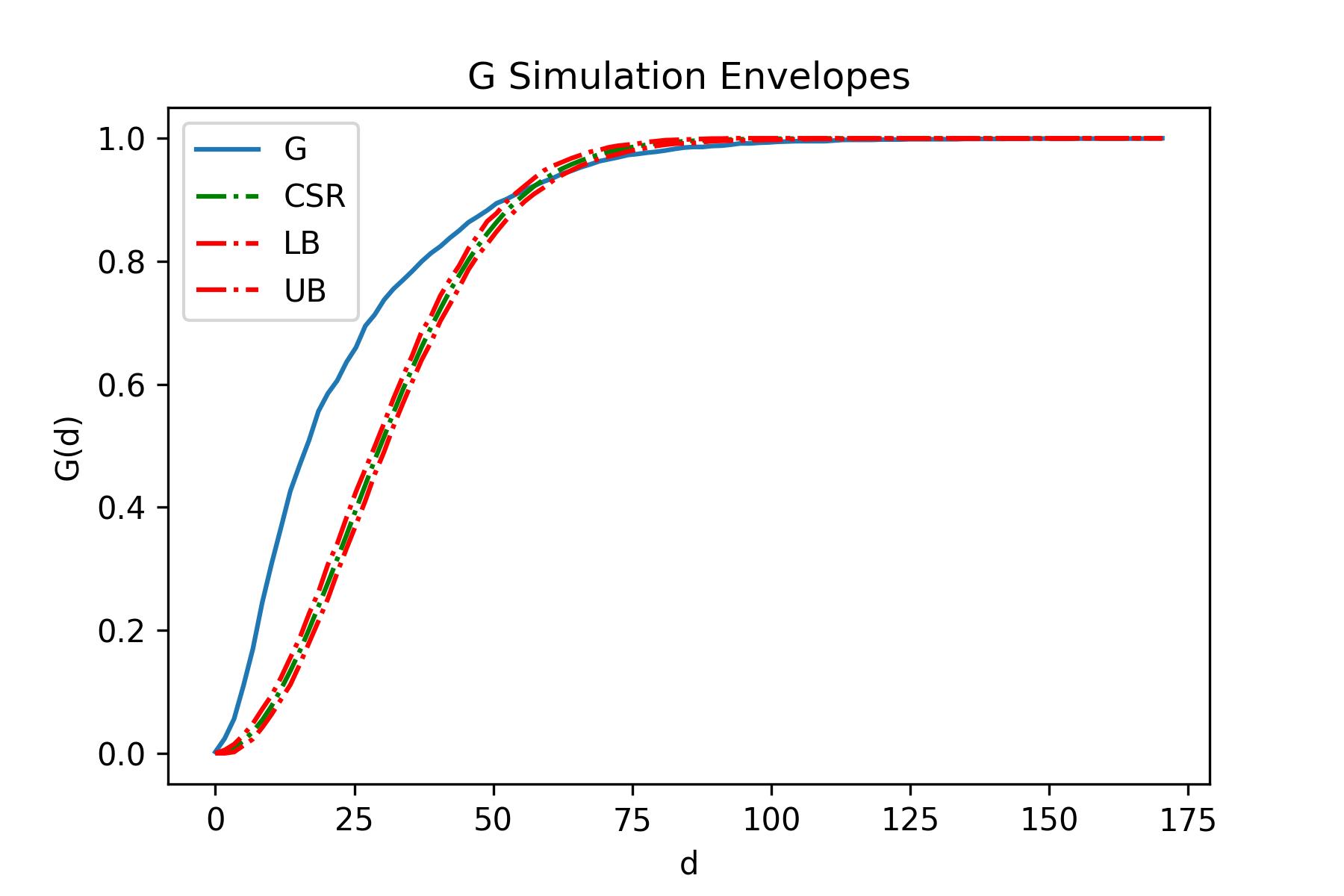

Supplement: Supplementary file 1 [file ijms-23-10435-s001.zip › supplementary File S1/STORM G/storm_btx_6.jpg]

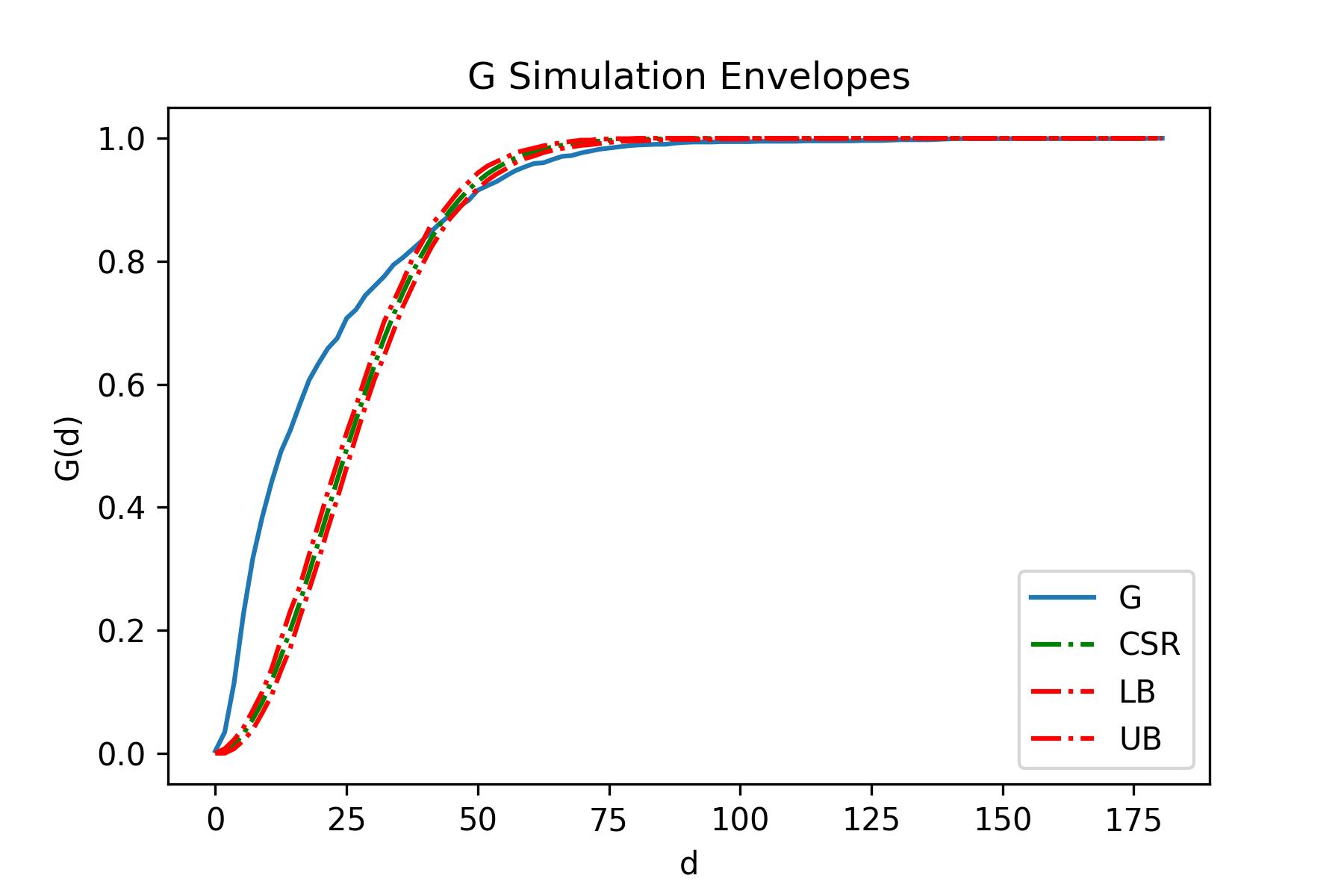

Supplement: Supplementary file 1 [file ijms-23-10435-s001.zip › supplementary File S1/STORM G/storm_btx_7.jpg]

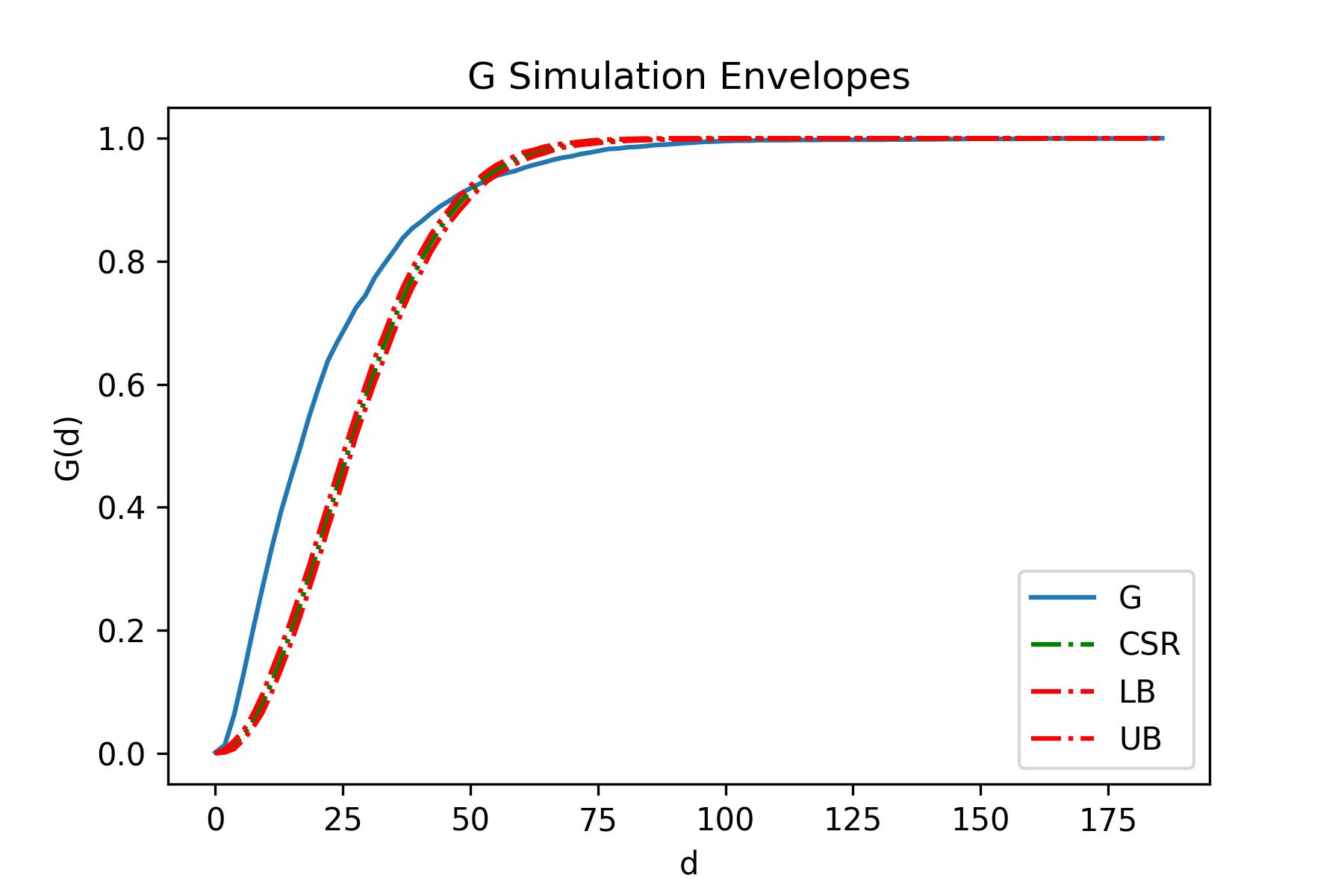

Supplement: Supplementary file 1 [file ijms-23-10435-s001.zip › supplementary File S1/STORM G/storm_btx_8.jpg]

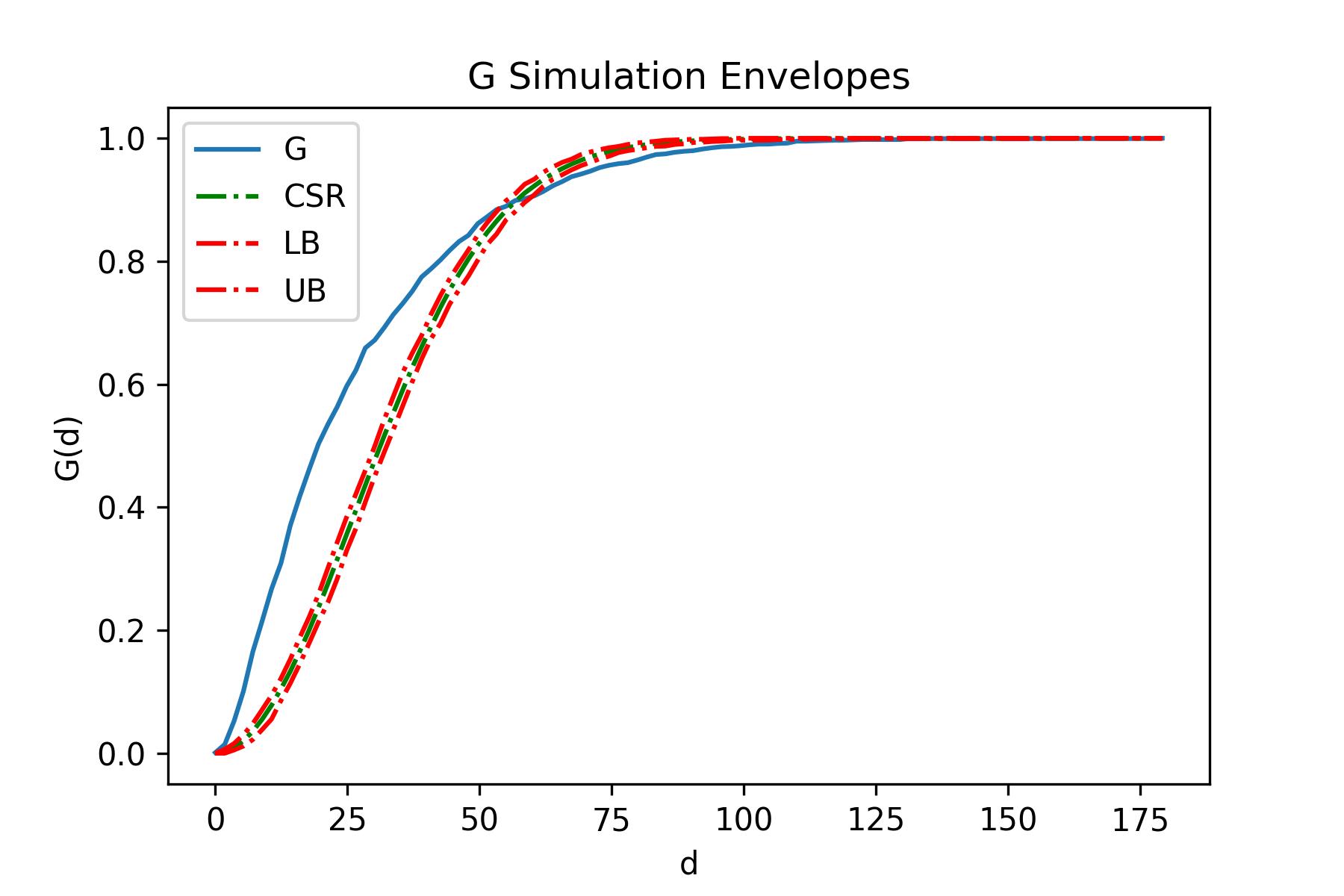

Supplement: Supplementary file 1 [file ijms-23-10435-s001.zip › supplementary File S1/STORM G/storm_btx_9.jpg]

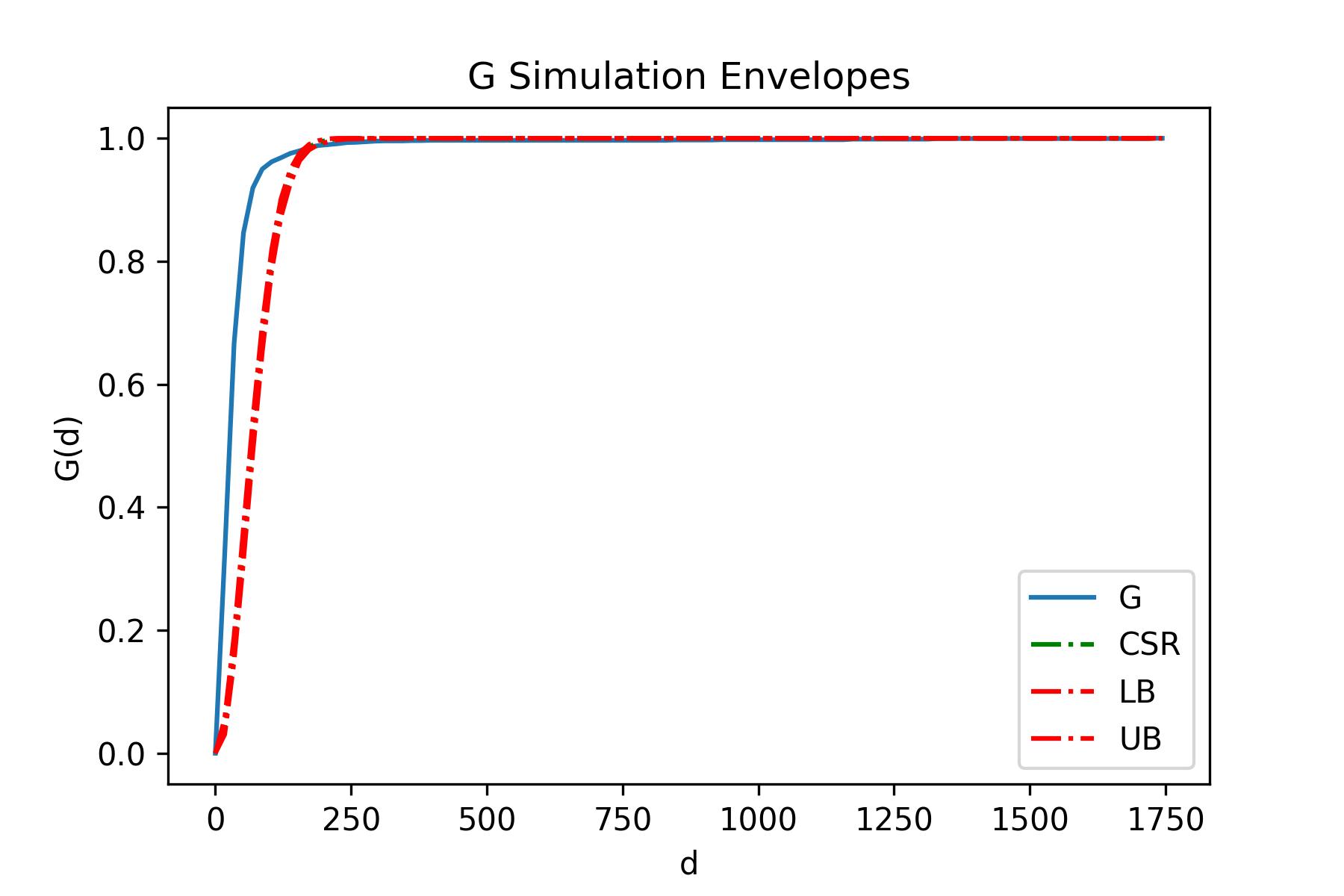

Supplement: Supplementary file 1 [file ijms-23-10435-s001.zip › supplementary File S1/STORM G/storm_mab_0.jpg]

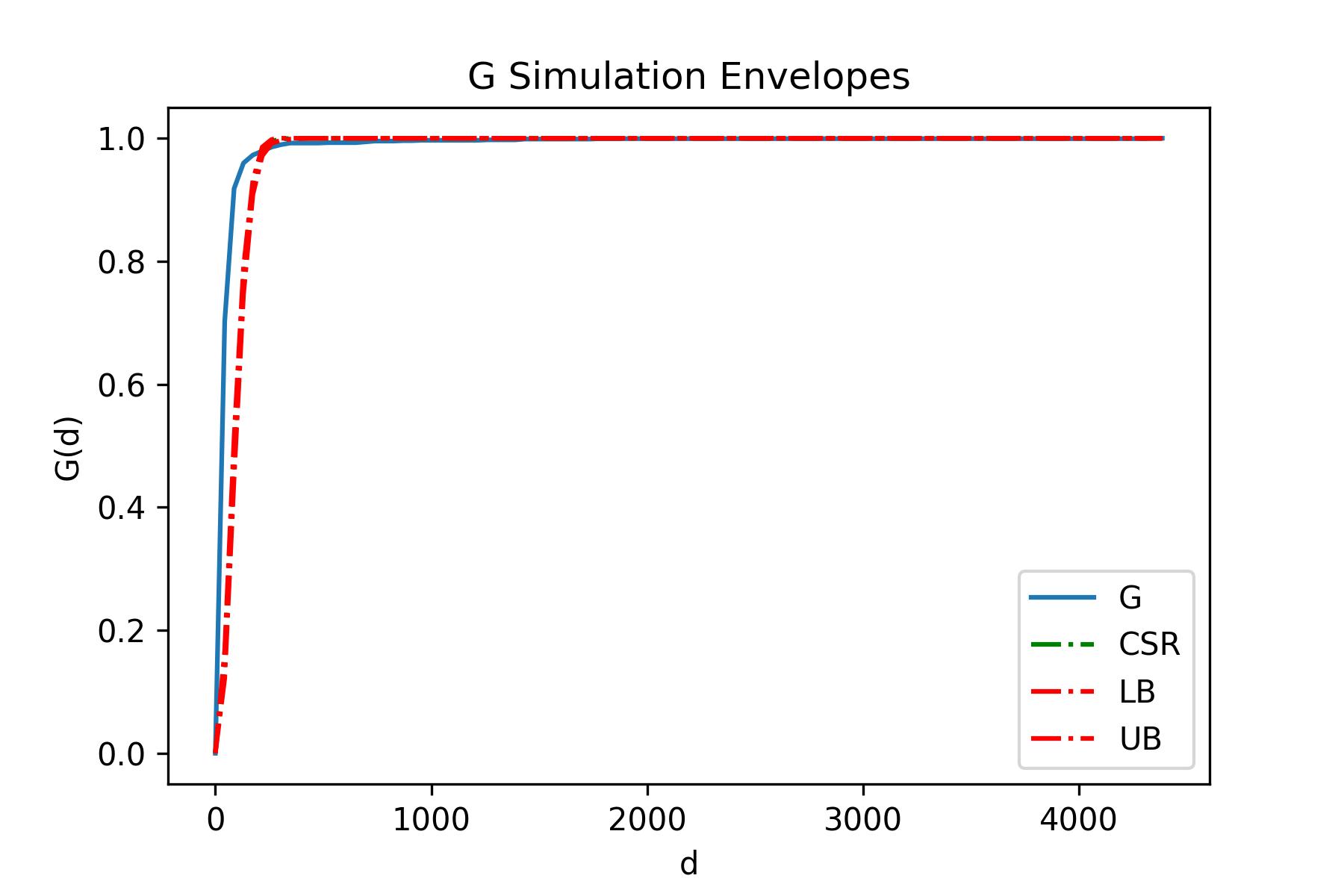

Supplement: Supplementary file 1 [file ijms-23-10435-s001.zip › supplementary File S1/STORM G/storm_mab_1.jpg]

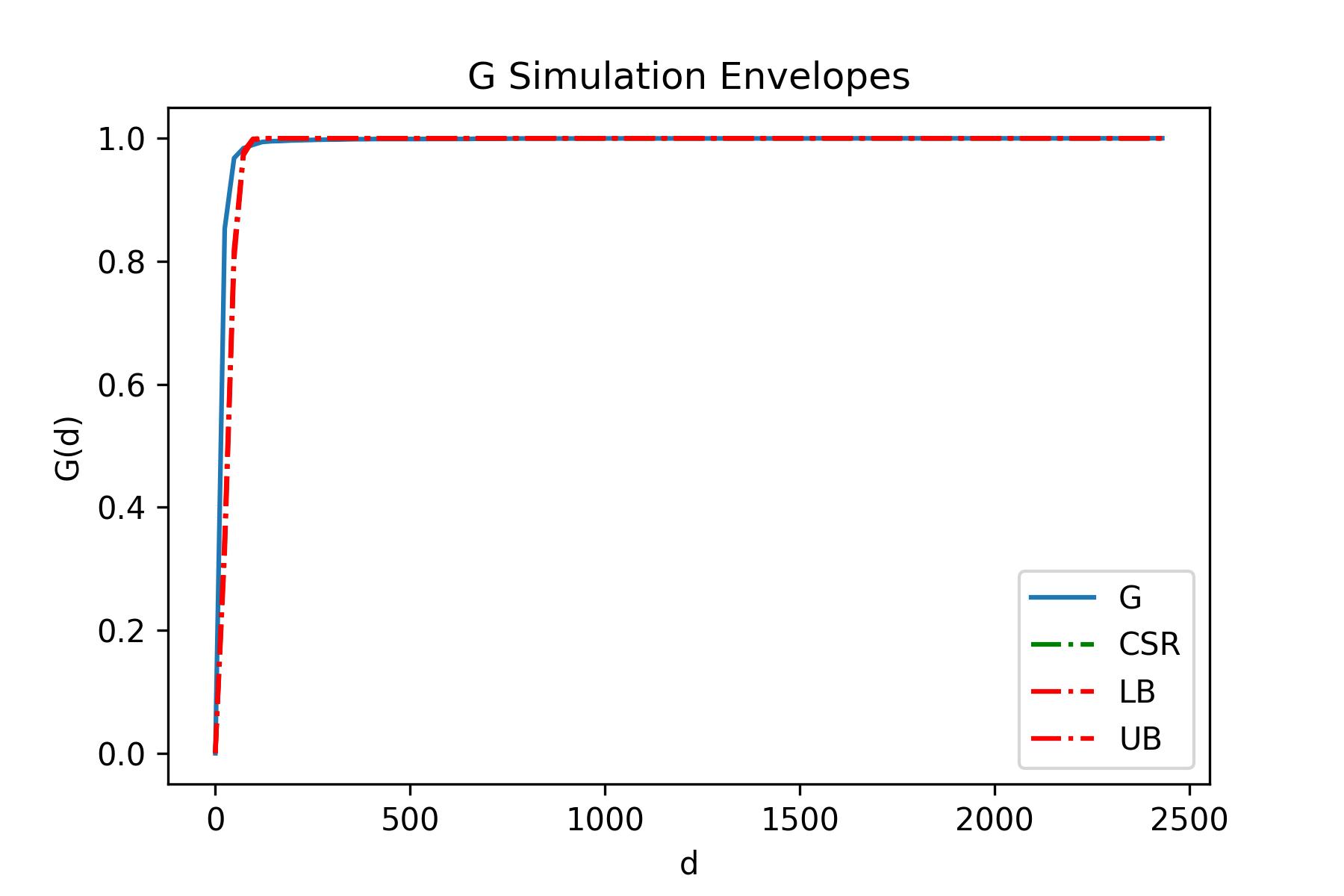

Supplement: Supplementary file 1 [file ijms-23-10435-s001.zip › supplementary File S1/STORM G/storm_mab_10.jpg]

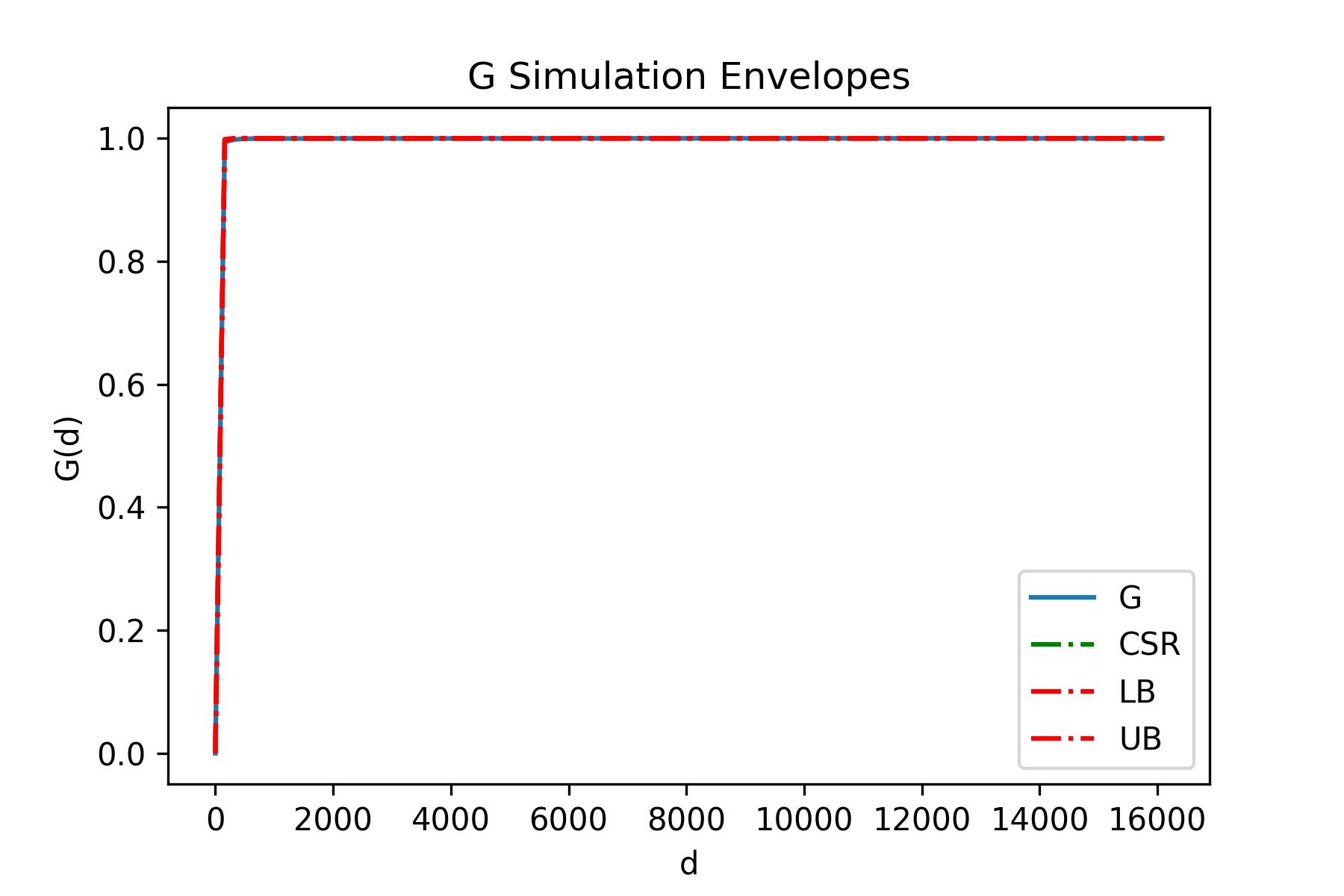

Supplement: Supplementary file 1 [file ijms-23-10435-s001.zip › supplementary File S1/STORM G/storm_mab_11.jpg]

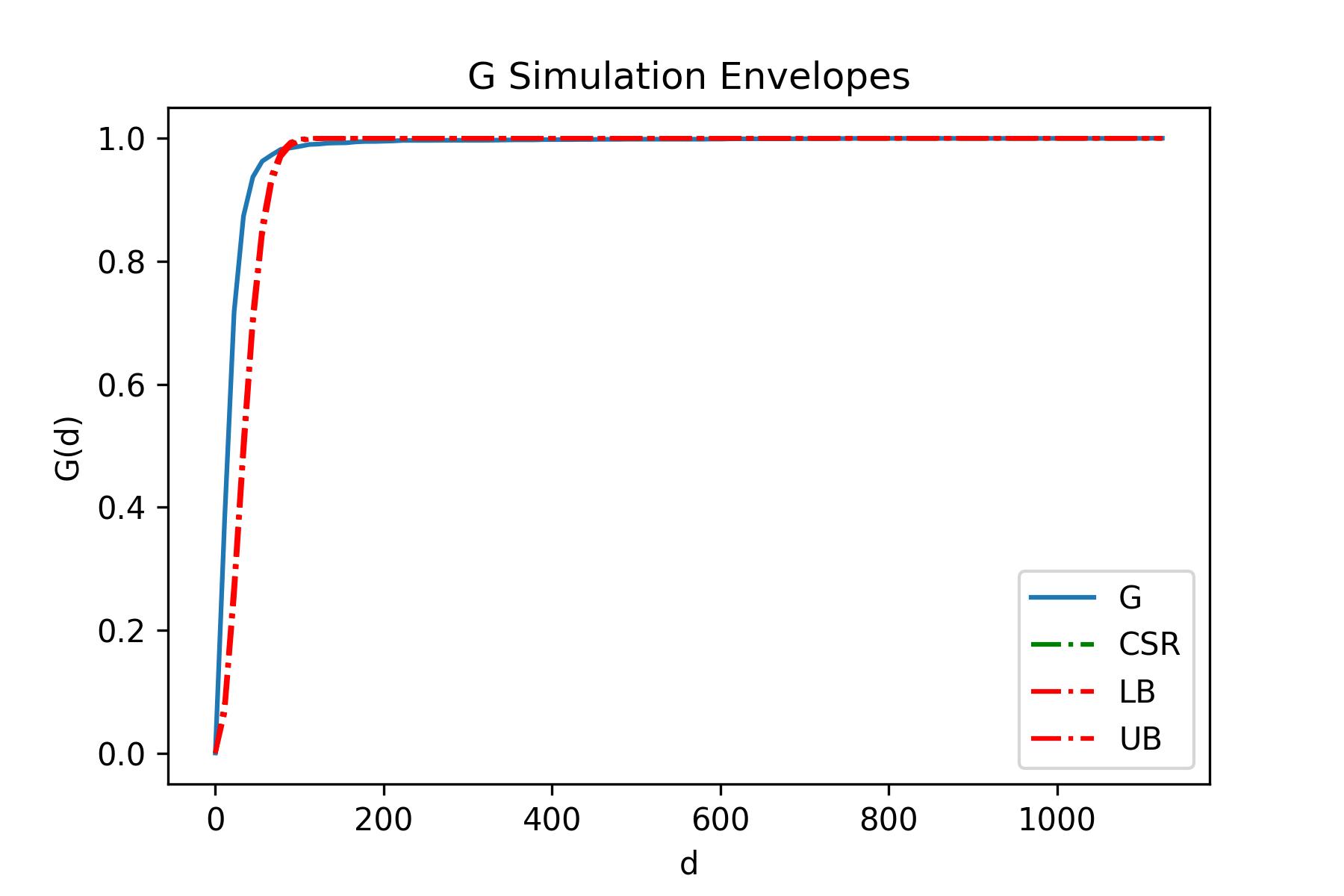

Supplement: Supplementary file 1 [file ijms-23-10435-s001.zip › supplementary File S1/STORM G/storm_mab_12.jpg]

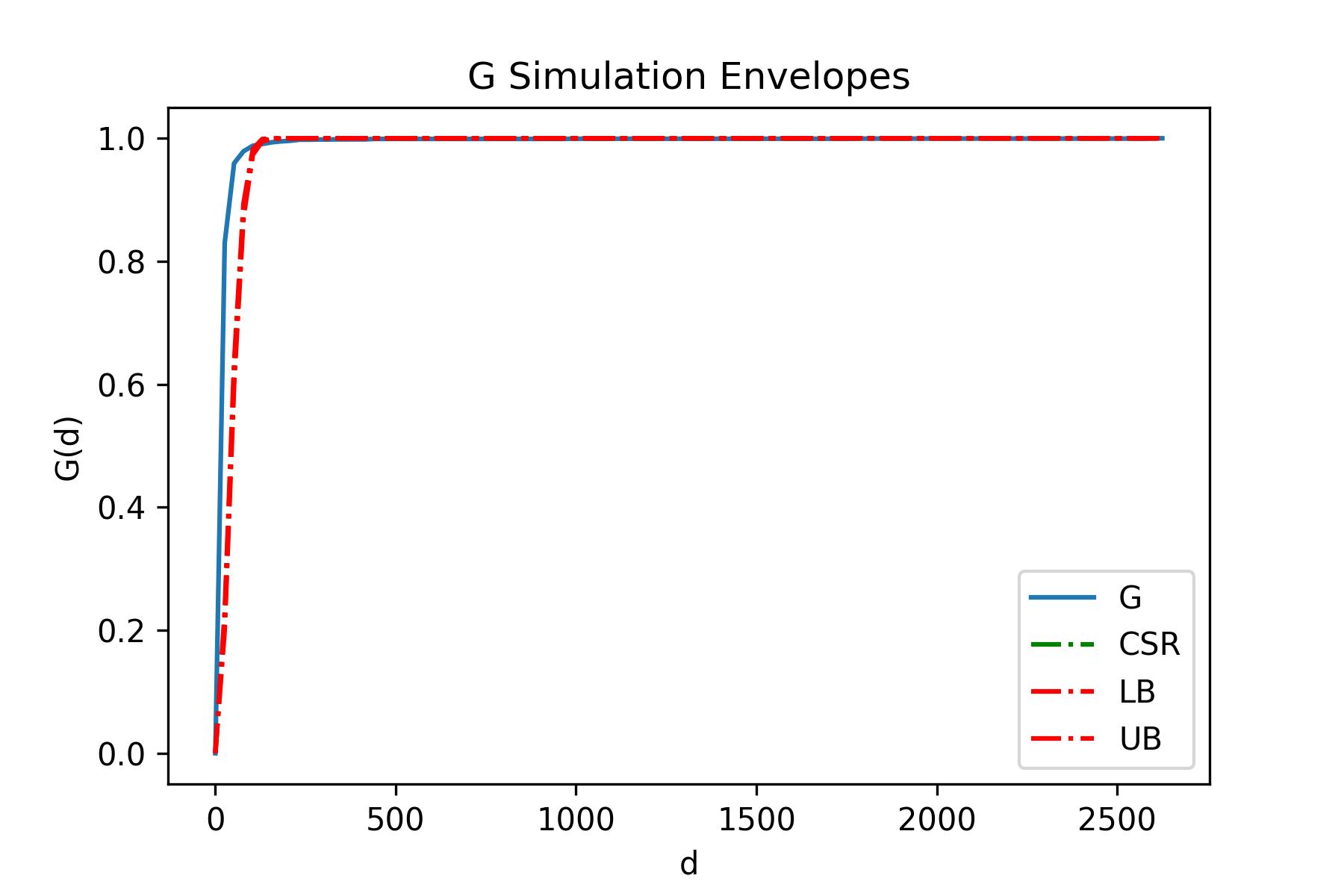

Supplement: Supplementary file 1 [file ijms-23-10435-s001.zip › supplementary File S1/STORM G/storm_mab_13.jpg]

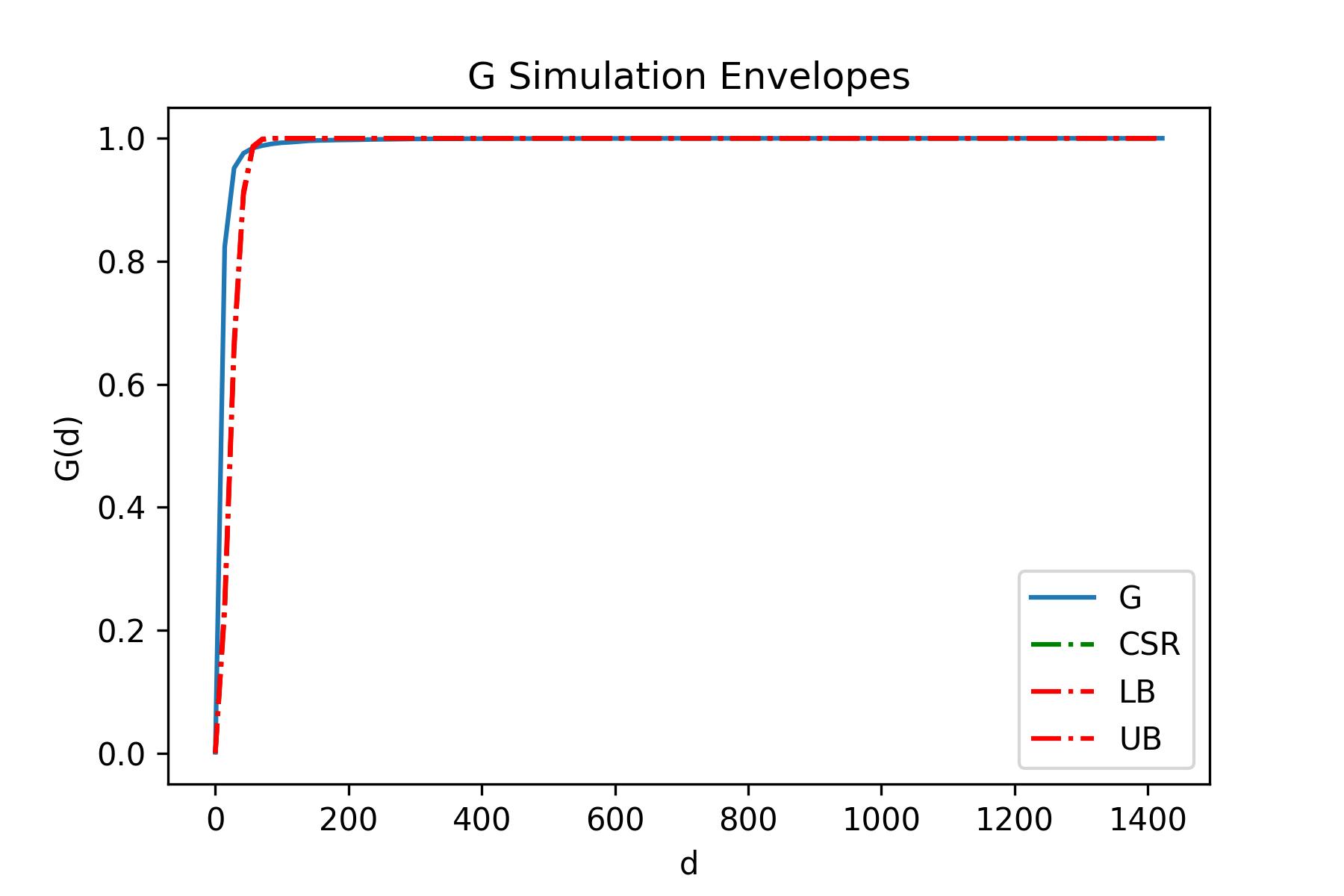

Supplement: Supplementary file 1 [file ijms-23-10435-s001.zip › supplementary File S1/STORM G/storm_mab_14.jpg]

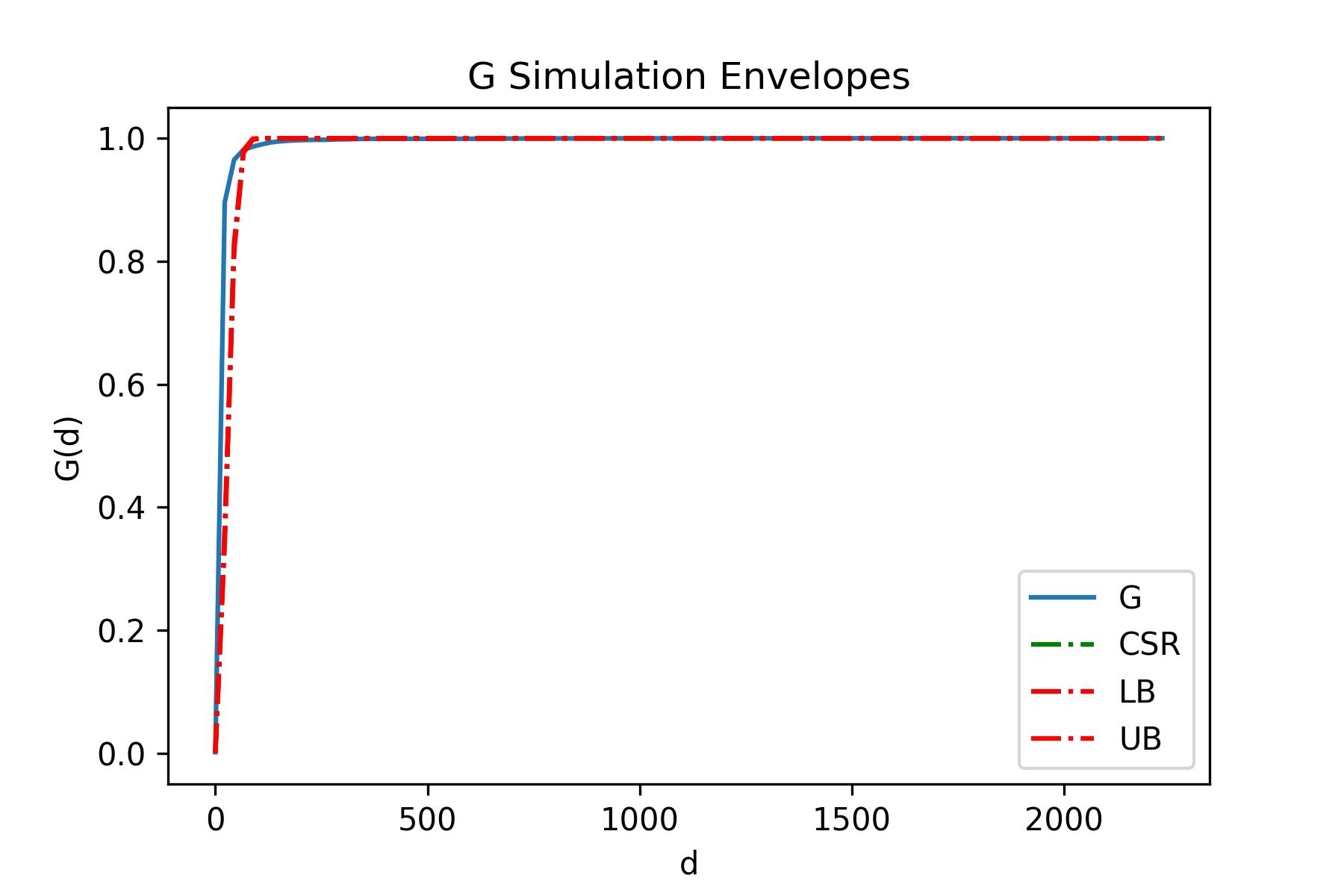

Supplement: Supplementary file 1 [file ijms-23-10435-s001.zip › supplementary File S1/STORM G/storm_mab_15.jpg]

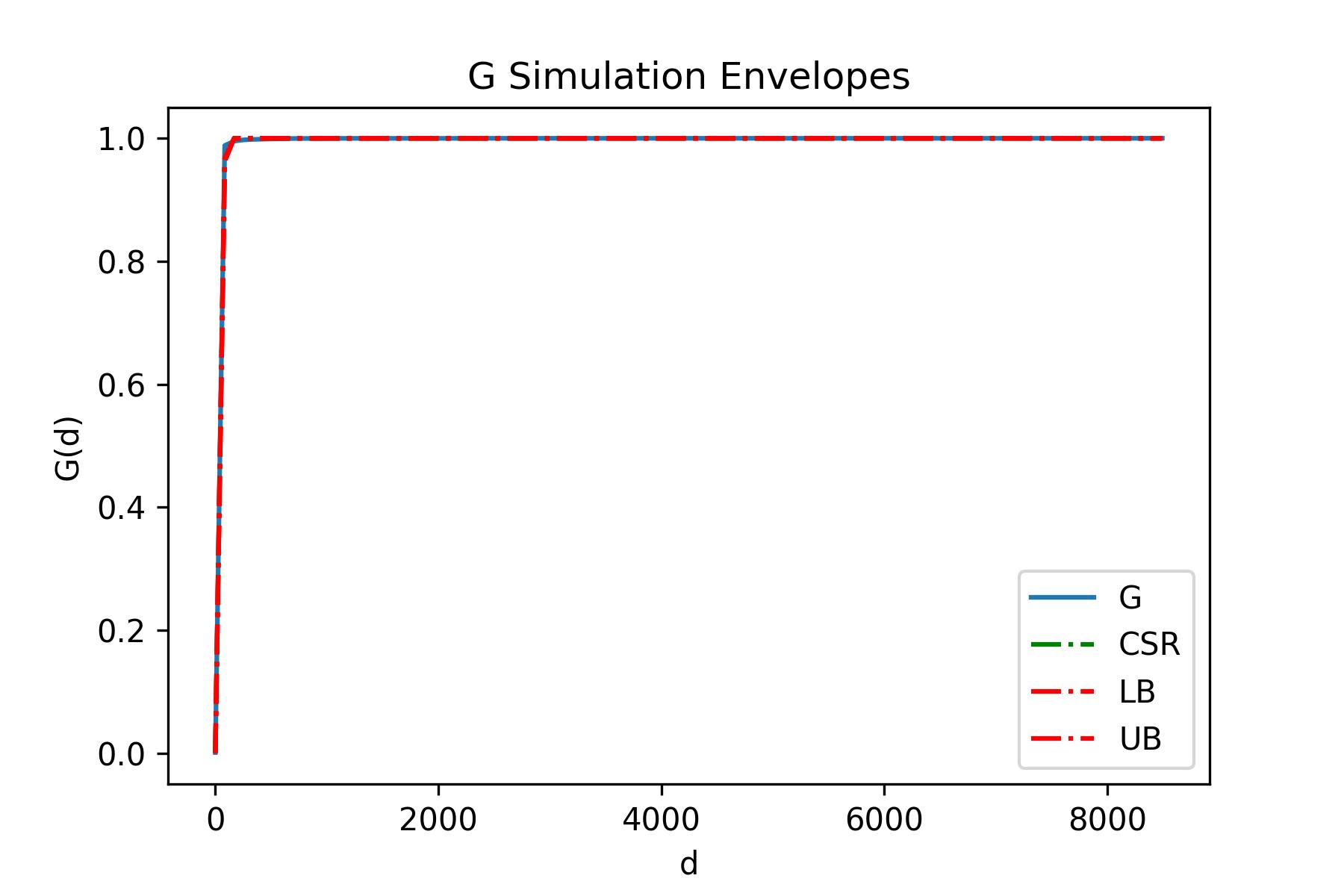

Supplement: Supplementary file 1 [file ijms-23-10435-s001.zip › supplementary File S1/STORM G/storm_mab_16.jpg]

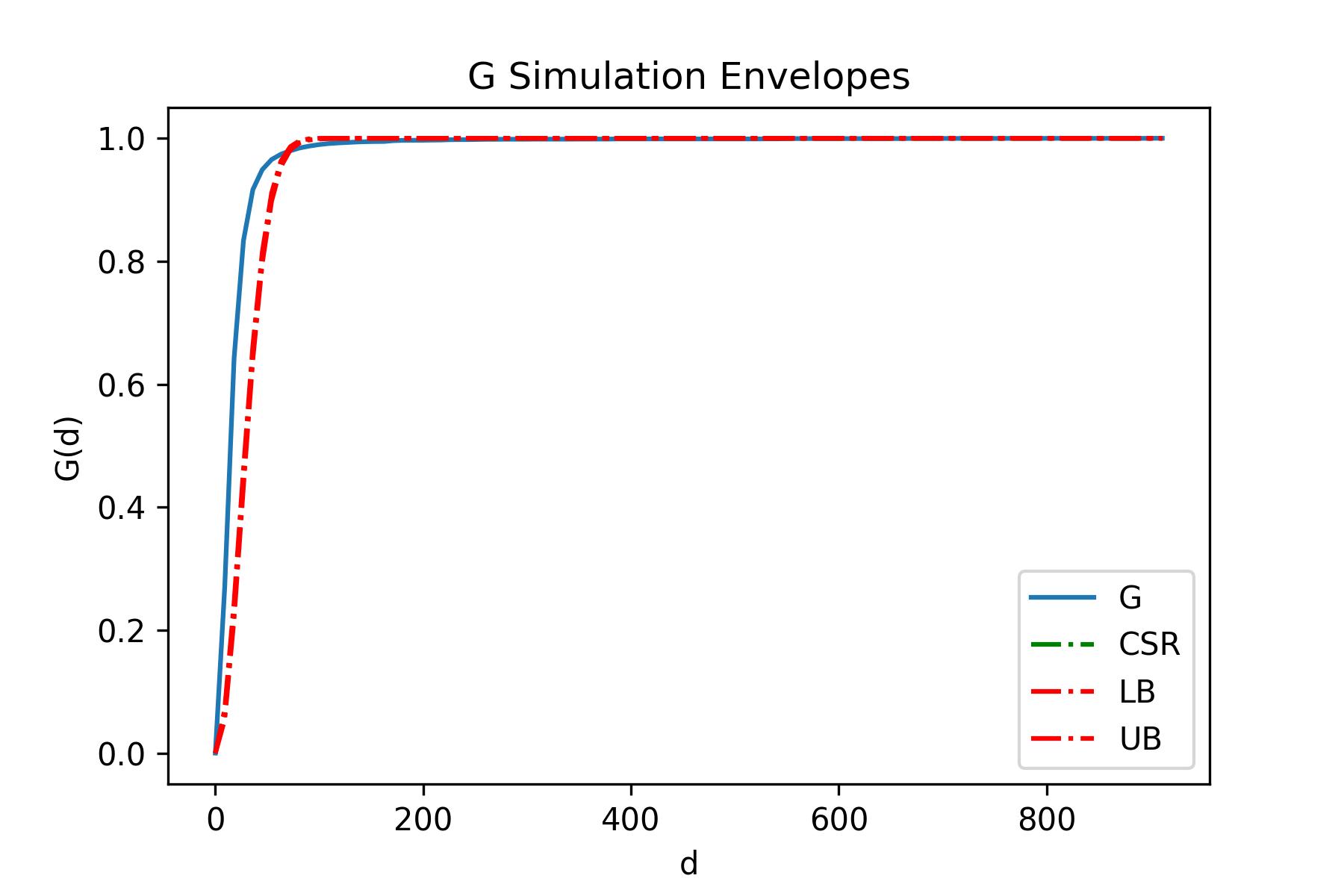

Supplement: Supplementary file 1 [file ijms-23-10435-s001.zip › supplementary File S1/STORM G/storm_mab_17.jpg]

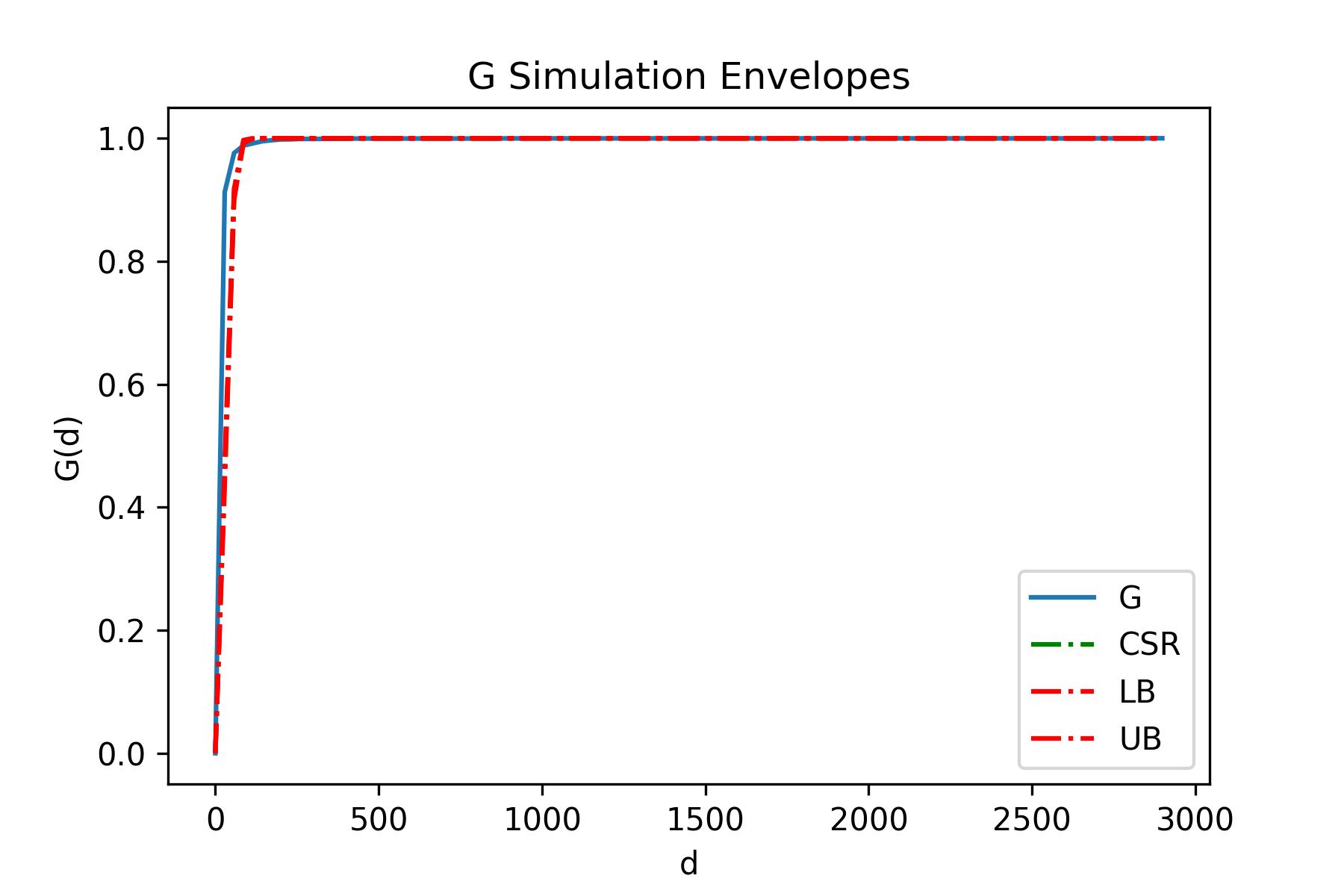

Supplement: Supplementary file 1 [file ijms-23-10435-s001.zip › supplementary File S1/STORM G/storm_mab_18.jpg]

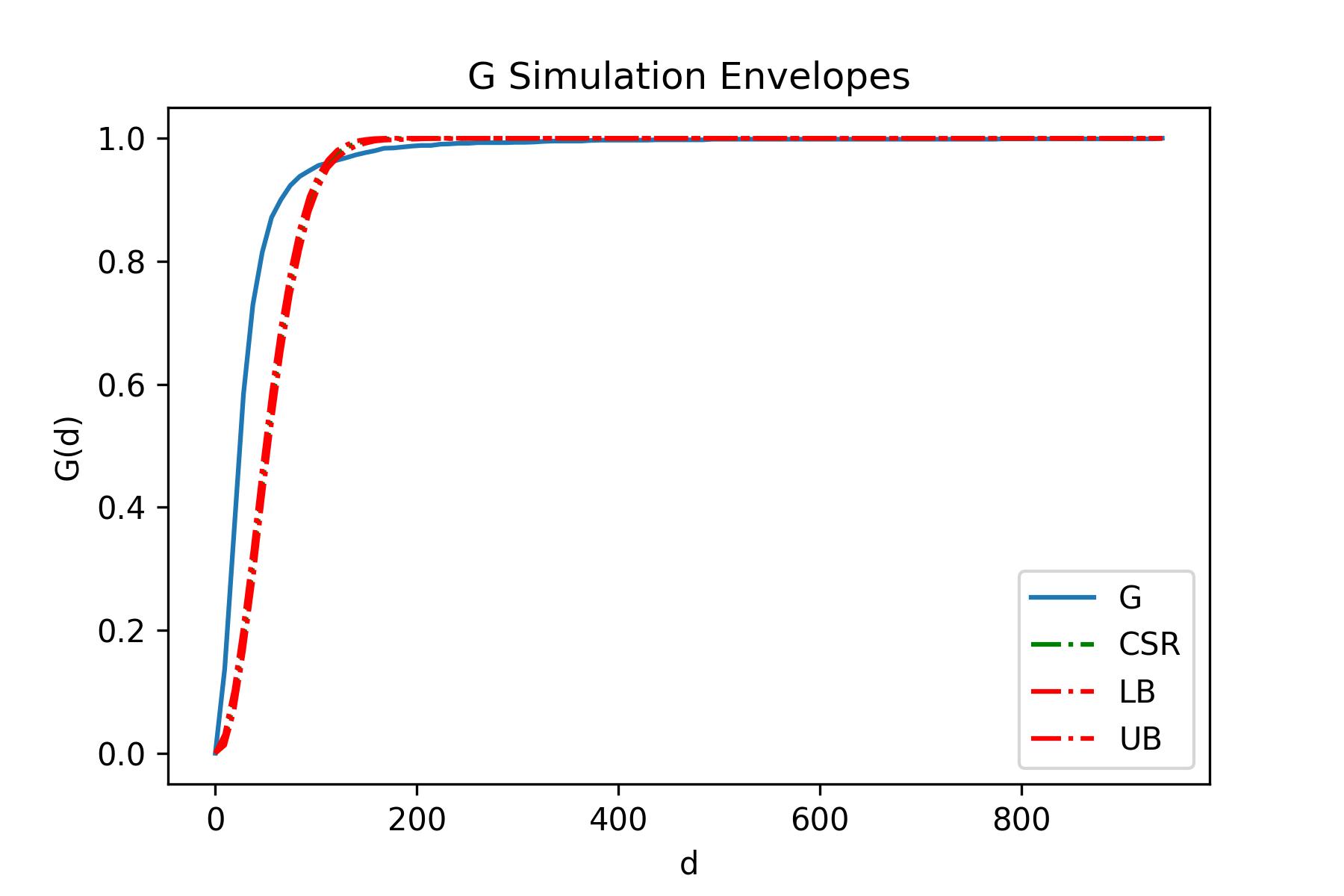

Supplement: Supplementary file 1 [file ijms-23-10435-s001.zip › supplementary File S1/STORM G/storm_mab_2.jpg]

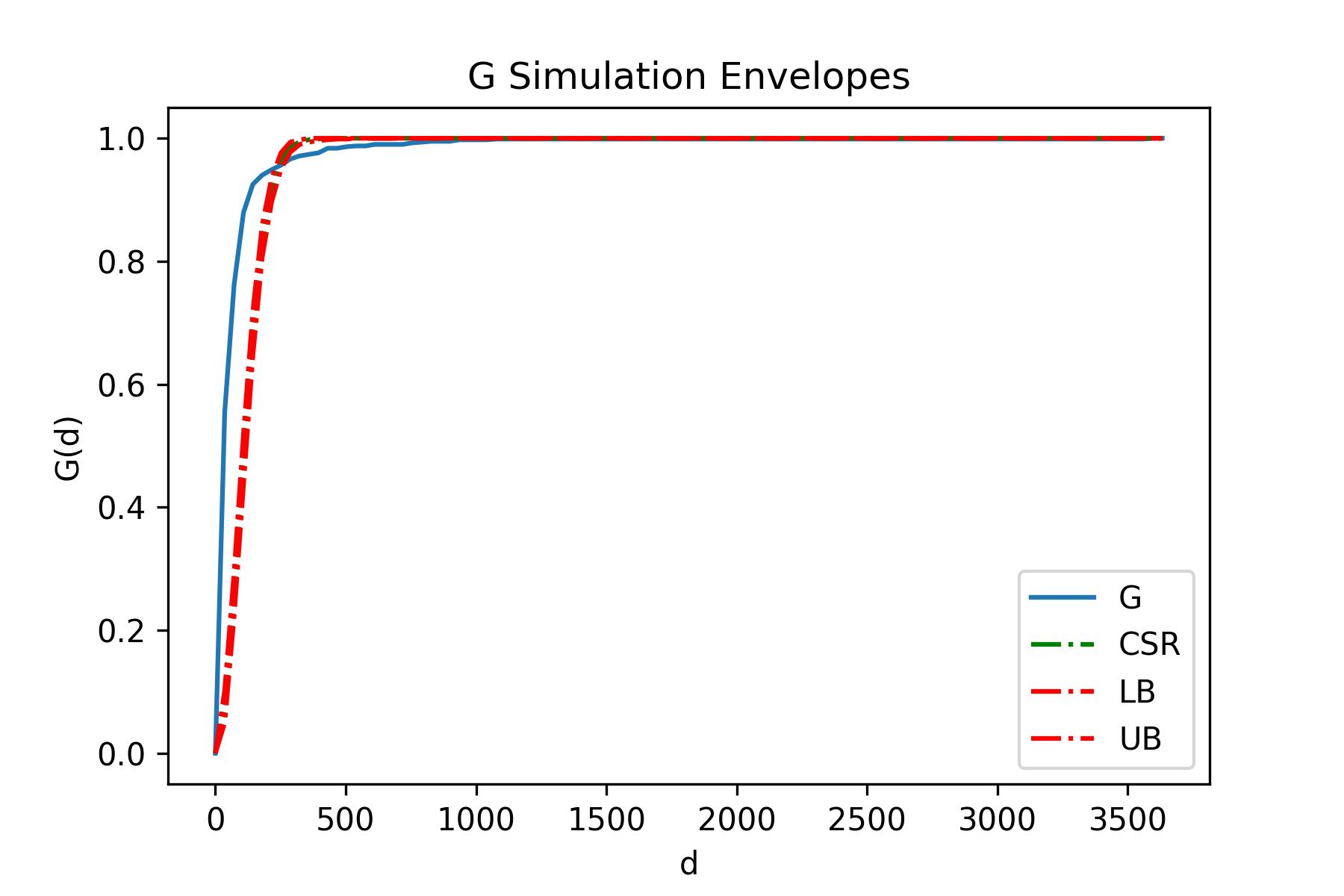

Supplement: Supplementary file 1 [file ijms-23-10435-s001.zip › supplementary File S1/STORM G/storm_mab_3.jpg]

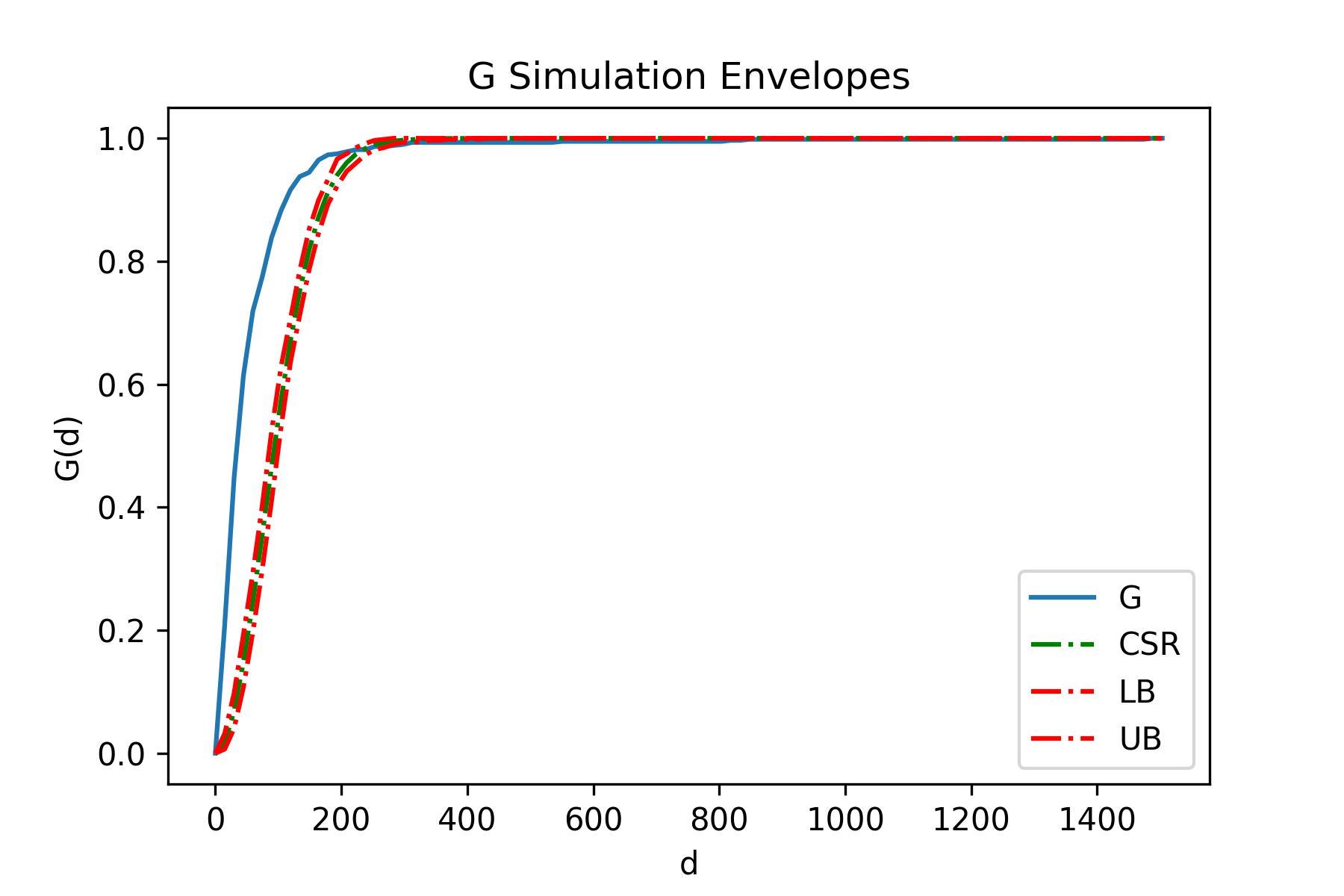

Supplement: Supplementary file 1 [file ijms-23-10435-s001.zip › supplementary File S1/STORM G/storm_mab_4.jpg]

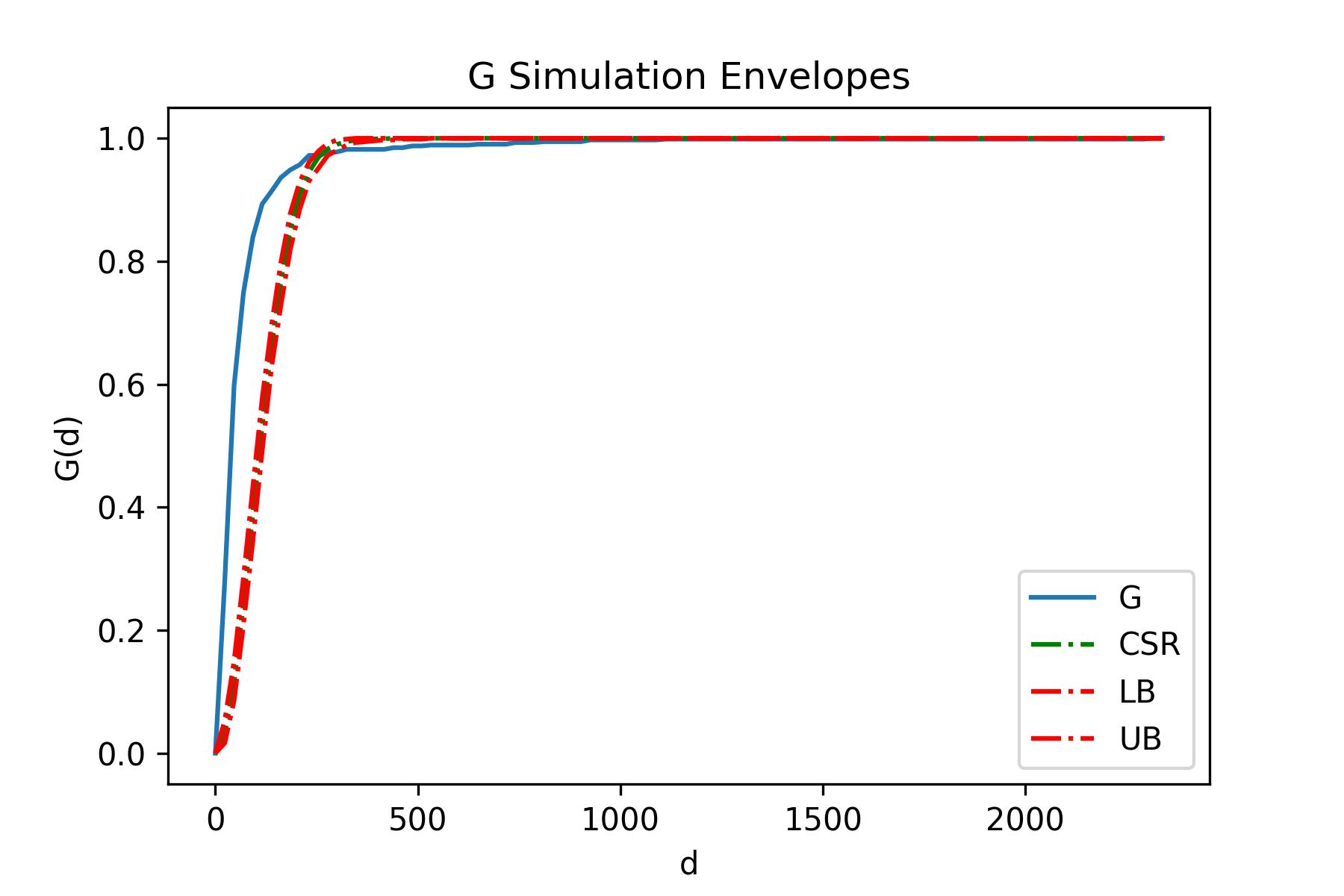

Supplement: Supplementary file 1 [file ijms-23-10435-s001.zip › supplementary File S1/STORM G/storm_mab_5.jpg]

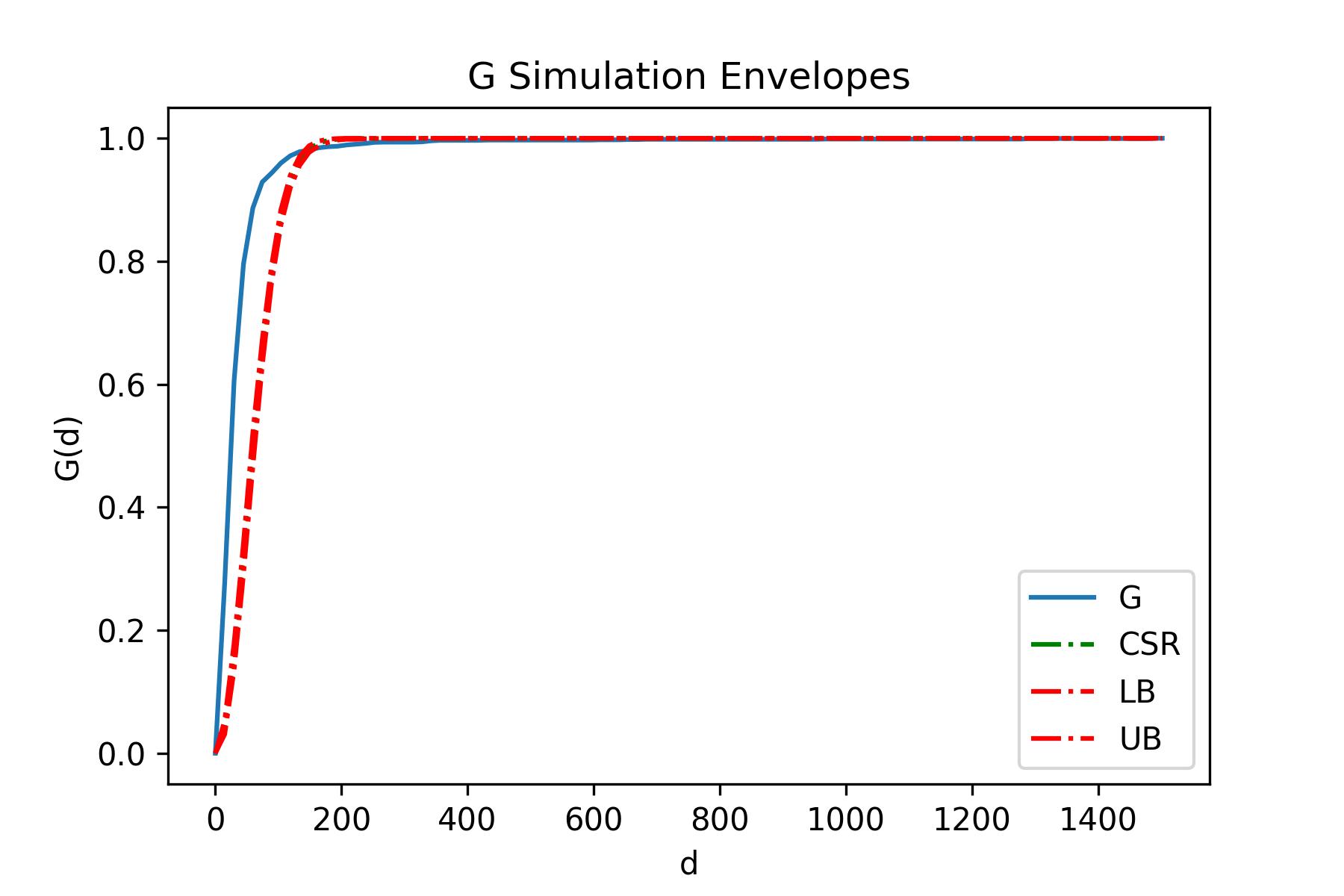

Supplement: Supplementary file 1 [file ijms-23-10435-s001.zip › supplementary File S1/STORM G/storm_mab_6.jpg]

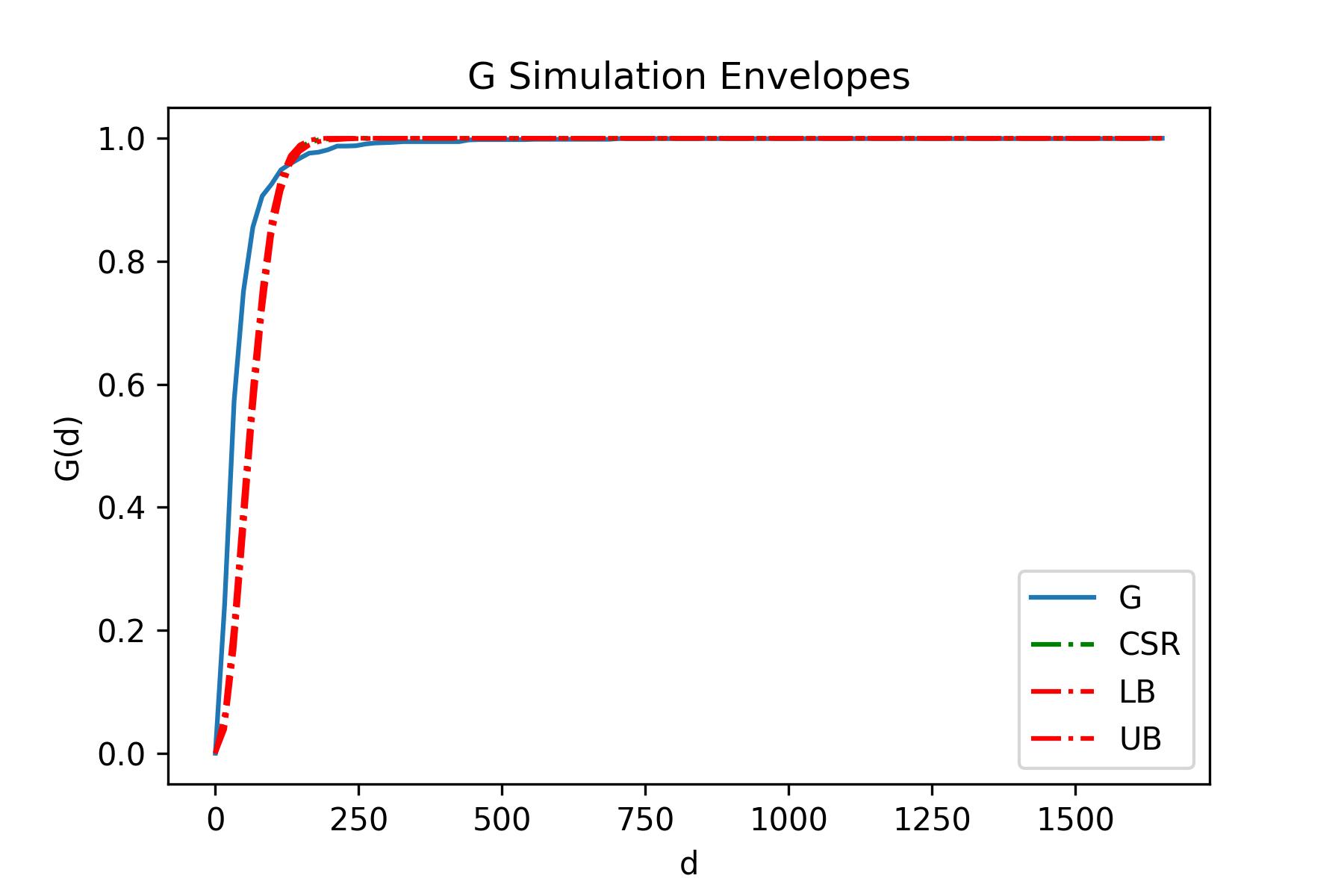

Supplement: Supplementary file 1 [file ijms-23-10435-s001.zip › supplementary File S1/STORM G/storm_mab_7.jpg]

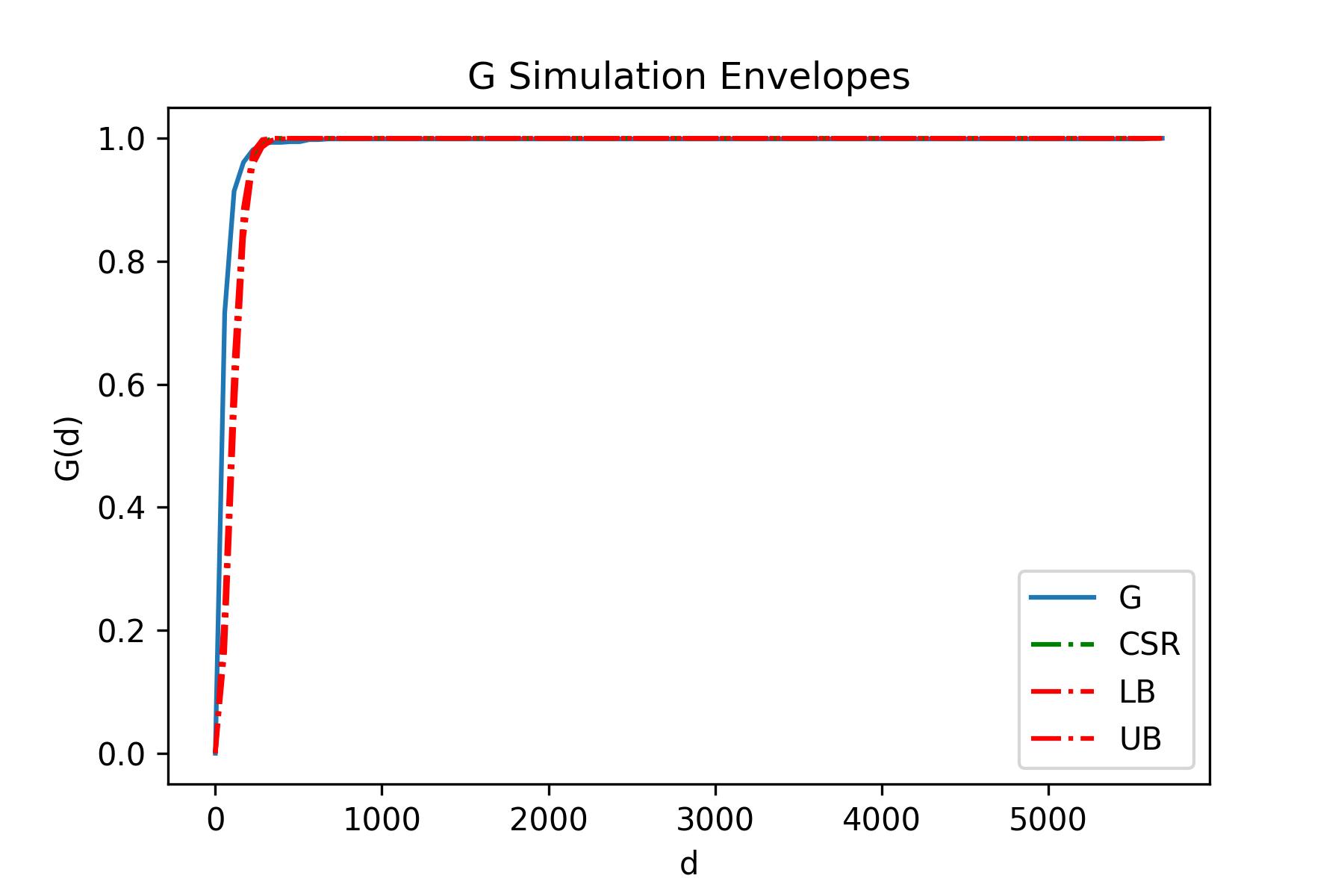

Supplement: Supplementary file 1 [file ijms-23-10435-s001.zip › supplementary File S1/STORM G/storm_mab_8.jpg]

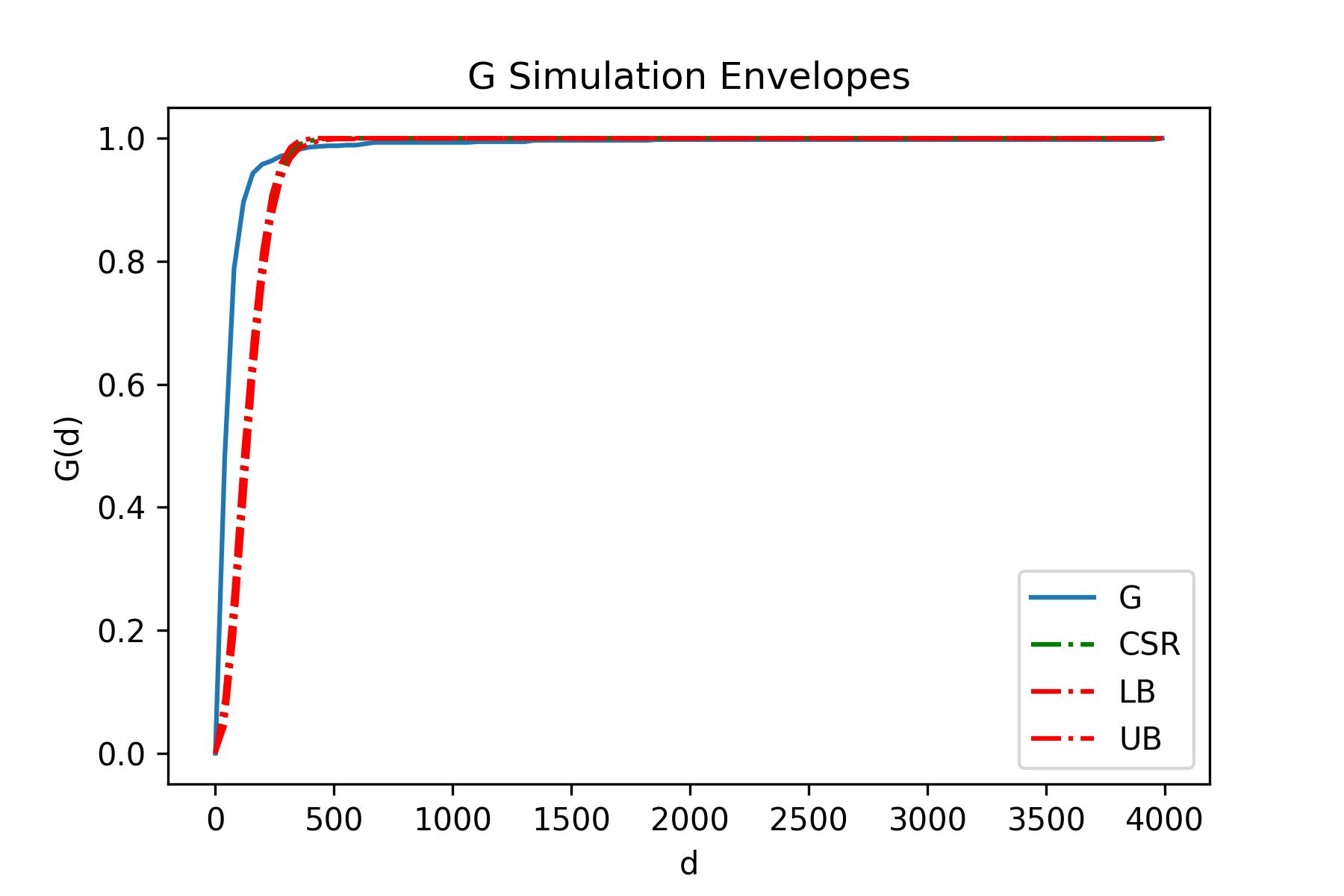

Supplement: Supplementary file 1 [file ijms-23-10435-s001.zip › supplementary File S1/STORM G/storm_mab_9.jpg]
